# Design, synthesis and biological evaluation of a series of dianilinopyrimidines as EGFR inhibitors

**DOI:** 10.1080/14756366.2022.2046567

**Published:** 2022-03-09

**Authors:** Longjia Yan, Qin Wang, Li Liu, Yi Le

**Affiliations:** a School of Pharmaceutical Sciences, Guizhou University, Guiyang, China; b State Key Laboratory of Functions and Applications of Medicinal Plants, Guizhou Medical University, Guiyang, China; c Guizhou Engineering Laboratory for Synthetic Drugs, Guiyang, China

**Keywords:** Design, synthesis, EGFR, inhibitor, antitumor

## Abstract

This paper described our efforts to develop dianilinopyrimidines as novel EGFR inhibitors. All the target compounds were tested for inhibitory effects against wild type EGFR (EGFR^wt^) and three tumour cells, including A549, PC-3, and HepG2. Some of the compounds performed well in antitumor activities. Especially, compound **4c** 2-((2-((4-(3-fluorobenzamido)phenyl)amino)-5-(trifluoromethyl) pyrimidin-4-yl)amino)-*N*-methylthiophene-3-carboxamide showed higher anti-tumour activities than Gefitinib. The IC_50_ values of compound **4c** against A549, PC-3, and HepG2. reached 0.56 μM, 2.46 μM, and 2.21 μM, respectively. In addition, further studies indicated that compound **4c** could induce apoptosis against A549 cells and arrest A549 cells in the G2/M phase. Molecular docking studies showed that compound **4c** could closely interact with EGFR. Generally, compound **4c** was the potential for developing into an anti-tumour drug.

## 1. Introduction

Lung cancer is an incurable respiratory disease. It is one of the fastest-growing malignant tumours with incidence and mortality rate worldwide, which seriously endangers human life and health^1–4^. Among the patients with lung cancer, they were diagnosed more than 75% as non-small cell lung cancer (NSCLC). Moreover, the five-year survival rate of patients with NSCLC is very low^5–7^. Many studies have shown that the epidermal growth factor receptor (EGFR) tyrosine kinase was one of the critical targets for treating NSCLC^8–10^.

**Figure 1.**
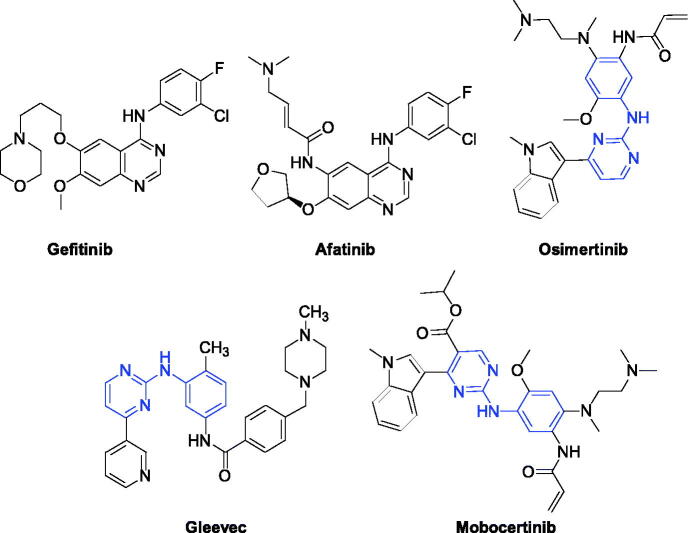
Anti-tumour drugs in the market.

EGFR is a receptor for an epithelial growth factor (EGF) cell proliferation and signal transduction^11^. It belongs to a family of ErbB receptors, which includes EGFR (HER1 or ErbB-1), HER2 (ErbB-2), HER3 (ErbB-3) and HER4 (ErbB-4). EGFR plays an essential role in regulating cell growth, proliferation and differentiation and other physiological activities of various cancer cells, which is an important target for anti-cancer drug research^12–14^. As shown in Figure 1, lots of EGFR inhibitors such as Gefitinib, Afatinib, and Osimertinib have been approved in the market, which significantly improves the clinical treatment of NSCLC patients^15–17^. However, with the continuously emerging resistance of EGFR inhibitors, the development of new EGFR inhibitors has become a hot topic in drug discovery^18–20^.

**Figure 2.**
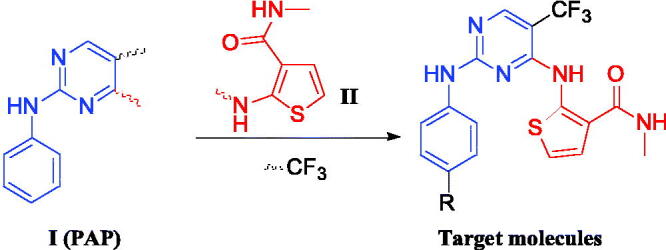
The design strategy of EGFR inhibitors.

Tremendous researches indicated that phenylaminopyrimidine (PAP) derivatives were important for new drug design^21^^,^^22^. Many anti-tumour reagents contained the fragment of PAP, such as Gleevec^23^, Osimertinib^24^, and Mobocertinib (Figure 1)^25^ In addition, a large number of molecules containing the structure of PAP (Blue colour in Figure 1) are in the stage of clinical research^26–28^. To develop new anti-tumour reagents for the treatment of NSCLC, we are very interested in designing and synthesising new EGFR inhibitors. Our strategy is shown in Figure 2. Based on good anti-tumour activities of PAP, we were using 2-phenylaminopyrimidine (**I**, Figure 2) as the main skeleton and introducing aminothiophen moiety (**II**, Figure 2) into the 4-position of pyrimidine ring, which has been proved as the well bioactive backbone in many antimicrobial or anti-tumour reaents^29^^,^^30^. This paper will report our progress in 5-trifluoromethylpyrimidine derivatives bearing 2-aminothiophen moiety as EGFR inhibitors.

## 2. Experimental section

All chemical reagents were commercially available in Energy Chemical Reagent Co., Ltd. The NMR spectra were recorded on Bruker (Avance) 400 MHz and JEOL (Japan) 500 MHz NMR instrument with chemical reported as δ in CDCl_3_ and DMSO-d_6_, tetramethylsilane (TMS) as the internal standard. The high-resolution mass spectrometer (HRMS) was tested in TSQ 8000 and AB SCIEX X500R QTOF. Elemental analysis was recorded in vario EL Cube (Elementar, Germany).

### 2.1. Synthesis

#### 2.1.1. 2-(2-Chloro-5-trifluoromethyl-pyrimidin-4-ylamino)-thiophene-3-carboxylic acid methylamide (1)

2,4-Dichloro-5-trifluoromethylpyrimidine (10 mmol) was added to a stirred solution of 2-amino-*N*-methylthiophene-3-carboxamide (11 mmol) and NaHCO_3_ (11 mmol) in anhydrous EtOH (20 mL) at room temperature. The resulted mixture was heated to reflux and stirred overnight before cool at room temperature. The precipitate was filtered out, washed with water to give the compound as yellow solid (1.344 g; 40% yield). mp: 136.3–138.2 °C; ^1^H NMR (400 MHz, DMSO-d_6_) δ 13.68 (s, 1H), 8.75 (s, 1H), 8.59 (q, *J* = 4.8 Hz, 1H), 7.51 (d, *J* = 6.0 Hz, 1H), 7.19 (d, *J* = 6.0 Hz, 1H), 2.82 (d, *J* = 4.4 Hz, 3H); ^13 ^C NMR (100 MHz, DMSO-d_6_) δ 166.1, 162.1, 157.1, 153.3, 144.8, 124.9, 122.9, 119.2, 116.7, 107.5, 26.3; ESI-HRMS C_11_H_8_ClF_3_N_4_OS ([M + Na]^+^): calcd 358.9951, found 358.9949. HMBC experiment, a correlation was observed between the C^4^-*N*-proton at 13.68 ppm and the C^5^(CF_3_)-carbon (pyrimidine ring) at 107.5 ppm.

#### 2.1.2. 2-[2-(4-Nitro-phenylamino)-5-trifluoromethyl-pyrimidin-4-ylamino]-thiophene-3-carboxylic acid methylamide (2)

To a solution of compound **1** (6 mmol) in TFE (2,2,2-trifluoroethanol, 24 mL) was added 4-nitroaniline (6.6 mmol) and TFA (trifluoroacetic acid, 18 mmol). The resulted mixture was heated to reflux under nitrogen atmosphere and stirred overnight before cooled to room temperature. The mixture was added EtOAc (100 mL) and washed with saturated NaHCO_3_ (3 × 50 mL). The organic layer was dried over magnesium sulphate, filtered, and concentrated *in vacuo* to afford the crude compound. The residue was purified by silica-gel column using DCM/MeOH = 30/1 to give the product as yellow solid (1.13 g; 43% yield). mp >250 °C; ^1^H NMR (400 MHz, DMSO-d_6_) δ 13.13 (s, 1H), 10.40 (s, 1H), 8.62 (s, 1H), 8.47 (d, *J* = 4.4 Hz, 1H), 8.24 (d, *J* = 9.2 Hz, 2H), 8.05 (d, *J* = 9.2 Hz, 2H), 7.48 (d, *J* = 6.0 Hz, 1H), 7.13 (d, *J* = 6.0 Hz, 1H), 2.81 (d, *J* = 4.4 Hz, 3H); ^13 ^C NMR (100 MHz, DMSO-d_6_) δ 166.2, 160.4, 156.1, 153.2, 146.3, 145.6, 142.0, 125.1, 123.0, 120.3, 117.7, 115.7, 101.2, 100.9, 26.2; ESI-HRMS C_17_H_13_F_3_N_6_O_3_S ([M + Na]^+^): calcd 461.0620, found 461.0606.

#### 2.1.3. 2-[2-(4-Amino-phenylamino)-5-trifluoromethyl-pyrimidin-4-ylamino]-thiophene-3-carboxylic acid methylamide (3)

To a solution of compound **2** (876 mg, 2 mmol) in methanol (20 mL) was added Pd/C (263 mg). The mixture was stirred at room temperature under hydrogen atmosphere for 24 h. The solution was filtered with celite and the filtration was evaporated under vacuum. The crude solid was recrystallized with methanol to afford compound **3** as yellow solid (0.286g; 35% yield). mp >250 °C; ^1^H NMR (400 MHz, DMSO-d_6_) δ 12.82 (s, 1H), 9.38 (s, 1H), 8.37 (s, 2H), 7.58 − 6.88 (m, 4H), 6.57 (d, *J* = 7.6 Hz, 2H), 4.97 (s, 2H), 2.80 (s, 3H); ^13 ^C NMR (100 MHz, DMSO-d_6_) δ 166.2, 156.3, 153.2, 146.2, 145.9, 138.4, 134.4, 130.0, 126.5, 123.8, 122.8, 117.2, 114.9, 114.8, 26.2; ESI-HRMS C_17_H_15_F_3_N_6_OS ([M + H]^+^): calcd 409.1052, found 409.1045.

#### 2.1.4. Synthesis of 4a–4f

A mixture of compound **3** (408 mg, 1 mmol) and DMF (8 mL) and DIEA (258 mg, 2 mmol) was stirred at room temperature. And then, the corresponding acid (1 mmol) and HATU (570 mg, 1.5 mmol) were added to the solution. The mixture was stirred at room temperature for 24 h. The solution was extracted with EtOAc (100 mL × 3), and the combined organic phase was washed with saturated brine (50 mL × 3). The organic layer was dried over magnesium sulphate, filtered, and concentrated *in vacuo* to afford the crude compound. The residue was purified by silica-gel column using DCM/MeOH = 30/1 to give the product **4a–4f**.

##### 2.1.4.1. 2-{2-[4-(4-Methoxy-benzoylamino)-phenylamino]-5-trifluoromethyl-pyrimidin-4-ylamino}-thiophene-3-carboxylic acid methylamide (4a)

White solid, 45% yield; mp >250 °C; ^1^H NMR (400 MHz, DMSO-d_6_) δ 12.93 (s, 1H), 10.09 (s, 1H), 9.75 (s, 1H), 8.48 (s, 1H), 8.44 − 8.38 (m, 1H), 7.98 (d, *J* = 8.8 Hz, 2H), 7.67 (m, 4H), 7.43 (d, *J* = 6.0 Hz, 1H), 7.07 (d, *J* = 8.8 Hz, 2H), 3.85 (s, 3H), 2.80 (d, *J* = 4.4 Hz, 3H); ^13 ^C NMR (125 MHz, DMSO-d_6_) δ 153.0, 152.1, 149.9, 145.1, 142.6, 138.6, 136.9, 135.6, 127.8, 125.5, 124.0, 122.0, 120.9, 118.3, 116.9, 113.9, 112.2, 111.5, 111.3, 64.8, 41.0; ESI-HRMS C_25_H_21_F_3_N_6_O_3_S ([M + H]^+^): calcd 543.1420, found 543.1426; Anal. calcd for C_25_H_21_F_3_N_6_O_3_S: C 55.35, H 3.90, N 15.49; found C 55.31, H 3.87, N 15.51.

##### 2.1.4.2. 2-{2-[4-(2-Fluoro-benzoylamino)-phenylamino]-5-trifluoromethyl-pyrimidin-4-ylamino}-thiophene-3-carboxylic acid methylamide (4b)

White solid, 44% yield; mp: 224.8-226.3 °C; ^1^H NMR (400 MHz, DMSO-d_6_) δ 12.94 (s, 1H), 10.39 (s, 1H), 9.78 (s, 1H), 8.48 (s, 1H), 8.41 (q, *J* = 4.0 Hz, 1H), 7.65 (m, 6H), 7.38 (m, 3H), 7.01 (s, 1H), 2.81 (d, *J* = 4.4 Hz, 3H); ^13 ^C NMR (125 MHz, DMSO-d_6_) δ 153.0, 150.4, 148.3, 146.7, 145.1, 142.6, 136.9, 128.2, 126.4, 124.4, 124.3, 120.9, 120.4, 120.1, 120.1, 119.1, 118.3, 116.4, 113.9, 113.4, 113.3, 112.2, 41.0; ESI-HRMS C_24_H_18_F_4_N_6_O_2_S ([M + H]^+^): calcd 531.1220, found 531.1224; Anal. calcd for C_24_H_18_F_4_N_6_O_2_S: C 54.34, H 3.42, N 15.84; found C 54.32, H 3.41, N 15.88.

##### 2.1.4.3. 2-{2-[4–(3-Fluoro-benzoylamino)-phenylamino]-5-trifluoromethyl-pyrimidin-4-ylamino}-thiophene-3-carboxylic acid methylamide (4c)

White solid, 41% yield; mp: 217.1-219.0 °C; ^1^H NMR (400 MHz, DMSO-d_6_) δ 12.93 (s, 1H), 10.32 (s, 1H), 9.78 (s, 1H), 8.48 (s, 1H), 8.41 (q, *J* = 4.0 Hz, 1H), 7.86 − 7.72 (m, 4H), 7.66 − 7.57 (m, 3H), 7.45 (dd, *J* = 6.4, 4.4 Hz, 2H), 7.01 (s, 1H), 2.80 (d, *J* = 4.4 Hz, 3H); ^13 ^C NMR (125 MHz, DMSO-d_6_) δ 153.0, 151.5, 150.8, 149.2, 145.1, 142.6, 136.8, 130.3, 124.9, 124.9, 120.9, 119.5, 119.2, 118.3, 117.0, 115.2, 115.1, 113.9, 112.2, 112.1, 111.9, 41.0; ESI-HRMS C_24_H_18_F_4_N_6_O_2_S ([M + H]^+^): calcd 531.1220, found 531.1226; Anal. calcd for C_24_H_18_F_4_N_6_O_2_S: C 54.34, H 3.42, N 15.84; found C 54.29, H 3.44, N 15.87.

##### 2.1.4.4. 2-{2-[4-(4-Fluoro-benzoylamino)-phenylamino]-5-trifluoromethyl-pyrimidin-4-ylamino}-thiophene-3-carboxylic acid methylamide (4d)

White solid, 46% yield; mp: 233.5–235.3 °C; ^1^H NMR (400 MHz, DMSO-d_6_) δ 12.93 (s, 1H), 10.26 (s, 1H), 9.78 (s, 1H), 8.48 (s, 1H), 8.41 (q, *J* = 4.0 Hz, 1H), 8.06 (dd, *J* = 8.8, 5.6 Hz, 2H), 7.70 (m, 4H), 7.47 − 7.34 (m, 3H), 7.01 (d, *J* = 2.4 Hz, 1H), 2.80 (d, *J* = 4.4 Hz, 3H); ^13 ^C NMR (125 MHz, DMSO-d_6_) δ 153.0, 152.4, 151.8, 150.9, 145.1, 145.1, 142.6, 136.8, 125.5, 124.7, 124.7, 120.9, 119.2, 118.3, 117.0, 113.9, 112.7, 112.6, 112.2, 41.0; ESI-HRMS C_24_H_18_F_4_N_6_O_2_S ([M + H]^+^): calcd 531.1220, found 531.1224; Anal. calcd for C_24_H_18_F_4_N_6_O_2_S: C 54.34, H 3.42, N 15.84; found C 54.38, H 3.47, N 15.77.

##### 2.1.4.5. 2-{2-[4-(3,4-Difluoro-benzoylamino)-phenylamino]-5-trifluoromethyl-pyrimidin-4-ylamino}-thiophene-3-carboxylic acid methylamide (4e)

White solid, 41% yield; mp: 244.3–245.9 °C; ^1^H NMR (400 MHz, DMSO-d_6_) δ 12.94 (s, 1H), 10.32 (s, 1H), 9.79 (s, 1H), 8.48 (s, 1H), 8.41 (q, *J* = 4.0 Hz, 1H), 8.09 − 7.87 (m, 2H), 7.75 − 7.60 (m, 5H), 7.44 (d, *J* = 6.0 Hz, 1H), 7.00 (s, 1H), 2.80 (d, *J* = 4.4 Hz, 3H); ^13 ^C NMR (125 MHz, DMSO-d_6_) δ 153.0, 150.8, 145.1, 142.6, 140.5, 140.5, 139.0, 138.9, 136.8, 128.3, 126.3, 120.6, 119.2, 118.3, 117.0, 114.9, 114.5, 114.2, 114.1, 113.9, 112.2, 41.0; ESI-HRMS C_24_H_17_F_5_N_6_O_2_S ([M + H]^+^): calcd 549.1126, found 549.1130; Anal. calcd for C_24_H_17_F_5_N_6_O_2_S: C 52.55, H 3.12, N 15.32; found C 52.53, H 3.10, N 15.38.

##### 2.1.4.6. 2-{5-Trifluoromethyl-2-[4-(3-trifluoromethyl-benzoylamino)-phenylamino]-pyrimidin-4-ylamino}-thiophene-3-carboxylic acid methylamide (4f)

White solid, 38% yield; mp >250 °C; ^1^H NMR (400 MHz, DMSO-d_6_) δ 12.94 (s, 1H), 10.48 (s, 1H), 9.80 (s, 1H), 8.49 (s, 1H), 8.41 (q, *J* = 4.0 Hz, 1H), 8.34 − 8.27 (m, 2H), 7.98 (d, *J* = 7.6 Hz, 1H), 7.76 (m, 5H), 7.44 (d, *J* = 6.0 Hz, 1H), 7.01 (d, *J* = 2.8 Hz, 1H), 2.81 (d, *J* = 4.4 Hz, 3H); ^13 ^C NMR (125 MHz, DMSO-d_6_) δ 153.0, 151.4, 145.1, 142.6, 136.8, 129.1, 128.3, 128.0, 125.9, 124.2, 123.9, 123.7, 122.9, 120.9, 120.5, 119.8, 119.2, 118.8, 118.3, 117.1, 113.9, 112.2, 41.0; ESI-HRMS C_25_H_18_F_6_N_6_O_2_S ([M + H]^+^): calcd 581.1188, found 581.1195; Anal. calcd for C_25_H_18_F_6_N_6_O_2_S: C 51.73, H 3.13, N 14.48; found C 51.71, H 3.12, N 14.52.

#### 2.1.5. 2-{2-[4-(2-Methoxy-3,4-dioxo-cyclobut-1-enylamino)-phenylamino]-5-trifluoromethyl-pyrimidin-4-ylamino}-thiophene-3-carboxylic acid methylamide (5)

To a solution of compound **3** (1.224 g, 3 mmol) in DMF (15 mL) was added dimethyl squarate (426 mg, 3 mmol) and DIEA (516 mg, 4 mmol). The mixture was stirred at room temperature for overnight. The mixture was extracted with EtOAc (150 mL × 2) and the combined organic phase was washed with saturated brine (100 mL × 3). The organic layer was dried over Na_2_SO_4_, filtered, and concentrated *in vacuo* to afford the crude compound. The residue was purified by silica-gel column using DCM/MeOH = 30/1 to give the product. White solid 886 mg; 57% yield; mp: 233.1–235.0 °C; ^1^H NMR (400 MHz, DMSO-d_6_) δ 12.94 (s, 1H), 10.77 (s, 1H), 9.79 (d, *J* = 0.8 Hz, 1H), 8.55 − 8.35 (m, 2H), 7.62 (s, 2H), 7.38 (m, 3H), 7.01 (s, 1H), 4.39 (s, 3H), 2.80 (d, *J* = 3.6 Hz, 3H); ^13 ^C NMR (125 MHz, DMSO-d_6_) δ 187.5, 170.5, 167.2, 155.6, 153.0, 150.3, 145.1, 142.6, 136.8, 132.0, 128.6, 120.8, 119.1, 118.3, 116.4, 114.0, 112.2, 68.8, 41.0; ESI-HRMS C_22_H_17_F_3_N_6_O_4_S ([M + H]^+^): calcd 519.1056, found 519.1061.

#### 2.1.6. Synthesis of 6a–6i

To a solution of compound **5** (518 mg, 1 mmol) in DMF (10 mL) was the corresponding aniline (1.2 mmol) and DIEA (129 mg, 1 mmol). The mixture was stirred at 80 °C for 12 h. The mixture was extracted with EtOAc (100 mL × 3) and the combined organic phase was washed with saturated brine (50 mL × 3). The organic layer was dried over magnesium sulphate, filtered, and concentrated *in vacuo* to afford the crude compound. The residue was purified by silica-gel column using DCM/MeOH = 30/1 to give the product **6a–6i**.

##### 2.1.6.1. 2-{2-[4-(3,4-Dioxo-2-propylamino-cyclobut-1-enylamino)-phenylamino]-5-trifluoromethyl-pyrimidin-4-ylamino}-thiophene-3-carboxylic acid methylamide (6a)

White solid, 48% yield; mp >250 °C; ^1^H NMR (400 MHz, DMSO-d_6_) δ 12.93 (s, 1H), 9.70 (m, 2H), 8.47 (s, 1H), 8.41 (d, *J* = 4.4 Hz, 1H), 7.62 (m, 2H), 7.41 (d, *J* = 9.2 Hz, 3H), 7.01 (d, *J* = 3.2 Hz, 1H), 3.58 (m, 2H), 2.80 (d, *J* = 4.4 Hz, 2H), 1.60 (m, 2H), 0.94 (t, *J* = 7.2 Hz, 3H); ^13 ^C NMR (125 MHz, DMSO-d_6_) δ 190.4, 170.0, 167.3, 164.6, 158.3, 155.7, 153.0, 151.1, 145.1, 142.6, 136.8, 128.3, 120.9, 118.3, 115.1, 114.0, 112.2, 56.7, 41.0, 39.6, 29.1; ESI-HRMS C_24_H_22_F_3_N_7_O_3_S ([M + H]^+^): calcd 546.1529, found 546.1533; Anal. calcd for C_24_H_22_F_3_N_7_O_3_S: C 52.84, H 4.06, N 17.97; found C 52.81, H 4.09, N 17.92.

##### 2.1.6.2. 2-{2-[4-(2-Hexylamino-3,4-dioxo-cyclobut-1-enylamino)-phenylamino]-5-trifluoromethyl-pyrimidin-4-ylamino}-thiophene-3-carboxylic acid methylamide (6b)

White solid, 52% yield; mp >250 °C; ^1^H NMR (400 MHz, DMSO-d_6_) δ 12.93 (s, 1H), 9.69 (m, 2H), 8.44 (m, 2H), 7.52 (m, 6H), 7.02 (s, 1H), 3.61 (s, 2H), 2.80 (s, 3H), 1.57 (s, 2H), 1.30 (s, 6H), 0.88 (s, 3H); ^13 ^C NMR (125 MHz, DMSO-d_6_) δ 190.7, 170.1, 167.4, 164.6, 155.7, 153.0, 151.2, 147.9, 145.1, 142.6, 136.8, 120.9, 118.3, 115.1, 114.0, 112.2, 55.4, 45.1, 44.9, 41.0, 40.9, 38.1, 31.6; ESI-HRMS C_27_H_28_F_3_N_7_O_3_S ([M + Na]^+^): calcd 610.1822, found 610.1823; Anal. calcd for C_27_H_28_F_3_N_7_O_3_S: C 55.19, H 4.80, N 16.69; found C 55.21, H 4.77, N 16.65.

##### 2.1.6.3. 2–(2-{4-[2-(2-Hydroxy-ethylamino)-3,4-dioxo-cyclobut-1-enylamino]-phenylamino}-5-trifluoromethyl-pyrimidin-4-ylamino)-thiophene-3-carboxylic acid methylamide (6c)

White solid, 39% yield; mp > 250 °C; ^1^H NMR (400 MHz, DMSO-d_6_) δ 12.93 (s, 1H), 9.77 (d, *J* = 11.6 Hz, 2H), 8.47 (s, 1H), 8.41 (q, *J* = 4.0 Hz, 1H), 7.83 (s, 1H), 7.51 (m, 5H), 7.02 (d, *J* = 3.6 Hz, 1H), 5.03 (s, 1H), 3.68 (d, *J* = 4.8 Hz, 2H), 3.60 (t, *J* = 5.2 Hz, 2H), 2.80 (d, *J* = 4.4 Hz, 3H); ^13 ^C NMR (125 MHz, DMSO-d_6_) δ 190.5, 170.2, 167.4, 164.7, 155.8, 153.0, 151.2, 150.3, 149.4, 145.1, 142.6, 128.4, 119.2, 118.3, 115.0, 114.0, 112.2, 68.2, 49.0, 41.0; ESI-HRMS C_23_H_20_F_3_N_7_O_4_S ([M + H]^+^): calcd 548.1322, found 548.1326; Anal. calcd for C_23_H_20_F_3_N_7_O_4_S: C 50.46, H 3.68, N 17.91; found C 50.44, H 3.71, N 17.88.

##### 2.1.6.4. 2-{2-[4-(2-Isopropylamino-3,4-dioxo-cyclobut-1-enylamino)-phenylamino]-5-trifluoromethyl-pyrimidin-4-ylamino}-thiophene-3-carboxylic acid methylamide (6d)

White solid, 45% yield; mp >250 °C; ^1^H NMR (400 MHz, DMSO-d_6_) δ 12.93 (s, 1H), 9.76 (s, 1H), 9.56 (s, 1H), 8.47 (s, 1H), 8.41 (d, *J* = 4.4 Hz, 1H), 7.62 (d, *J* = 12.8 Hz, 3H), 7.43 (t, *J* = 6.4 Hz, 3H), 7.01 (d, *J* = 3.2 Hz, 1H), 4.21 (dd, *J* = 14.0, 6.8 Hz, 1H), 2.80 (d, *J* = 4.4 Hz, 3H), 1.27 (d, *J* = 6.4 Hz, 6H); ^13 ^C NMR (125 MHz, DMSO-d_6_) δ 190.3, 169.9, 167.2, 164.5, 155.1, 153.0, 151.2, 150.3, 146.1, 145.1, 142.6, 136.8, 119.2, 118.3, 115.0, 114.0, 112.2, 49.0, 41.0, 39.4; ESI-HRMS C_24_H_22_F_3_N_7_O_3_S ([M + H]^+^): calcd 546.1529, found 546.1533; Anal. calcd for C_24_H_22_F_3_N_7_O_3_S: C 52.84, H 4.06, N 17.97; found C 52.81, H 4.09, N 18.02.

##### 2.1.6.5. 2-{2-[4-(2-Cyclopentylamino-3,4-dioxo-cyclobut-1-enylamino)-phenylamino]-5-trifluoromethyl-pyrimidin-4-ylamino}-thiophene-3-carboxylic acid methylamide (6e)

White solid, 41% yield; mp > 250 °C; ^1^H NMR (400 MHz, DMSO-d_6_) δ 12.93 (s, 1H), 9.76 (s, 1H), 9.54 (s, 1H), 8.51 − 8.35 (m, 2H), 7.66 (m, 3H), 7.42 (d, *J* = 7.6 Hz, 3H), 7.01 (s, 1H), 4.41 (d, *J* = 6.4 Hz, 1H), 2.80 (d, *J* = 4.0 Hz, 3H), 1.98 (dd, *J* = 10.8, 4.4 Hz, 2H), 1.72 (d, *J* = 5.6 Hz, 2H), 1.67 − 1.53 (m, 4H); ^13 ^C NMR (125 MHz, DMSO-d_6_) δ 191.0, 170.1, 167.2, 164.5, 159.0, 155.2, 153.0, 151.2, 150.3, 145.1, 142.6, 136.8, 133.7, 118.3, 115.0, 114.0, 112.2, 64.8, 45.1, 41.0, 38.9; ESI-HRMS C_26_H_24_F_3_N_7_O_3_S ([M + H]^+^): calcd 572.1686, found 572.1690; Anal. calcd for C_26_H_24_F_3_N_7_O_3_S: C 54.63, H 4.23, N 17.15; found C 54.66, H 4.22, N 17.17.

##### 2.1.6.6. 2-{2-[4-(2-Cyclohexylamino-3,4-dioxo-cyclobut-1-enylamino)-phenylamino]-5-trifluoromethyl-pyrimidin-4-ylamino}-thiophene-3-carboxylic acid methylamide (6f)

White solid, 42% yield; mp > 250 °C; ^1^H NMR (400 MHz, DMSO-d_6_) δ 12.93 (s, 1H), 9.76 (s, 1H), 9.58 (s, 1H), 8.47 (s, 1H), 8.41 (d, *J* = 4.4 Hz, 1H), 7.65 (m, 3H), 7.42 (d, *J* = 8.0 Hz, 3H), 7.01 (d, *J* = 2.0 Hz, 1H), 3.88 (s, 1H), 2.80 (d, *J* = 4.4 Hz, 3H), 1.95 (s, 2H), 1.81 − 1.52 (m, 4H), 1.35 (t, *J* = 9.2 Hz, 4H); ^13 ^C NMR (125 MHz, DMSO-d_6_) δ 190.4, 170.1, 167.1, 164.5, 155.0, 153.0, 151.2, 150.3, 145.1, 142.6, 136.8, 127.5, 119.2, 118.3, 115.0, 114.0, 112.2, 62.4, 45.0, 41.0, 40.2, 39.6; ESI-HRMS C_27_H_26_F_3_N_7_O_3_S ([M + H]^+^): calcd 586.1842, found 586.1847; Anal. calcd for C_27_H_26_F_3_N_7_O_3_S: C 55.38, H 4.48, N 16.74; found C 55.34, H 4.51, N 16.77.

##### 2.1.6.7. 2-(2-{4-[2–(4-Hydroxy-cyclohexylamino)-3,4-dioxo-cyclobut-1-enylamino]-phenylamino}-5-trifluoromethyl-pyrimidin-4-ylamino)-thiophene-3-carboxylic acid methylamide (6g)

White solid, 38% yield; mp > 250 °C; ^1^H NMR (400 MHz, DMSO-d_6_) δ 12.92 (s, 1H), 10.94 (s, 1H), 9.73 (s, 1H), 9.38 (s, 2H), 8.90 (s, 1H), 7.50 (d, *J* = 18.0 Hz, 5H), 7.00 (s, 1H), 4.61 (d, *J* = 3.6 Hz, 1H), 3.85 (s, 1H), 3.11 (d, *J* = 6.4 Hz, 1H), 2.80 (d, *J* = 4.0 Hz, 3H), 2.04 − 1.85 (m, 4H), 1.27 (d, *J* = 6.4 Hz, 4H); ^13 ^C NMR (125 MHz, DMSO-d_6_) δ 190.1, 170.8, 166.9, 164.3, 157.4, 155.3, 153.0, 151.4, 150.3, 145.1, 143.0, 127.0, 119.6, 118.4, 114.8, 114.0, 112.2, 63.1, 53.7, 34.8, 33.8, 30.2; ESI-HRMS C_27_H_26_F_3_N_7_O_4_S ([M + Na]^+^): calcd 624.1611, found 624.1614; Anal. calcd for C_27_H_26_F_3_N_7_O_4_S: C 53.90, H 4.36, N 16.30; found C 53.91, H 4.32, N 16.35.

##### 2.1.6.8. 2-(2-{4-[2-(1-Methyl-piperidin-4-ylamino)-3,4-dioxo-cyclobut-1-enylamino]-phenylamino}-5-trifluoromethyl-pyrimidin-4-ylamino)-thiophene-3-carboxylic acid methylamide (6h)

White solid, 35% yield; mp > 250 °C; ^1^H NMR (400 MHz, DMSO-d_6_) δ 12.93 (s, 1H), 9.77 (s, 2H), 8.44 (m, 2H), 7.89 (s, 1H), 7.60 (s, 2H), 7.44 (s, 3H), 7.01 (s, 1H), 3.90 (s, 1H), 2.97 − 2.70 (m, 6H), 2.23 (m, 4H), 1.97 (d, *J* = 9.2 Hz, 2H), 1.61 (d, *J* = 10.8 Hz, 2H); ^13 ^C NMR (125 MHz, DMSO-d_6_) δ 190.8, 170.4, 167.0, 164.6, 159.0, 155.1, 153.0, 151.4, 145.1, 142.6, 136.8, 128.3, 119.2, 118.3, 115.0, 114.0, 112.2, 63.0, 60.5, 56.7, 46.4, 41.0; ESI-HRMS C_27_H_27_F_3_N_8_O_3_S ([M + H]^+^): calcd 601.1951, found 601.1954; Anal. calcd for C_27_H_27_F_3_N_8_O_3_S: C 53.99, H 4.53, N 18.66; found C 54.01, H 4.52, N 18.68.

##### 2.1.6.9. [2-(2-{4-[4-(3-Methylcarbamoyl-thiophen-2-ylamino)-5-trifluoromethyl-pyrimidin-2-ylamino]-phenylamino}-3,4-dioxo-cyclobut-1-enylamino)-ethyl]-carbamic acid tert-butyl ester (6i)

White solid, 43% yield; mp > 250 °C; ^1^H NMR (400 MHz, DMSO-d_6_) δ 12.93 (s, 1H), 9.72 (m, 2H), 8.47 (s, 1H), 8.41 (d, *J* = 4.4 Hz, 1H), 7.60 (s, 3H), 7.42 (dd, *J* = 13.2, 7.2 Hz, 3H), 6.99 (s, 2H), 3.62 (s, 2H), 3.21 − 3.14 (m, 2H), 2.80 (d, *J* = 4.4 Hz, 3H), 1.37 (s, 9H); ^13 ^C NMR (125 MHz, DMSO-d_6_) δ 190.9, 170.8, 167.3, 164.9, 156.0, 153.0, 151.3, 149.4, 145.1, 142.7, 136.8, 127.4, 120.9, 118.3, 115.0, 114.0, 112.2, 82.7, 55.5, 53.3, 43.0, 41.0; ESI-HRMS C_28_H_29_F_3_N_8_O_5_S ([M + H]^+^): calcd 647.2006, found 647.2009; Anal. calcd for C_28_H_29_F_3_N_8_O_5_S: C 52.01, H 4.52, N 17.33; found C 52.03, H 4.51, N 17.31.

#### 2.1.7. 2-(2-{4-[2-(2-Amino-ethylamino)-3,4-dioxo-cyclobut-1-enylamino]-phenylamino}-5-trifluoromethyl-pyrimidin-4-ylamino)-thiophene-3-carboxylic acid methylamide (7)

A mixture of compound **6i** (646 mg, 1 mmol) and hydrogen chloride-ethyl acetate solution (3 mL, 1 mol/L) was stirred at room temperature for overnight. The mixture was evaporated under vacuum and extracted with EtOAc (50 mL × 2) and the combined organic phase was washed with saturated brine (20 mL × 3). The organic layer was dried over magnesium sulphate, filtered, and concentrated *in vacuo* to afford the crude compound. The residue was recrystallized with methanol to afford compound **7**. Yellow solid 431 mg; 79% yield; mp: 220.2–222.5 °C; ^1^H NMR (400 MHz, DMSO-d_6_) δ 12.93 (s, 1H), 10.19 (s, 1H), 9.77 (d, *J* = 3.2 Hz, 1H), 8.50 − 8.40 (m, 2H), 8.22 (s, 1H), 7.99 (s, 2H), 7.60 (d, *J* = 4.0 Hz, 2H), 7.49 − 7.38 (m, 3H), 6.99 (d, *J* = 4.0 Hz, 1H), 3.82 (m, 2H), 3.09 (m, 2H), 2.80 (d, *J* = 4.4 Hz, 3H); ^13 ^C NMR (100 MHz, DMSO-d_6_) δ 181.2, 169.8, 166.2, 164.8, 159.3, 159.0, 158.6, 156.2, 153.2, 145.9, 130.9, 122.8, 119.0, 118.5, 117.4, 115.5, 115.2, 41.7, 38.9, 26.2; ESI-HRMS C_23_H_21_F_3_N_8_O_3_S ([M + H]^+^): calcd 547.1482, found 547.1489.

#### 2.1.8. Synthesis of 8a–8g

A mixture of compound **7** (546 mg, 1 mmol) and DMF (10 mL) and DIEA (258 mg, 2 mmol) was stirred at room temperature. And then the corresponding acid (1 mmol) and HATU (760 mg, 2 mmol) were added into the solution. The mixture was stirred at room temperature for overnight. The solution was extracted with EtOAc (100 mL × 3) and the combined organic phase was washed with saturated brine (50 mL × 3). The organic layer was dried over Na_2_SO_4_ and concentrated *in vacuo* to afford the crude compound. The residue was purified by silica-gel column using DCM/MeOH = 30/1 to give the product **8a–8g**.

##### 2.1.8.1. 2-(2-{4-[3,4-Dioxo-2-(2-propionylamino-ethylamino)-cyclobut-1-enylamino]-phenylamino}-5-trifluoromethyl-pyrimidin-4-ylamino)-thiophene-3-carboxylic acid methylamide (8a)

White solid, 58% yield; mp > 250 °C; ^1^H NMR (400 MHz, DMSO-d_6_) δ 12.92 (s, 1H), 10.83 (s, 1H), 9.75 (s, 1H), 8.72 (s, 1H), 8.45 (d, *J* = 5.2 Hz, 4H), 7.52 (m, 4H), 6.99 (s, 1H), 3.63 (d, *J* = 5.2 Hz, 2H), 3.17 (s, 2H), 2.79 (d, *J* = 4.4 Hz, 3H), 2.14 − 2.04 (m, 2H), 1.03 − 0.94 (m, 3H); ^13 ^C NMR (100 MHz, DMSO-d_6_) δ 180.3, 173.8, 172.6, 170.0, 162.8, 156.3, 145.0, 139.7, 134.9, 128.0, 124.9, 123.7, 119.6, 118.5, 117.4, 117.1, 114.6, 114.2, 43.8, 39.2, 29.0, 26.2, 10.3; ESI-HRMS C_26_H_25_F_3_N_8_O_4_S ([M + H]^+^): calcd 603.1744, found 603.1750; Anal. calcd for C_26_H_25_F_3_N_8_O_4_S: C 51.82, H 4.18, N 18.60; found C 51.83, H 4.21, N 18.57.

##### 2.1.8.2. 2-(2-{4-[2-(2-Isobutyrylamino-ethylamino)-3,4-dioxo-cyclobut-1-enylamino]-phenylamino}-5-trifluoromethyl-pyrimidin-4-ylamino)-thiophene-3-carboxylic acid methylamide (8b)

White solid, 61% yield; mp > 250 °C; ^1^H NMR (400 MHz, DMSO-d_6_) δ 12.92 (s, 1H), 9.88 − 9.69 (m, 2H), 8.51 − 8.37 (m, 2H), 7.93 (s, 1H), 7.61 (d, *J* = 5.6 Hz, 3H), 7.42 (m, 3H), 7.00 (s, 1H), 3.64 (d, *J* = 3.6 Hz, 2H), 3.28 (d, *J* = 5.6 Hz, 2H), 2.80 (d, *J* = 4.4 Hz, 3H), 2.34 (m, 1H), 0.99 (d, *J* = 6.8 Hz, 6H); ^13 ^C NMR (100 MHz, DMSO-d_6_) δ 186.7, 176.9, 170.1, 166.2, 164.2, 161.0, 156.3, 153.2, 149.0, 147.3, 145.9, 134.4, 132.1, 126.3, 122.9, 118.5, 117.4, 115.2, 43.9, 39.1, 34.5, 26.2, 20.0; ESI-HRMS C_27_H_27_F_3_N_8_O_4_S ([M + H]^+^): calcd 617.1900, found 617.1908; Anal. calcd for C_27_H_27_F_3_N_8_O_4_S: C 52.59, H 4.41, N 18.17; found C 52.61, H 4.39, N 18.14.

##### 2.1.8.3. 2-[2–(4-{2-[2-(Cyclopentanecarbonyl-amino)-ethylamino]-3,4-dioxo-cyclobut-1-enylamino}-phenylamino)-5-trifluoromethyl-pyrimidin-4-ylamino]-thiophene-3-carboxylic acid methylamide (8c)

White solid, 62% yield; mp > 250 °C; ^1^H NMR (400 MHz, DMSO-d_6_) δ 12.92 (s, 1H), 10.79 (d, *J* = 2.4 Hz, 1H), 9.74 (s, 1H), 8.67 (s, 1H), 8.51 − 8.39 (m, 3H), 7.95 (s, 2H), 7.65 − 7.42 (m, 3H), 7.00 (s, 1H), 3.69 − 3.59 (m, 2H), 3.29 (d, *J* = 5.6 Hz, 2H), 2.79 (d, *J* = 4.0 Hz, 3H), 1.77 − 1.42 (m, 8H), 1.23 (s, 1H); ^13 ^C NMR (100 MHz, DMSO-d_6_) δ 187.9, 176.8, 172.7, 168.4, 166.3, 162.8, 147.2, 139.5, 136.9, 134.9, 133.8, 127.8, 125.0, 123.6, 119.3, 118.6, 117.4, 115.2, 44.8, 43.9, 36.3, 31.2, 30.4, 26.0; ESI-HRMS C_29_H_29_F_3_N_8_O_4_S ([M + H]^+^): calcd 643.2057, found 643.2062; Anal. calcd for C_29_H_29_F_3_N_8_O_4_S: C 54.20, H 4.55, N 17.44; found C 54.22, H 4.54, N 17.42.

##### 2.1.8.4. 2-[2-(4-{2-[2-(Cyclohexanecarbonyl-amino)-ethylamino]-3,4-dioxo-cyclobut-1-enylamino}-phenylamino)-5-trifluoromethyl-pyrimidin-4-ylamino]-thiophene-3-carboxylic acid methylamide (8d)

White solid, 66% yield; mp >250 °C; ^1^H NMR (400 MHz, DMSO-d_6_) δ 12.92 (s, 1H), 10.07 (s, 1H), 9.76 (s, 1H), 8.59 (d, *J* = 3.6 Hz, 4H), 8.40 (d, *J* = 8.4 Hz, 4H), 7.60 (s, 2H), 7.00 (s, 1H), 3.64 (d, *J* = 8.4 Hz, 3H), 3.13 (d, *J* = 6.8 Hz, 4H), 2.80 (d, *J* = 4.4 Hz, 4H), 1.66 (d, *J* = 10.8 Hz, 5H), 1.29 − 1.20 (m, 12H); ^13 ^C NMR (100 MHz, DMSO-d_6_) δ 184.8, 176.0, 170.4, 167.9, 166.2, 153.8, 148.5, 146.0, 142.6, 139.8, 134.9, 131.0, 128.2, 123.0, 119.9, 118.4, 117.4, 115.2, 44.5, 39.0, 31.1, 29.6, 26.2, 25.8, 24.4; ESI-HRMS C_30_H_31_F_3_N_8_O_4_S ([M + H]^+^): calcd 657.2213, found 657.2218; Anal. calcd for C_30_H_31_F_3_N_8_O_4_S: C 54.87, H 4.76, N 17.06; found C 54.89, H 4.75, N 17.05.

##### 2.1.8.5. 2-[2-(4-{3,4-Dioxo-2-[2-(3-phenyl-acryloylamino)-ethylamino]-cyclobut-1-enylamino}-phenylamino)-5-trifluoromethyl-pyrimidin-4-ylamino]-thiophene-3-carboxylic acid methylamide (8e)

White solid, 47% yield; mp > 250 °C; ^1^H NMR (400 MHz, DMSO-d_6_) δ 12.92 (s, 1H), 9.78 (s, 2H), 8.41 (m, 3H), 7.82 − 7.33 (m, 12H), 7.01 (d, *J* = 4.8 Hz, 1H), 6.64 (d, *J* = 15.6 Hz, 1H), 3.73 (d, *J* = 2.0 Hz, 2H), 3.45 (d, *J* = 3.2 Hz, 2H), 2.80 (d, *J* = 2.8 Hz, 3H); ^13 ^C NMR (100 MHz, DMSO-d_6_) δ 187.4, 181.7, 174.0, 169.8, 166.2, 162.9, 156.2, 149.5, 144.6, 140.1, 139.4, 137.0, 135.3, 134.4, 130.0, 129.4, 128.0, 123.8, 122.4, 121.1, 119.9, 119.4, 118.7, 114.1, 42.7, 38.4, 26.2; ESI-HRMS C_32_H_27_F_3_N_8_O_4_S ([M + H]^+^): calcd 677.1900, found 677.1909; Anal. calcd for C_32_H_27_F_3_N_8_O_4_S: C 56.80, H 4.02, N 16.56; found C 56.82, H 4.00, N 16.51.

##### 2.1.8.6. 2-{2-[4–(2-{2-[3-(3-Fluoro-phenyl)-acryloylamino]-ethylamino}-3,4-dioxo-cyclobut-1-enylamino)-phenylamino]-5-trifluoromethyl-pyrimidin-4-ylamino}-thiophene-3-carboxylic acid methylamide (8f)

White solid, 45% yield; mp > 250 °C; ^1^H NMR (400 MHz, DMSO-d_6_) δ 12.92 (s, 1H), 9.99 (s, 1H), 9.76 (s, 1H), 8.63 (d, *J* = 3.6 Hz, 3H), 8.44 (d, *J* = 8.8 Hz, 5H), 7.95 (s, 4H), 7.60 (d, *J* = 7.2 Hz, 3H), 7.00 (s, 1H), 3.73 (d, *J* = 10.4 Hz, 2H), 3.15 − 3.10 (m, 2H), 2.80 (d, *J* = 4.4 Hz, 3H); ^13 ^C NMR (100 MHz, DMSO-d_6_) δ 183.9, 176.9, 173.9, 166.2, 165.5, 164.3, 164.1, 161.7, 156.3, 153.2, 148.4, 145.9, 138.0, 131.4, 131.3, 126.3, 124.1, 122.8, 118.7, 117.4, 117.0, 116.7, 116.5, 115.2, 114.5, 114.3, 43.8, 39.0, 26.2; ESI-HRMS C_32_H_26_F_4_N_8_O_4_S ([M + H]^+^): calcd 695.1806, found 695.1807; Anal. calcd for C_32_H_26_F_4_N_8_O_4_S: C 55.33, H 3.77, N 16.13; found C 55.31, H 3.79, N 16.11.

##### 2.1.8.7. 2-{2-[4–(2-{2-[3–(4-Fluoro-phenyl)-acryloylamino]-ethylamino}-3,4-dioxo-cyclobut-1-enylamino)-phenylamino]-5-trifluoromethyl-pyrimidin-4-ylamino}-thiophene-3-carboxylic acid methylamide (8g)

White solid, 42% yield; mp >250 °C; ^1^H NMR (400 MHz, DMSO-d_6_) δ 12.92 (s, 1H), 11.03 (d, *J* = 5.6 Hz, 1H), 9.74 (s, 1H), 8.98 (s, 1H), 8.45 (m, 4H), 7.95 (s, 1H), 7.55 (m, 9H), 7.00 (s, 1H), 3.69 (d, *J* = 5.6 Hz, 2H), 3.16 − 3.02 (m, 2H), 2.79 (d, *J* = 4.4 Hz, 3H); ^13 ^C NMR (100 MHz, DMSO-d_6_) δ 188.5, 171.7, 165.7, 164.0, 162.8, 161.9, 155.7, 148.5, 146.9, 145.9, 143.3, 138.0, 134.9, 130.2, 130.1, 127.7, 124.0, 122.5, 119.3, 117.4, 116.5, 116.2, 115.2, 113.9, 43.7, 36.3, 26.2; ESI-HRMS C_32_H_26_F_4_N_8_O_4_S ([M + H]^+^): calcd 695.1806, found 695.1810; Anal. calcd for C_32_H_26_F_4_N_8_O_4_S: C 55.33, H 3.77, N 16.13; found C 55.28, H 3.80, N 16.12.

### 2.2. *In vitro* EGFR^wt^-TK assay

*In vitro* activities against wild type EGFR tyrosine kinase (EGFR^wt^) of compounds **4a–4f**, **6a–6i**, and **8a–8g** were tested with ELISA assay. The corresponding biochemical reagents were purchased from PTM Bio. Co., Ltd. The IC_50_ values of compounds **4b**, **4c**, **6e**, **6i**, and **8e–8g** were calculated from the dose − response curve, which was diluted to the corresponding concentration with kinase reaction buffer.

### 2.3. *In vitro* activity assay at cell level

#### 2.3.1. Cytotoxicity evaluation (MTT assay)

Three tumour cells with high expression of EGFR^wt^ were used to test the antitumor activity of the compounds including, A549 (Human non-small-cell lung cancer cell line) cells, PC-3 (Human prostate cancer cell line) cells, and HepG2 (Human hepatocellular carcinomas cell line) cells. They were all purchased from the Shanghai Cell Bank of the Chinese Academy of Sciences. L02 (normal human liver cell line) cells were also used to evaluate the cytotoxicity. *In vitro* cytotoxicity of compounds **4a–4f**, **6a–6i**, and **8a–8g** against three cancer cells and the normal cells were tested with MTT assay as our previous report^31^, and Gefitinib were used as positive controls. The IC_50_ values were calculated from the dose − response curve under Graph-Pad Prism.

#### 2.3.2. Cell apoptosis and cycle analysis

A549 cells were treated with different concentrations of compound **4c** under the kit’s instruction and then measured with Annexin V – FITC/PI apoptosis detection kit and Annexin V – FITC/PI cell cycle detection kit. The experimental data of apoptosis and cell cycle for A549 cells were evaluated in BD Accuri C6 flow cytometry (American BD Corporation Shanghai Co., Ltd.), provided by the School of Pharmaceutical Sciences, Guizhou University.

### 2.4. Molecular docking

The X-ray crystal structures of EGFR were obtained from the PDB bank (PDB entry 1M17 and PDB entry 6DUK), which defined the binding modes. The possible binding modes of compound **4c** were predicted with Sybyl X-2.0 software from Tripos Inc. USA.

### 2.5. Predicted ADMET studies

The absorption, distribution, metabolism, elimination, and toxicity (ADMET) parameters of compounds **4b**, **4c**, **6e**, **6i**, **8e–8g** and Gefitinib were calculated in CHARMM Force Field of Discovery Studio 2.5 Software (Accelrys, Inc., San Diego, USA).

## 3. Results and discussions

### 3.1. Chemistry

**Scheme 1.**
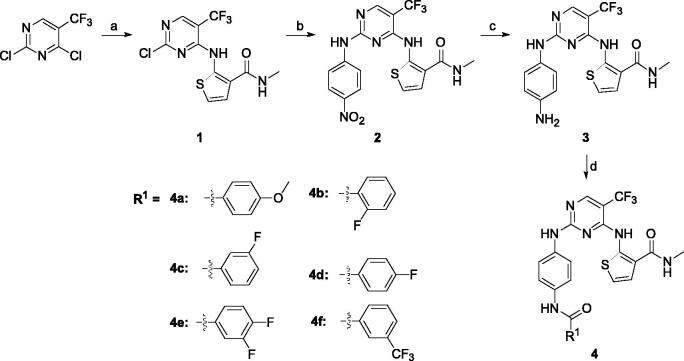
Synthetic route of target compounds **4a–4f**. Reagents and conditions: (a) 2-amino-*N*-methylthiophene-3-carboxamide, NaHCO_3_, EtOH, rt, overnight, 40% yield; (b) 4-nitroaniline, TFA, TFE, reflux, overnight, 43% yield; (c) Pd/C, MeOH, rt, 24 h, 35% yield; (d) corresponding acid, HATU, DIEA, DMF, rt, 12 h, 38%–46% yield.

The syntheses of compounds **4a–4f** were depicted in Scheme 1. Initially, 2,4-dichloro-5-trifluoromethyl-pyrimidine reacted with 2-amino-*N*-methylthiophene-3- carboxamide to produce compound **1** with 40% yield, which was confirmed by ^1^H-NMR, ^13 ^C-NMR, HRMS and HMBC (CF_3_ group interaction with N^4^-H in compound **1**). Subsequently, the 2-chloro group in the pyrimidine ring was substituted by 4-nitroaniline to give compound **2** with 43% yield. The nitro group of **2** was reduced to amino group at the presence of Pd/C, and compound **3** was obtained with 35% yield. At last, compound **3** reacted with different substituted carboxylic acids to obtain the compounds **4a–4f** in 38%−46% yield.

**Scheme 2.**
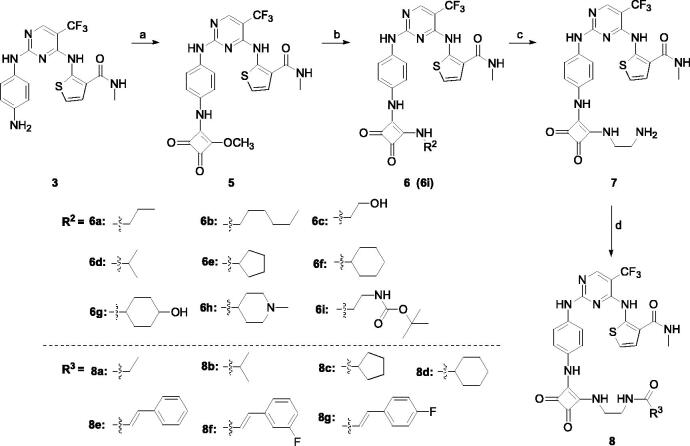
Synthetic route of target compounds **6** and **8**. Reagents and conditions: (a) dimethyl squarate, DIEA, DMF, rt, overnight, 57% yield; (b) corresponding amine, DIEA, DMF, 80 °C, 12 h, 38%–48% yield; (c) hydrogen chloride-ethyl acetate solution, 79% yield; (d) corresponding acid, HATU, DIEA, DMF, rt, overnight, 42%–66% yield.

Cyclobutene diketone was a kind of important fragment found in many kinase inhibitors^32^^,^^33^. Based on the widely biological activity of cyclobutene diketone, we introduced it into our target compounds. As shown in Scheme 2, compounds **6a–6i** and **8a–8g** were prepared. Compound **3** in Scheme 1 as the starting material successfully reacted with 3,4-dimethoxy-3- cyclobutene-1,2-dione to obtain intermediate **5** in 57% yield. Then, compound **5** reacted with different substituted amines, giving the compounds **6a–6i** in 35%−52% yields. After deprotected Boc group in compound **6i** under HCl, we got the other critical intermediate **7** with a high yield. Finally, the target compounds **8a–8g** were synthesised in the corresponding acid under the coupling reagent with moderate yield.

### 3.2. *In vitro* anti-tumour activity against cancer cell lines and kinases

Three tumour cell lines (A549, PC-3, and HepG2) were used to evaluate the antiproliferative activities of the final compounds with methyl thiazolyl tetrazolium colorimetric (MTT) assay^34^. The IC_50_ values are listed in Table 1. Gefitinib was performed as the positive control. The results suggested that some of the compounds exhibited well activities for all cancer cell lines. Against A549 cells, compounds **4b**, **4c**, **4f**, **6e**, **6i**, and **8c-8g** were more potent than Gefitinib (IC_50_ = 8.48 μM). Especially, the IC_50_ value of compound **4c** reached 0.56 μM for A549 cells. Against PC-3 cells, compounds **4a**–**4c**, **4f**, **6e**, **6i**, and **8c-8g** were more potent than Gefitinib (IC_50_ = 17.75 μM). Against HepG2 cells, compounds **4a**–**4c**, **4f**, **6d**, **6e**, **6i**, and **8c-8g** were more potent than Gefitinib (IC_50_ = 15.86 μM). In order to preliminarily estimate the activities of compounds against EGFR^wt^, the inhibition effects of target compounds at 1 μM were tested with ELISA assay. It can be discerned from the results in Table 1 that only compounds **4b**, **4c**, **6e**, **6i**, and **8e–8g** were more than 50% against EGFR^wt^. Hence, these seven compounds **4b**, **4c**, **6e**, **6i**, and **8e–8g** were selected for further studies to access corresponding IC_50_ values against EGFR^wt^.

**Table 1.** *In vitro* activities of target compounds for EGFR^wt^-TK and cancer cell lines^a^

Comp.	EGFR^wt^-TK inhibition rate (%, 1 μM)	IC_50_ (μM)^a^			
		A549	PC-3	HepG2	L02
**4a**	31.57 ± 3.27	9.32 ± 0.68	12.32 ± 0.89	11.46 ± 0.77	>40
**4b**	52.08 ± 1.93	4.33 ± 0.59	7.81 ± 0.53	7.62 ± 0.46	>40
**4c**	61.69 ± 2.08	0.56 ± 0.12	2.46 ± 0.42	2.21 ± 0.50	>40
**4d**	7.59 ± 0.25	>20	>20	>20	>40
**4e**	27.88 ± 1.34	>20	>20	>20	>40
**4f**	30.79 ± 1.09	6.14 ± 0.78	8.51 ± 0.64	8.36 ± 0.49	>40
**6a**	15.33 ± 0.48	>20	>20	>20	>40
**6b**	17.41 ± 0.32	>20	>20	>20	>40
**6c**	16.60 ± 0.21	>20	>20	>20	>40
**6d**	29.14 ± 1.03	10.13 ± 1.96	>20	14.09 ± 1.28	>40
**6e**	53.17 ± 3.05	7.14 ± 0.92	9.24 ± 0.86	9.00 ± 0.78	>40
**6f**	34.06 ± 1.29	18.11 ± 2.45	>20	>20	>40
**6g**	9.27 ± 0.25	>20	>20	>20	>40
**6h**	10.58 ± 0.93	>20	>20	>20	>40
**6i**	57.37 ± 1.82	3.11 ± 0.48	5.33 ± 0.47	5.24 ± 0.51	>40
**8a**	6.86 ± 0.11	>20	>20	>20	>40
**8b**	7.92 ± 0.54	>20	>20	>20	>40
**8c**	28.14 ± 1.27	7.32 ± 0.65	9.69 ± 1.13	9.54 ± 0.91	>40
**8d**	29.03 ± 1.55	6.14 ± 0.72	8.35 ± 0.74	8.28 ± 0.69	>40
**8e**	53.11 ± 1.75	3.15 ± 0.44	4.12 ± 0.65	4.31 ± 0.39	>40
**8f**	58.87 ± 1.92	2.17 ± 0.54	3.48 ± 0.22	3.09 ± 0.66	>40
**8g**	52.08 ± 1.93	3.14 ± 0.92	6.08 ± 0.94	5.33 ± 0.58	>40
**Gefitinib**	68.48 ± 0.79	8.48 ± 0.55	17.75 ± 1.38	15.86 ± 0.86	>40

^a^The values are mean ± SD of three replicates.

As shown in Table 2, the IC_50_ values of compound **4c** reached 0.32 μM against EGFR^wt^. The others were much higher than compound **4c**. Based on the results of EGFR^wt^, and three tumour cells, we found a similar structure-activity relationship (SAR) in both of them. The amide group (**4a–4f**) in the targets was more potent than the cyclobutene diketone derivatives (**6a–6i**). In addition, the extended chain compounds **8a–8g** also performed moderate antiproliferative activities. In general, compound **4c** was the best in all the target compounds, which was potentially developing into an anti-tumour reagent as the reports in literature^35–38^.

**Table 2.** IC_50_ values for EGFR^wt^

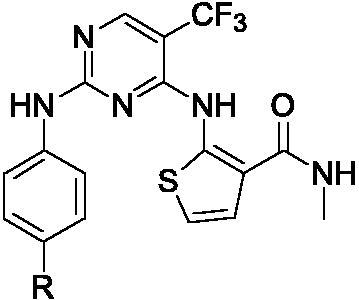			
Entry	Comp.	R	EGFR^wt^-TK IC_50_ (μM)
1	**4b**	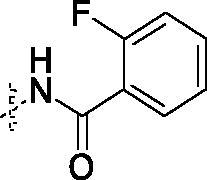	0.89 ± 0.12
2	**4c**	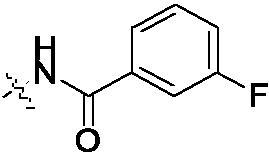	0.32 ± 0.047
3	**6e**	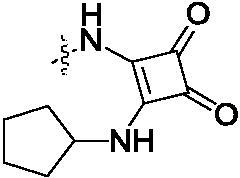	0.74 ± 0.10
4	**6i**	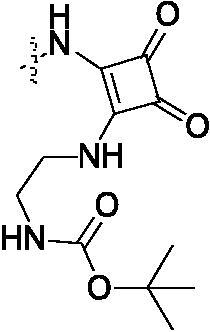	0.66 ± 0.13
5	**8e**	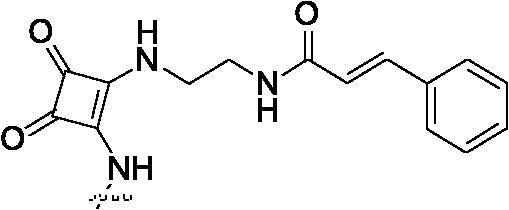	0.64 ± 0.10
6	**8f**	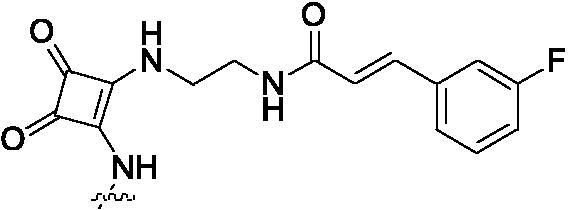	0.57 ± 0.29
7	**8g**	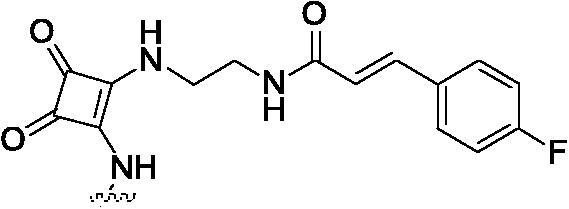	0.59 ± 0.15
8	Gefitinib		0.0061 ± 0.0003

^a^The values are mean ± SD of three replicates.

### 3.4. Effects of compound 4c on cell apoptosis of A549 cell line

**Figure 3.**
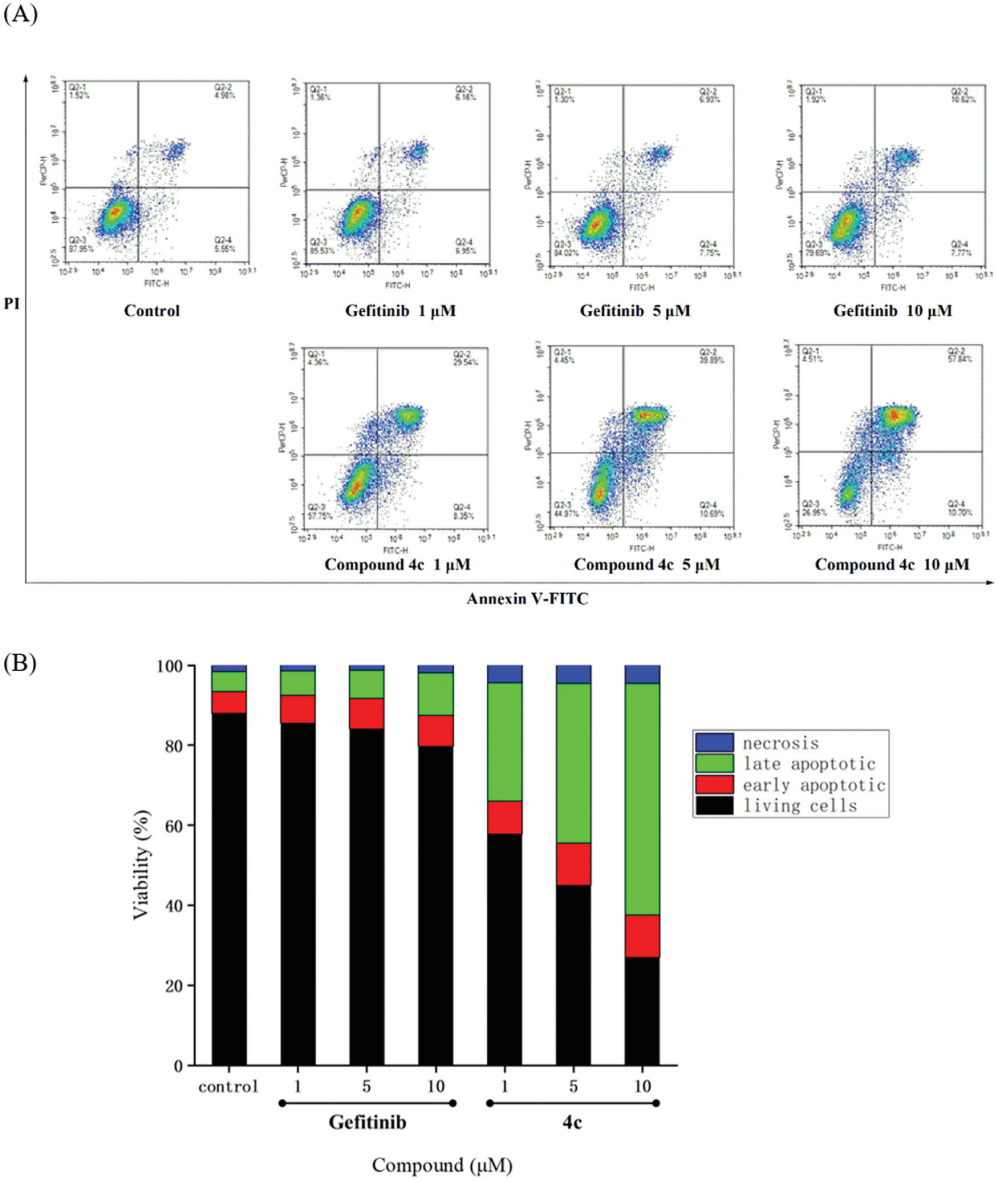
Compound **4c** induced A549 cell apoptosis in Annexin V-FITC assay. (A) Density plots were obtained by flow cytometry in the presence of different concentrations (1 μM, 5 μM and 10 μM); Gefitinib was used as the positive control. (B) Total apoptotic cells (%) at various concentrations of **4c** and Gefitinib.

Apoptosis of A549 cells is an efficient route to clear out the extra cancer cells in the tissue homeostasis. It has been found that many drugs for EGFR could induce apoptosis of A549 cells^39^. Therefore, compound **4c** was employed to investigate apoptosis against A549 cells. As shown in Figure 3(A), flow cytometry analysis of A549 treated with **4c** and Gefitinib at 1 µM, 5 µM, and 10 µM for 48 h demonstrated a notable increase in apoptotic cells with a dose-dependent fashion. Compared compound **4c** with Gefitinib at the same concentration (Figure 3(B)), the results showed that compound **4c** could better induce A549 cell apoptosis. Surprisingly, the ratio of apoptotic cells for compound **4c** reached 10.70% (early) and 57.84% (late) at 10 µM, which was much higher than the ratio of Gefitinib (7.77% and 10.62%).

### 3.5. Effects of compound 4c on cell cycle of A549 cell line

**Figure 4.**
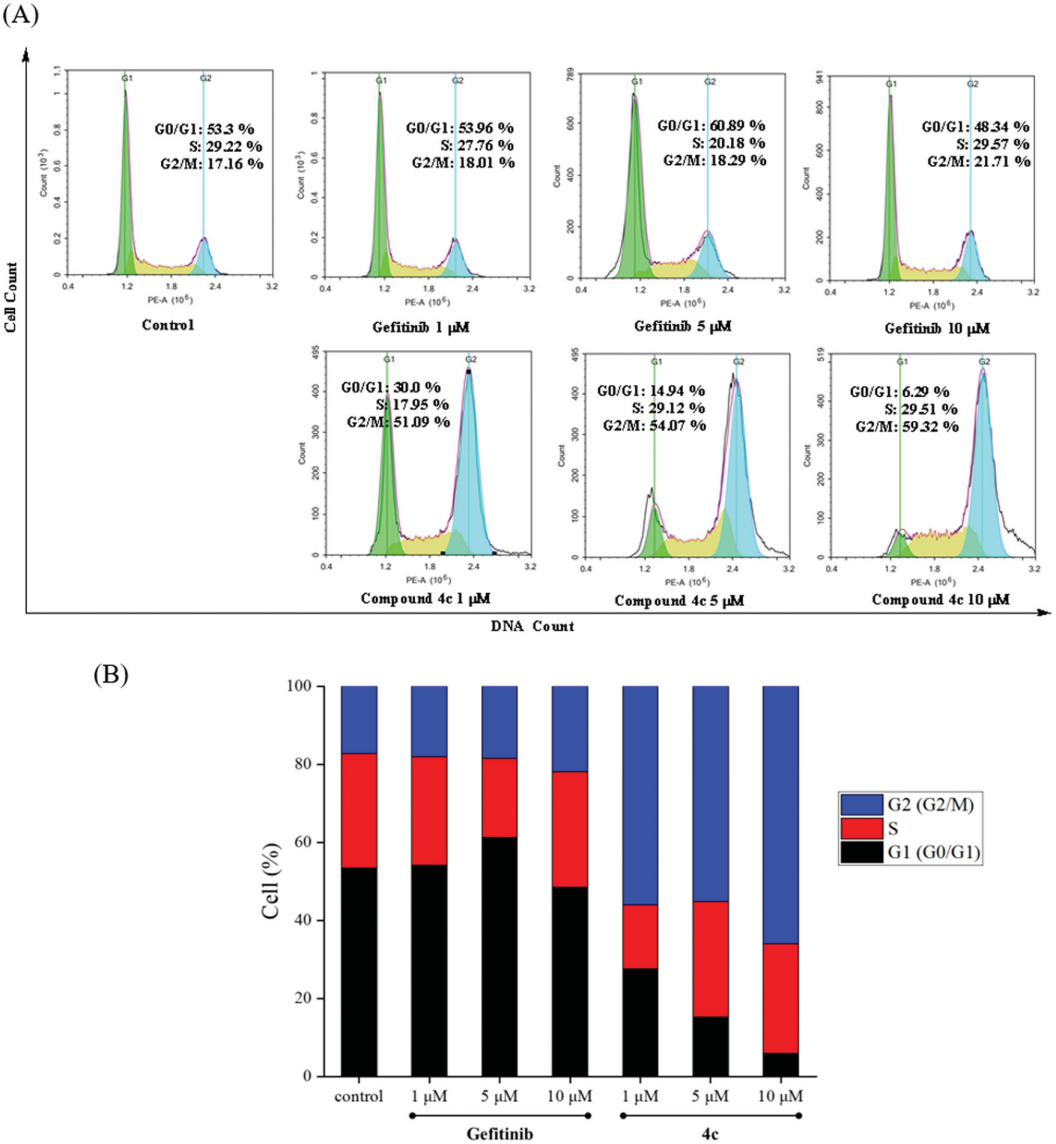
Cell cycle distribution of compound **4c** and Gefitinib against A549 was studied by flow cytometry. (A) A549 cells were cultured in the presence of different concentrations of **4c** (1 μM, 5 μM and 10 μM) or Gefitinib (1 μM, 5 μM and 10 μM) for 48 h, harvested, fixed, and labelled with PI, then analysed by FACS. Percentage of cells in G0/G1, S and G2/M phases are indicated. (B) Profiles obtained by FACS. The percentages for different phases of the cell cycle were illustrated in the histogram.

In order to investigate the effects of compound **4c** on the A549 cell cycle, flow cytometry was employed. As shown in Figure 4, A549 cell lines were treated with compound **4c** at 1 µM, 5 µM and 10 µM. For Gefitinib, the G2/M phase cells were slowly increased from 17.16% to 21.71%. Surprisingly, the G2/M phase cells of compound **4c** sharply increased from 17.16% to 59.32%, which reached 51.09% at 1 µM concentration. These data indicated that compound **4c** could arrest A549 cells in the G2/M phase.

### 3.6. Molecular docking studies

**Figure 5.**
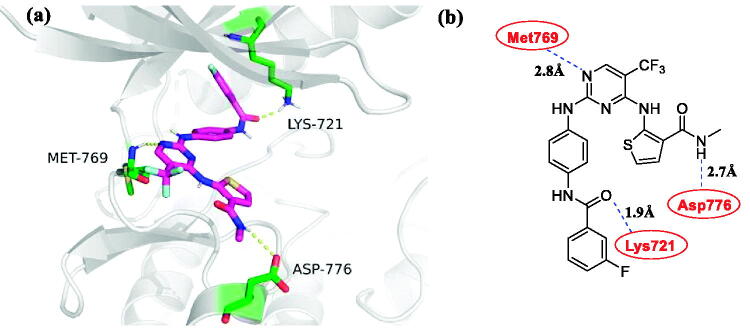
Docking structures of compound **4c**. (a) Binding configuration of compounds **4c** with EGFR^wt^ (PDB: 1M17); (b) The 2 D model of compound **4c** bound to EGFR^wt^ (PDB: 1M17).

Aiming to explain the activities of compound **4c** against EGFR, the possible binding modes were investigated in molecular docking through Sybyl X-2.0 software. The crystal structures of EGFR (PDB entry 1M17)^40^ were used for identifying candidate binding modes. As shown in Figure 5(a and b), compound **4c** formed hydrogen bonds with multiple amino acids, including Met769 (hydrogen bond length 2.8 Å), Asp776 (hydrogen bond length 2.7 Å), and Lys721 (hydrogen bond length 1.9 Å). Besides, compound **4c** weakly formed hydrophobic interactions with Leu694, Val702, Leu764, Thr766, Gly772, Cys773 and Asp776. The other crystal structures of EGFR (PDB entry 6DUK)^41^ were also used for predicting possible binding modes. However, it was quite weaker than the allosteric inhibitor JBJ-04–125-02 (Supplementary Figure S1 and S2). These results indicate that compound **4c** could closely combine with EGFR like the first-generation EGFR inhibitors^42^.

### 3.7. Predicted ADMET and stability studies

Computer-aided drug design (CADD) has been widely used to calculate ADMET for many years^43^. Although there are some limits and disadvantages in predication of ADMET, it would provide some useful information for further studies. Therefore, the ADMET properties of selected compounds **4b**, **4c**, **6e**, **6i**, and **8e–8g** were calculated through Discovery Studio 2.5 software (Accelrys, Inc., San Diego, USA). The calculated results indicated that all the target compounds were less toxic than Gefitinib. As shown in Table 3, the solubility levels of the target compounds were better than Gefitinib except for compound **8e**. However, the absorption of Gefitinib was significantly potent than the target compounds. CYP2D6 (non-inhibitor of cytochrome P450 enzyme) is always used to predict drug toxicity^44^. In the meantime, the calculated hepatotoxicity values, PPB values and log *p* values of target compounds showed well oral bioavailability, which were a little lower than Gefitinib.

**Table 3.** Predicted ADMET properties of the target compounds.

Comp.	Solubility	Absorption	CYP2D6	Hepatotoxicity	PPB	AlogP98
**4b**	1	2	0	1	2	5.19
**4c**	1	2	0	1	2	5.19
**6e**	1	2	0	0	2	4.32
**6i**	1	3	0	0	0	3.68
**8e**	2	3	0	1	1	4.24
**8f**	1	3	0	1	1	4.44
**8g**	1	3	0	1	1	4.44
**Gefitinib**	2	0	1	0	1	4.20

## 4. Conclusion

In conclusion, a novel series of dianilinopyrimidines as EGFR inhibitors were designed and synthesised. All the target compounds were confirmed by ^1^H-NMR, ^13 ^C-NMR, and HRMS. And then, these compounds were tested for inhibitory effects against EGFR and tumour cells (A549, PC-3, HepG2). The results showed that some of the compounds performed well in anti-tumour activities. In particular, compound **4c** showed the best activities against all tumour cells (IC_50_ of 0.56 μM, 2.46 μM, and 2.21 μM, respectively). Further studies indicated that compound **4c** could induce apoptosis A549 cells and arrest A549 cells in the G2/M phase. In addition, molecular docking and ADMET of compound **4c** were also investigated.

## Supplementary Material

Supplemental Material
