# Supplementary material for: Design, synthesis and biological evaluation of a series of dianilinopyrimidines as EGFR inhibitors
Source: J Enzyme Inhib Med Chem. 2022 Mar 9;37(1):832–43. doi: 10.1080/14756366.2022.2046567 (PMC8920385; doi:10.1080/14756366.2022.2046567)
Supplement: Supplemental Material [file IENZ_A_2046567_SM3971.pdf]

Supporting Information

Design, synthesis and biological evaluation of a series of dianilinopyrimidines as EGFR inhibitors

Longjia Yan [1,2,3], Qin Wang [1,3], Li Liu [1,3], Yi Le [1,2,3,*]

[1] School of Pharmaceutical Sciences, Guizhou University, Guiyang 550025

[2] State Key Laboratory of Functions and Applications of Medicinal Plants, Guizhou Medical University, Guiyang 550014, China

[3] Guizhou Engineering Laboratory for Synthetic Drugs, Guiyang 550025, China

E-mail: yile2021@163.com (Yi Le).

Table of contents

Molecular Docking ……………………………………….……………………………..…..**2**

NMR and HRMS spectra ……………………………………………………………..…..**3-43**

The IC_{50} curves from Table 2…………………………………………………...……...**44-47**

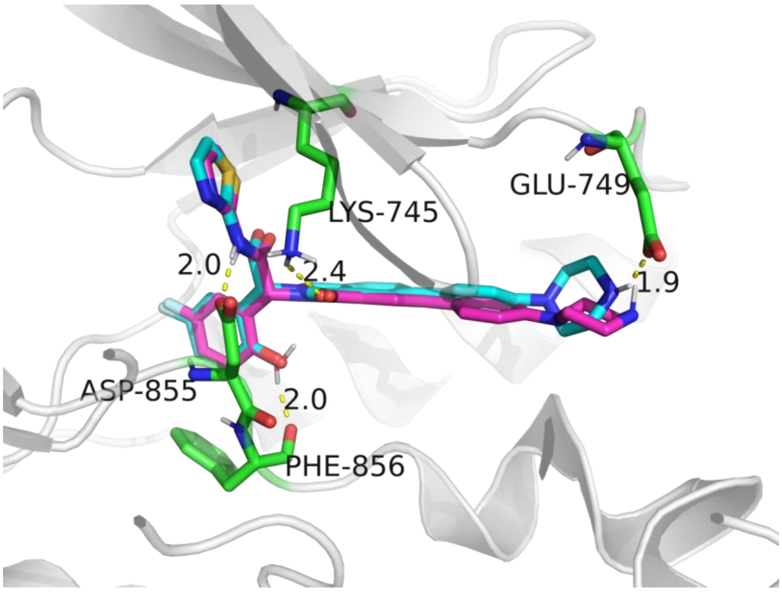

Figure S1. Binding configuration of **JBJ-04-125-02** with EGFR (PDB: 6DUK).

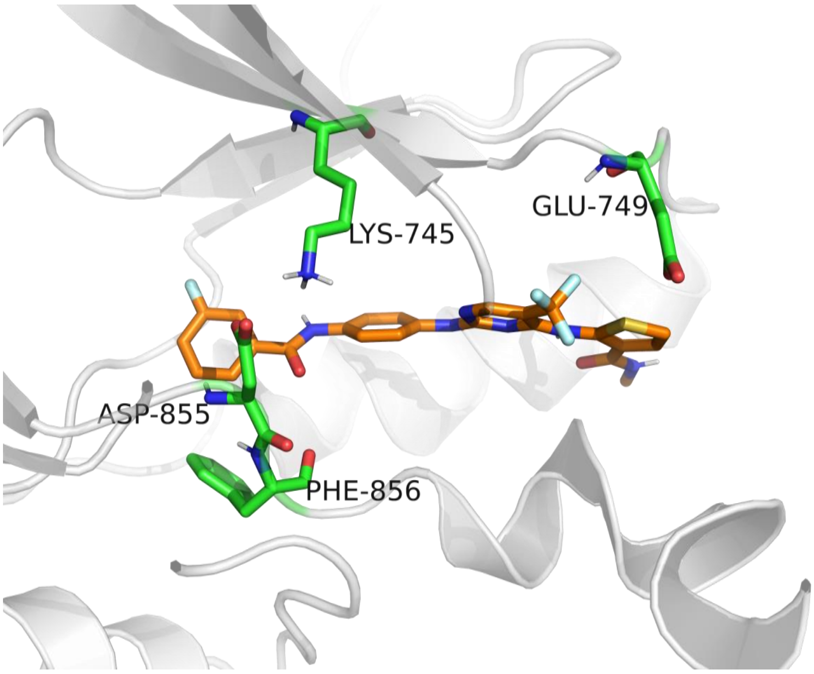

Figure S2. Binding configuration of compound **4c** with EGFR (PDB: 6DUK).

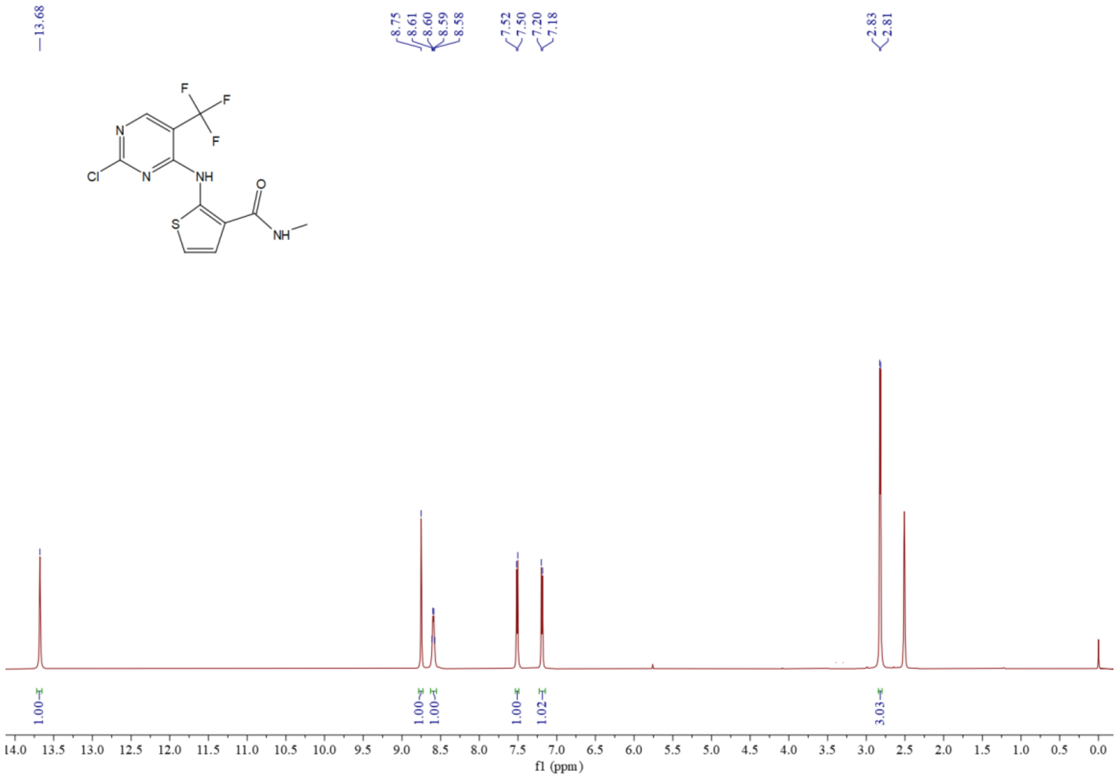

Figure S3. ^{1}H NMR spectrum of compound **1**

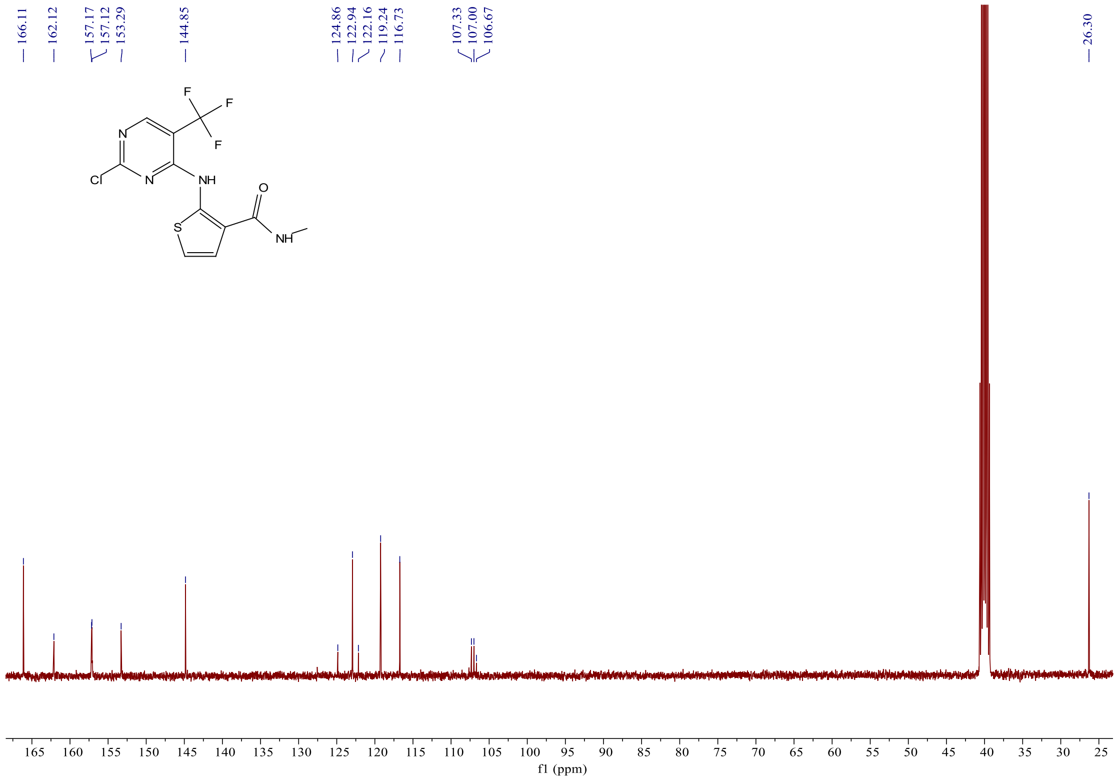

Figure S4. ^{13}C NMR spectrum of compound **1**

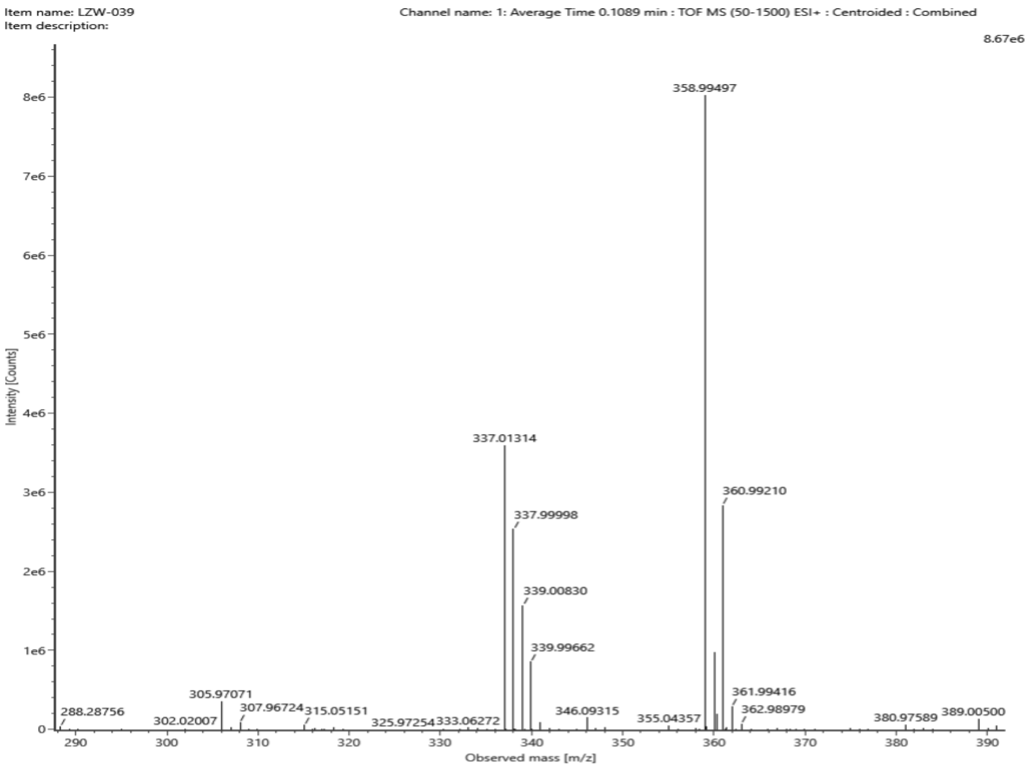

Figure S5. HRMS spectrum of compound **1**

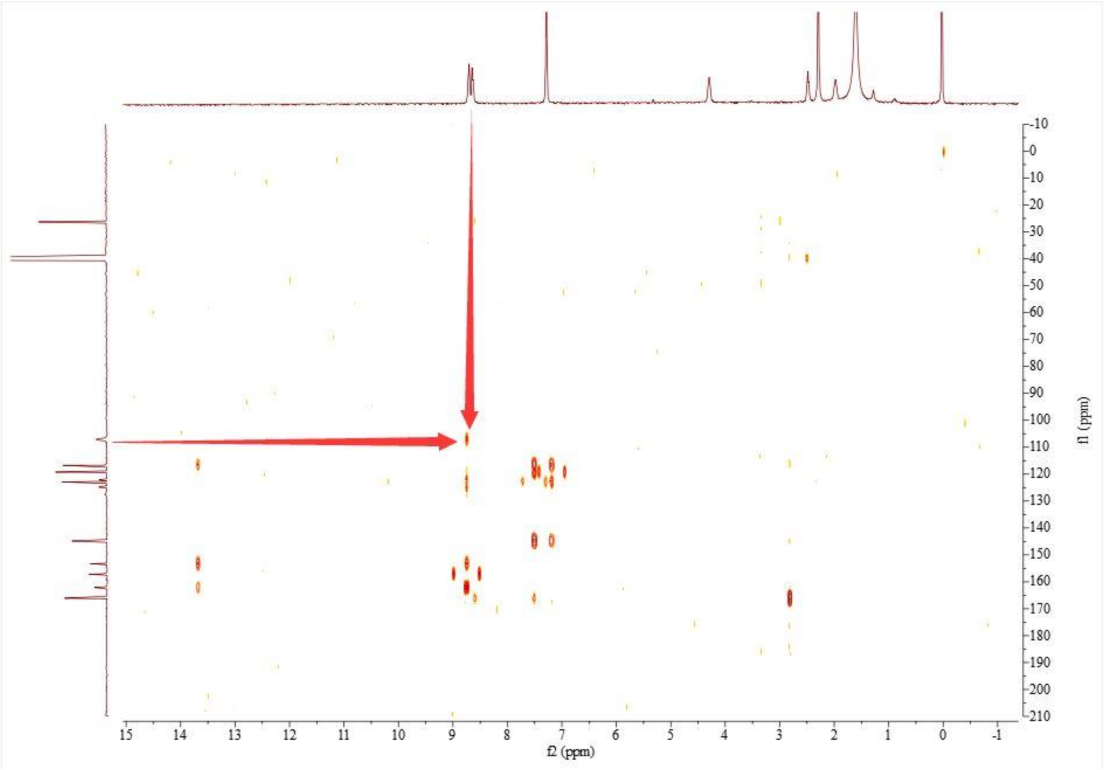

Figure S6. HMBC spectrum of compound **1**

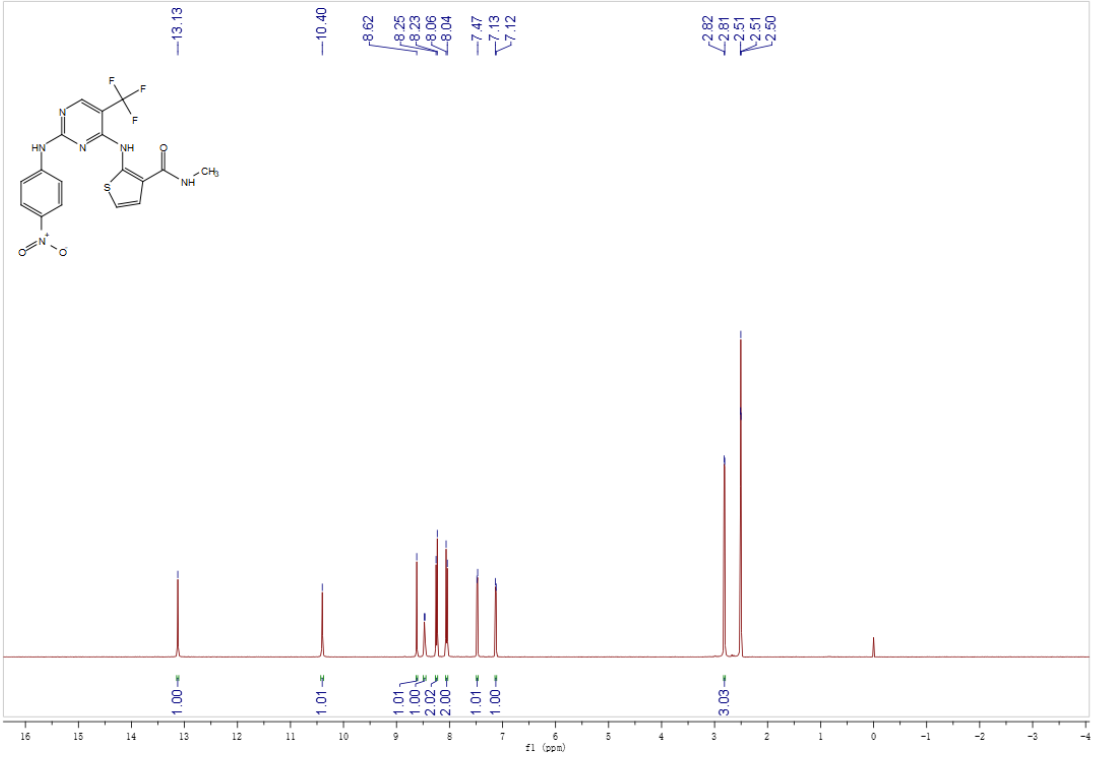

Figure S7. ¹H NMR spectrum of compound **2**

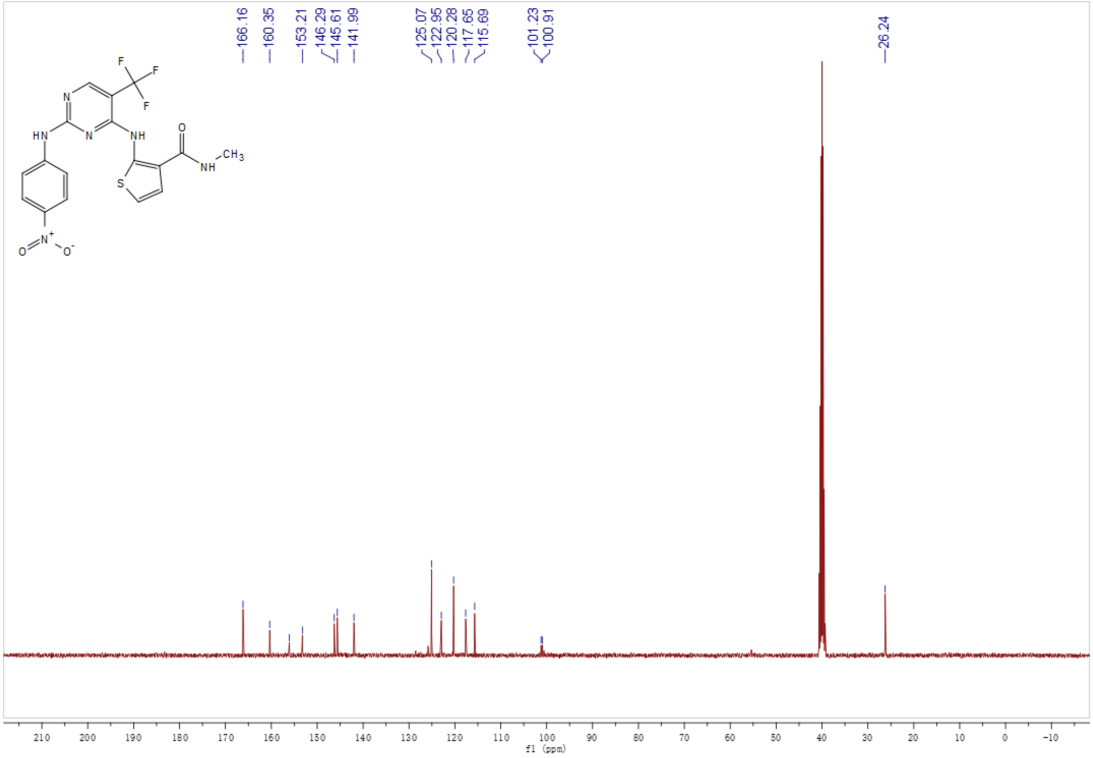

Figure S8. ¹³C NMR spectrum of compound **2**

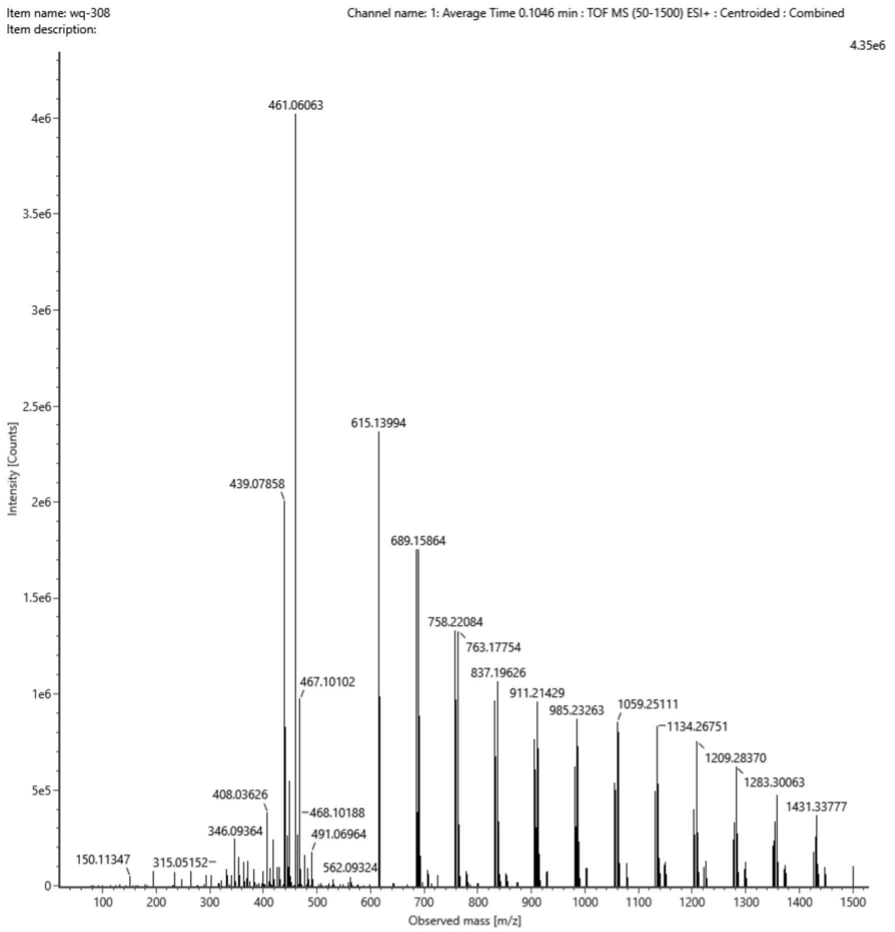

Figure S9. HRMS spectrum of compound **2**

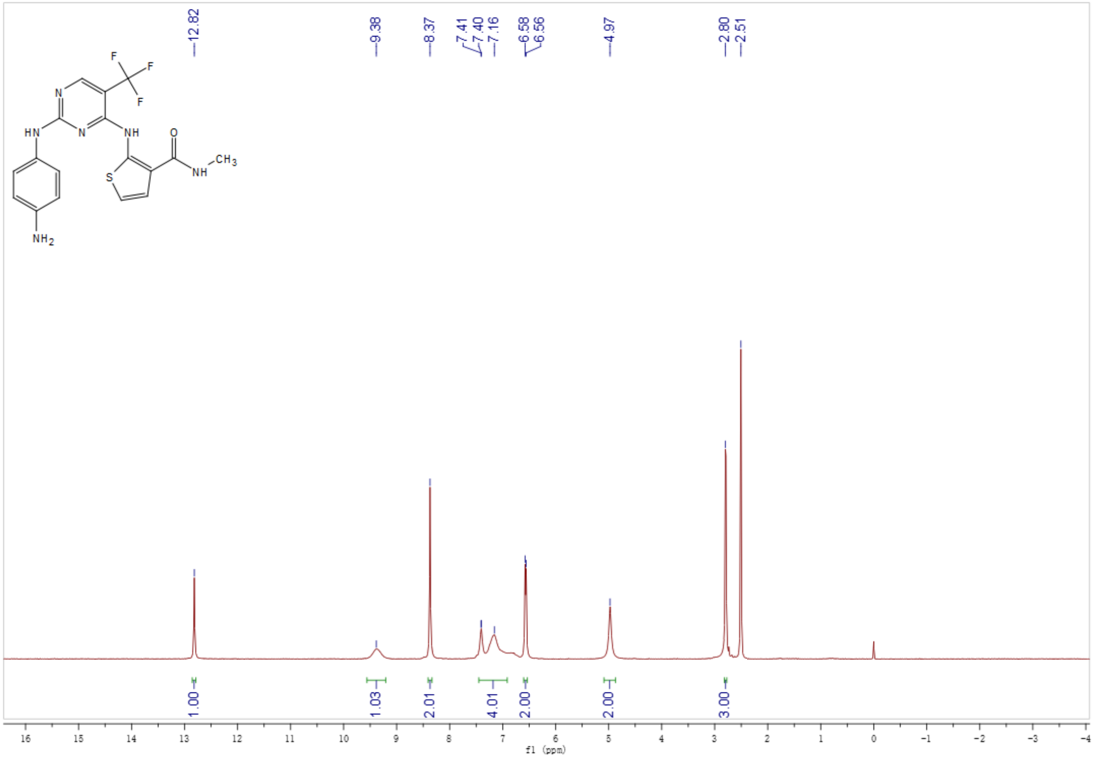

Figure S10. ^{1}H NMR spectrum of compound **3**

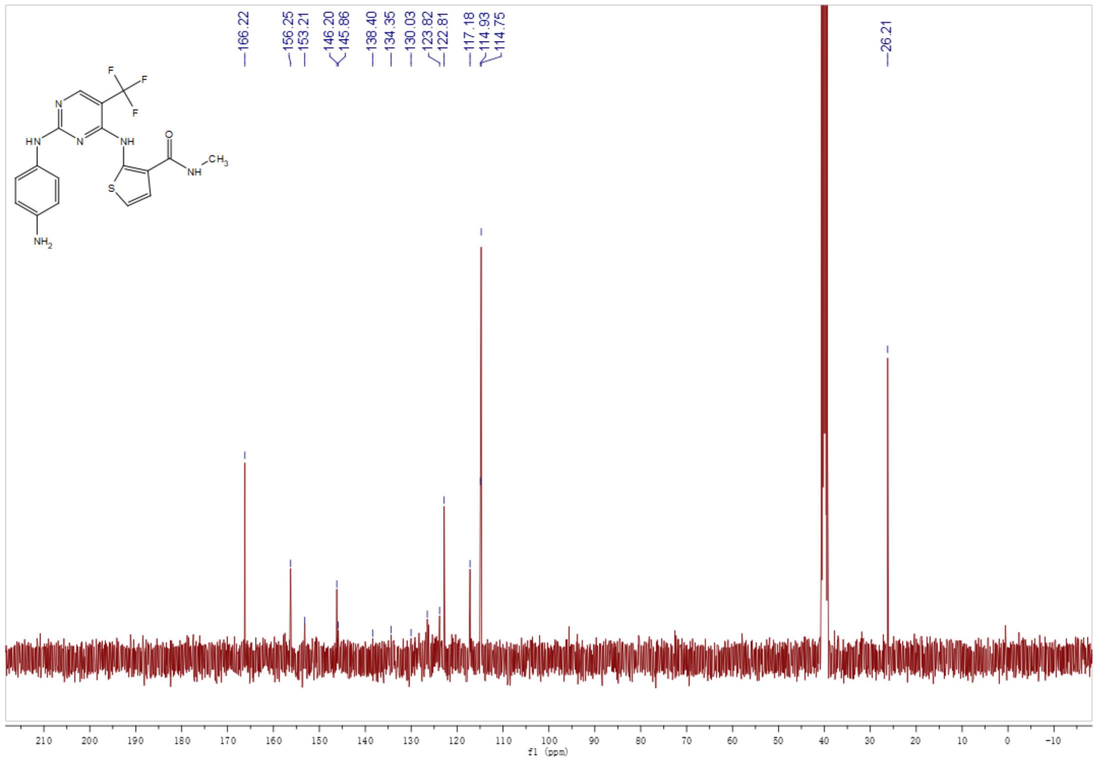

Figure S11. ^{13}C NMR spectrum of compound **3**

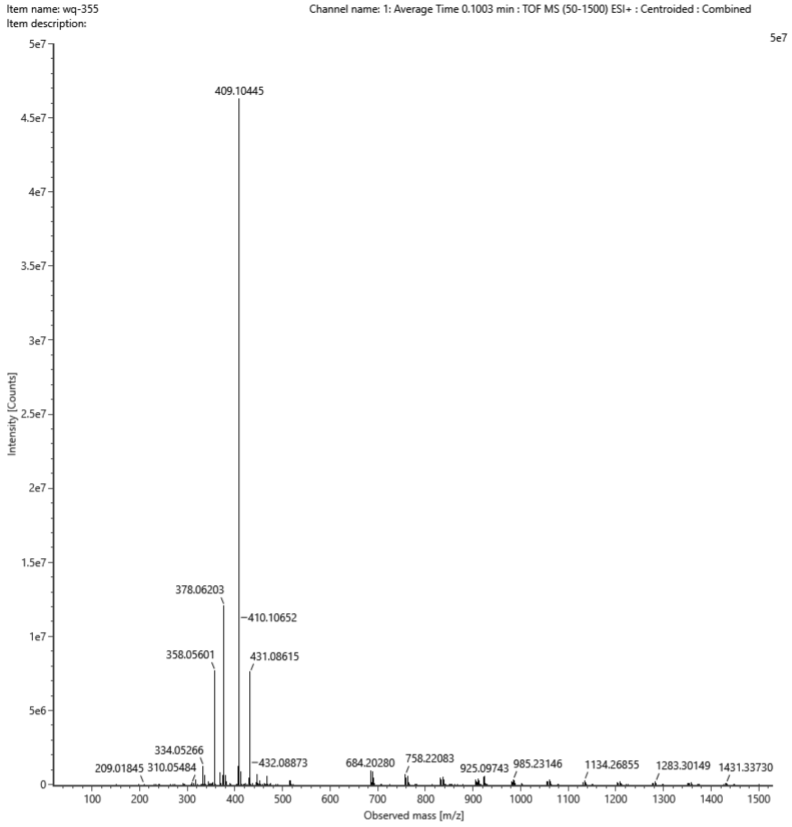

Figure S12. HRMS spectrum of compound **3**

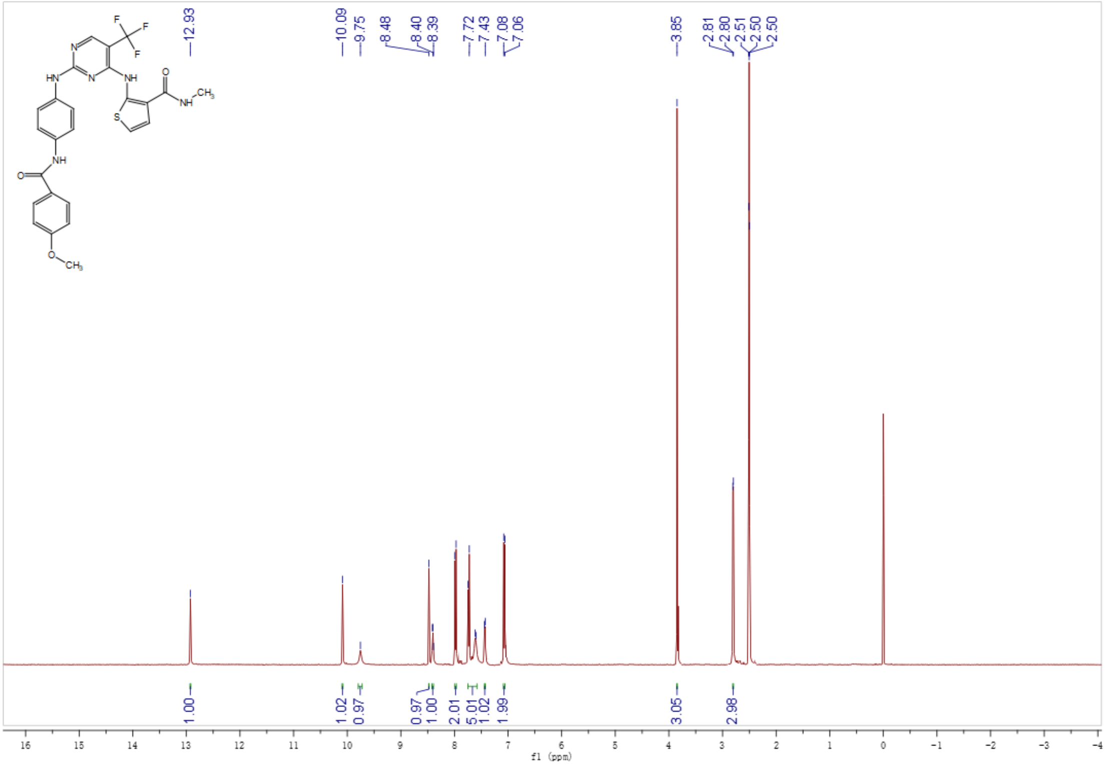

Figure S13. ^{1}H NMR spectrum of compound **4a**

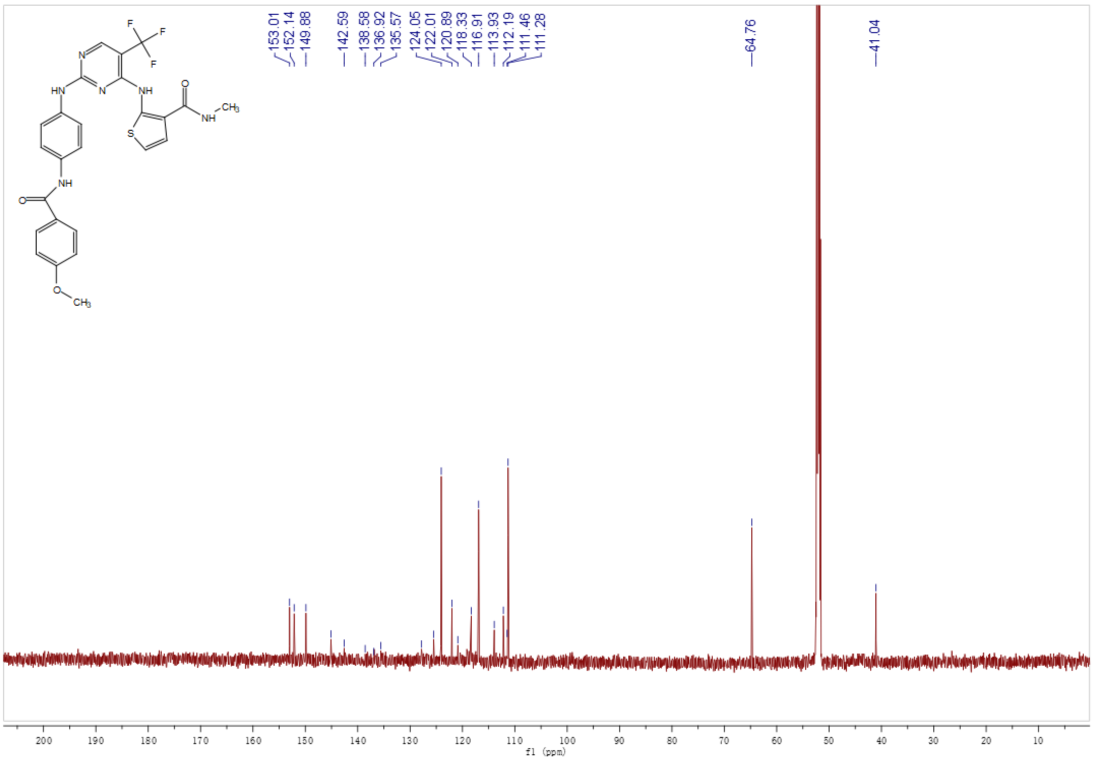

Figure S14. ^{13}C NMR spectrum of compound **4a**

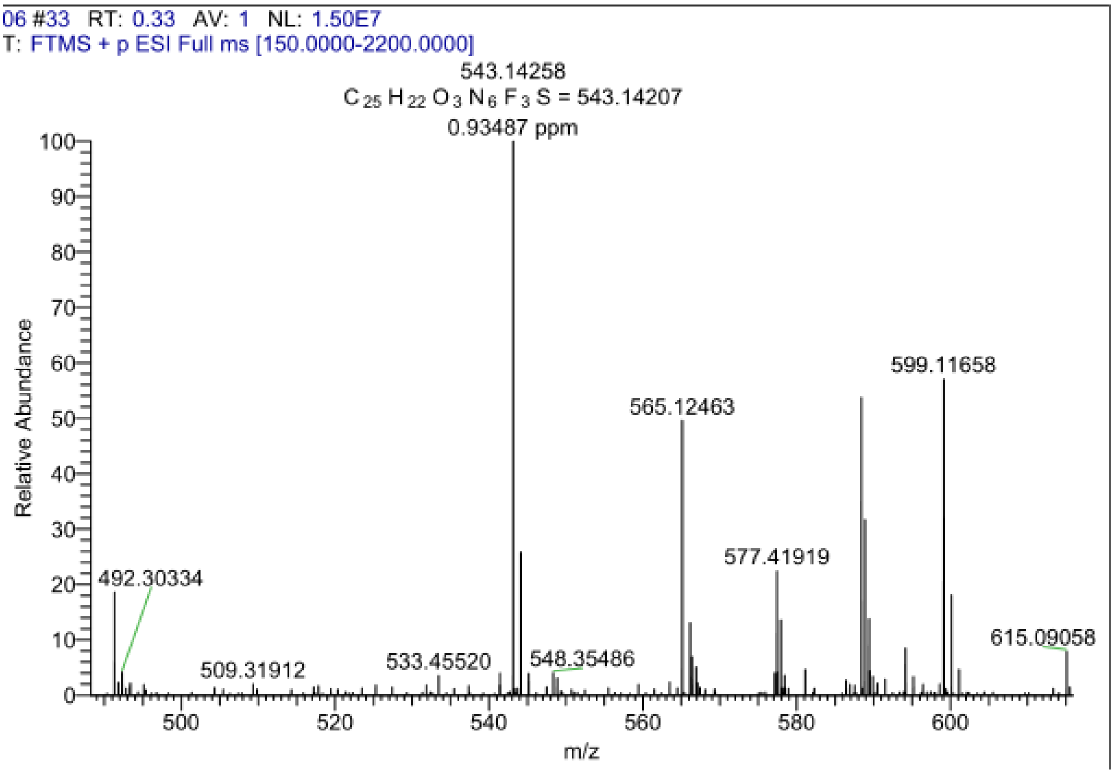

Figure S15. HRMS spectrum of compound **4a**

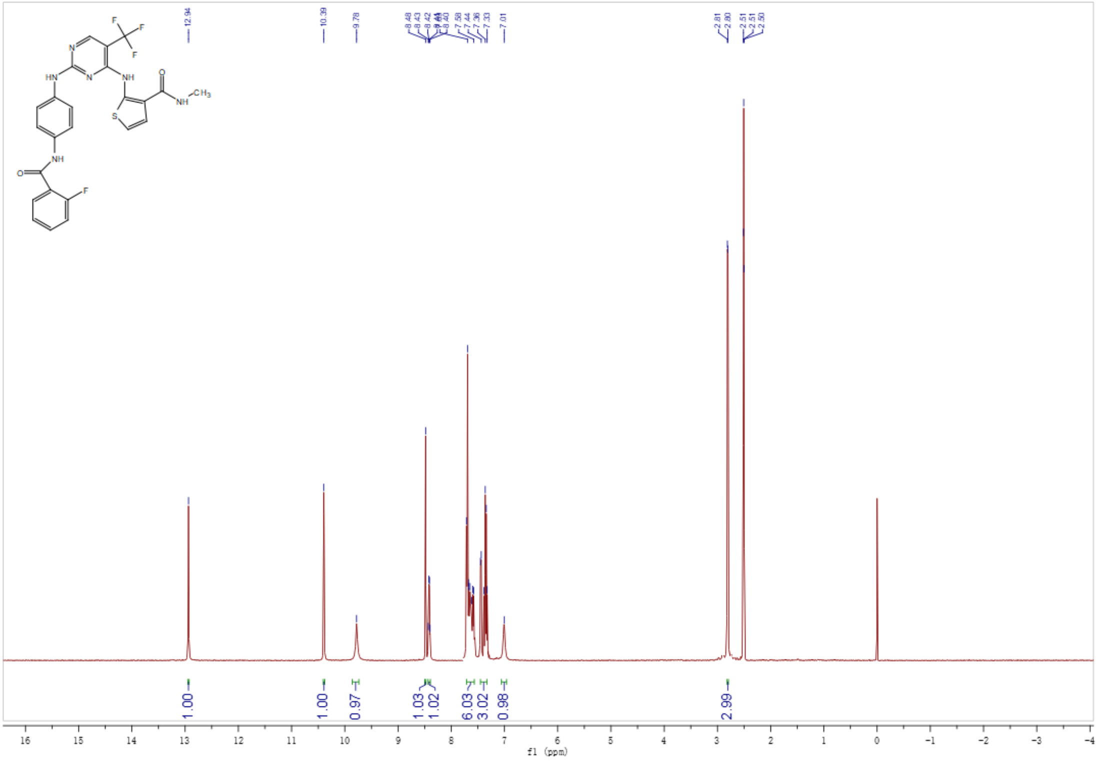

Figure S16. ^{1}H NMR spectrum of compound **4b**

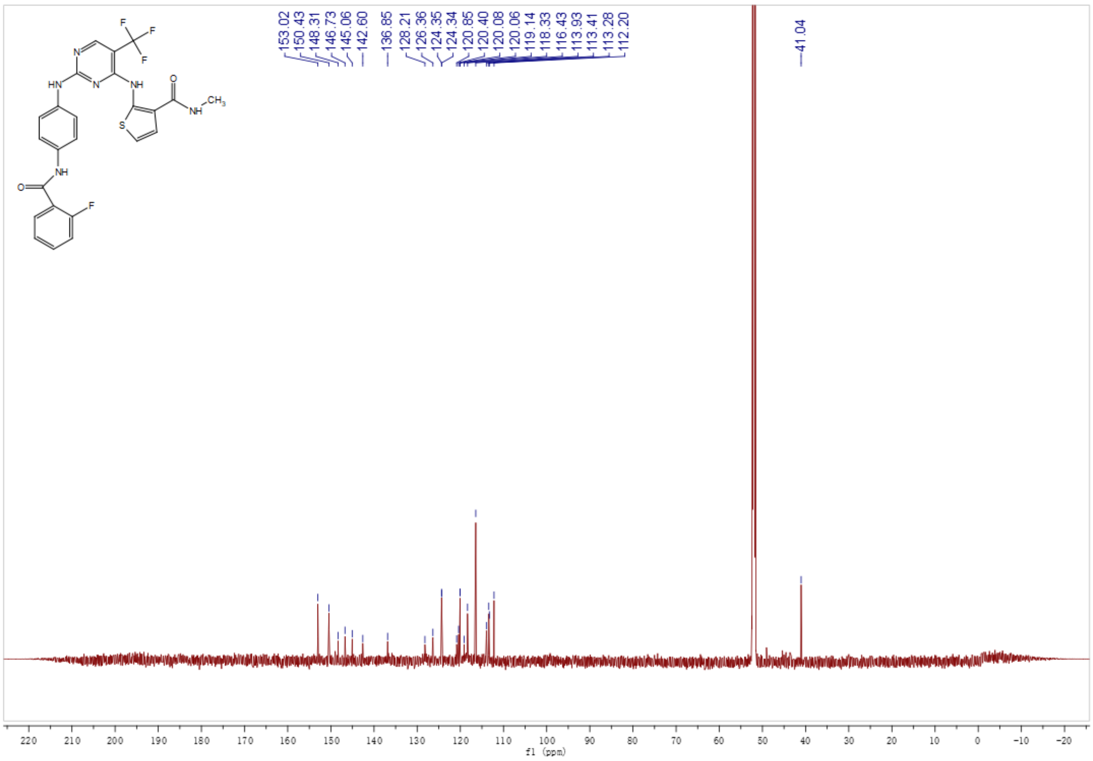

Figure S17. ^{13}C NMR spectrum of compound **4b**

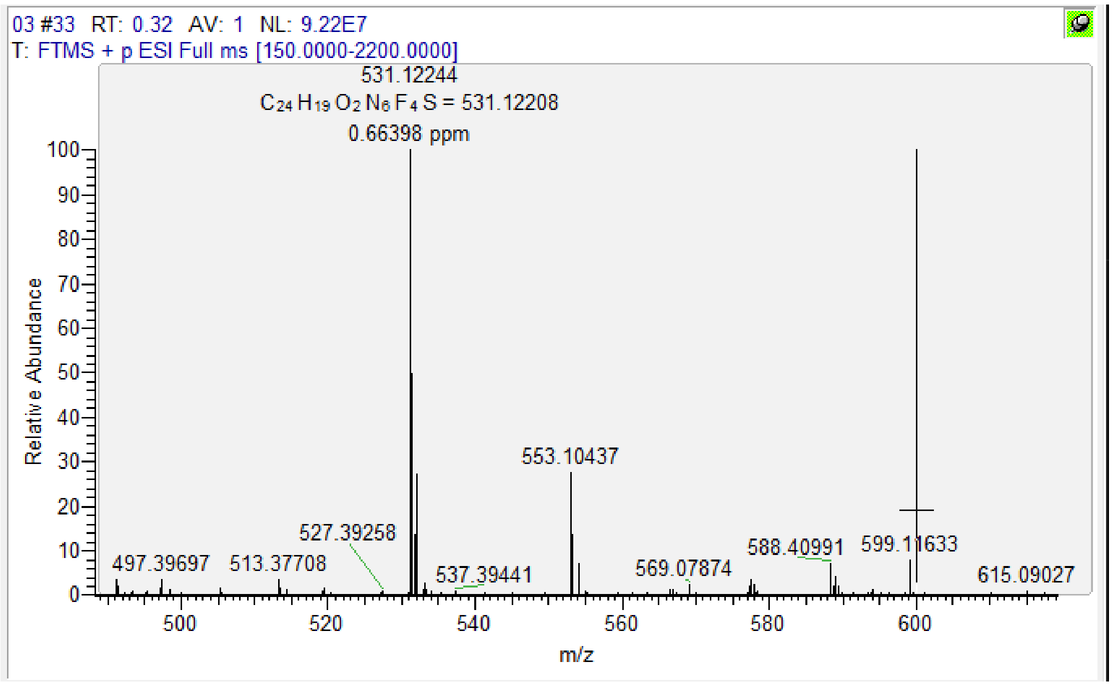

Figure S18. HRMS spectrum of compound **4b**

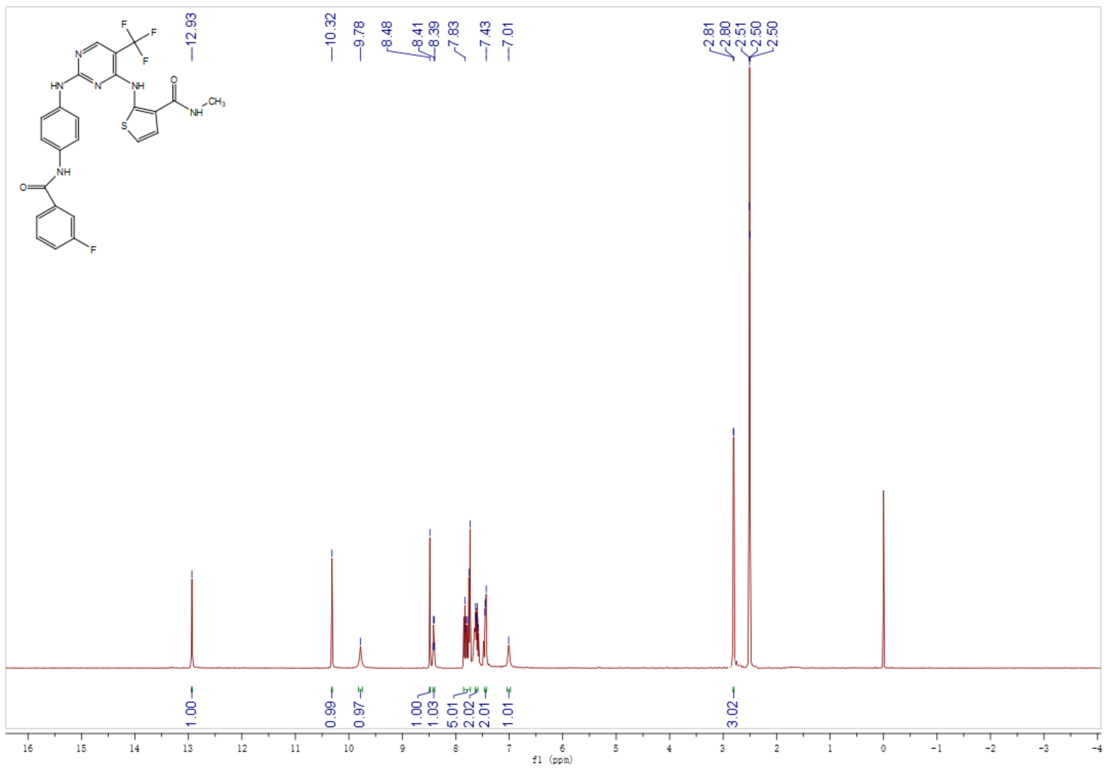

Figure S19. ^{1}H NMR spectrum of compound **4c**

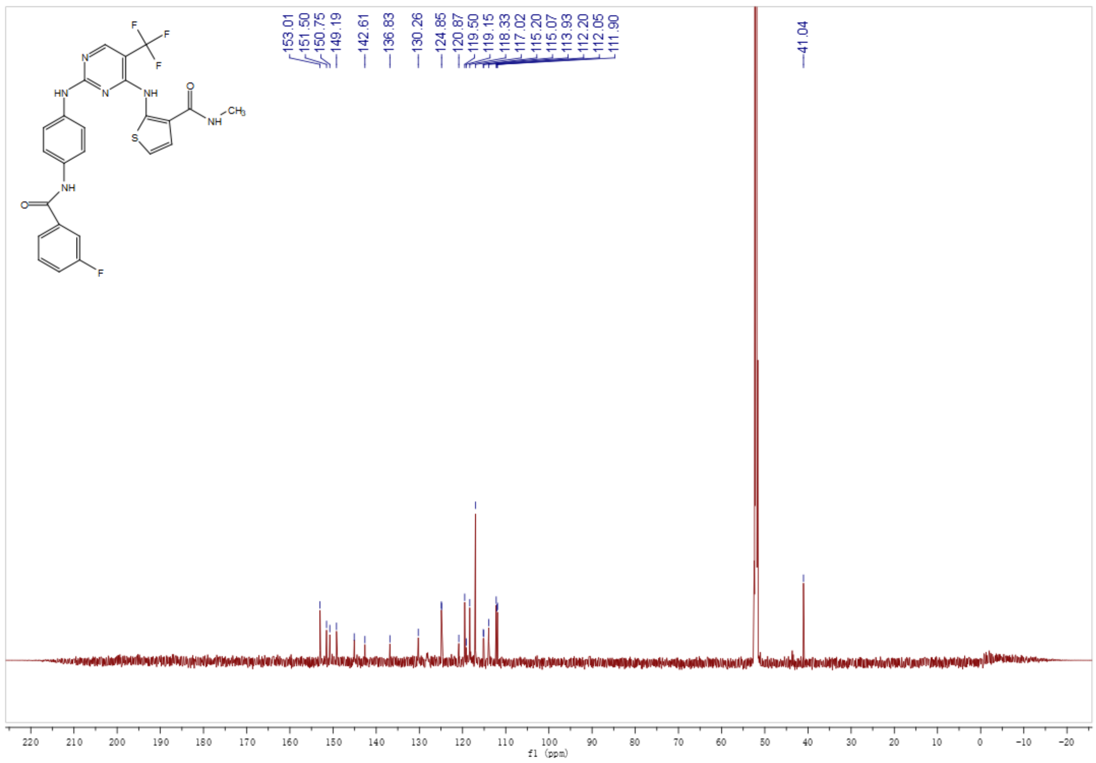

Figure S20. ^{13}C NMR spectrum of compound **4c**

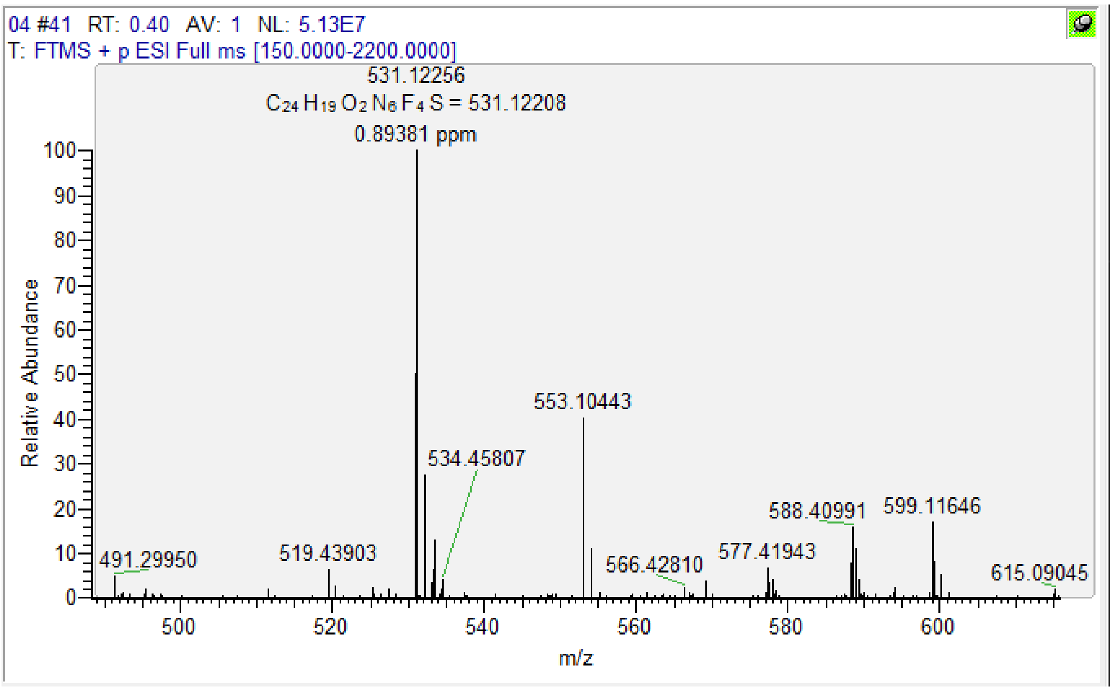

Figure S21. HRMS spectrum of compound **4c**

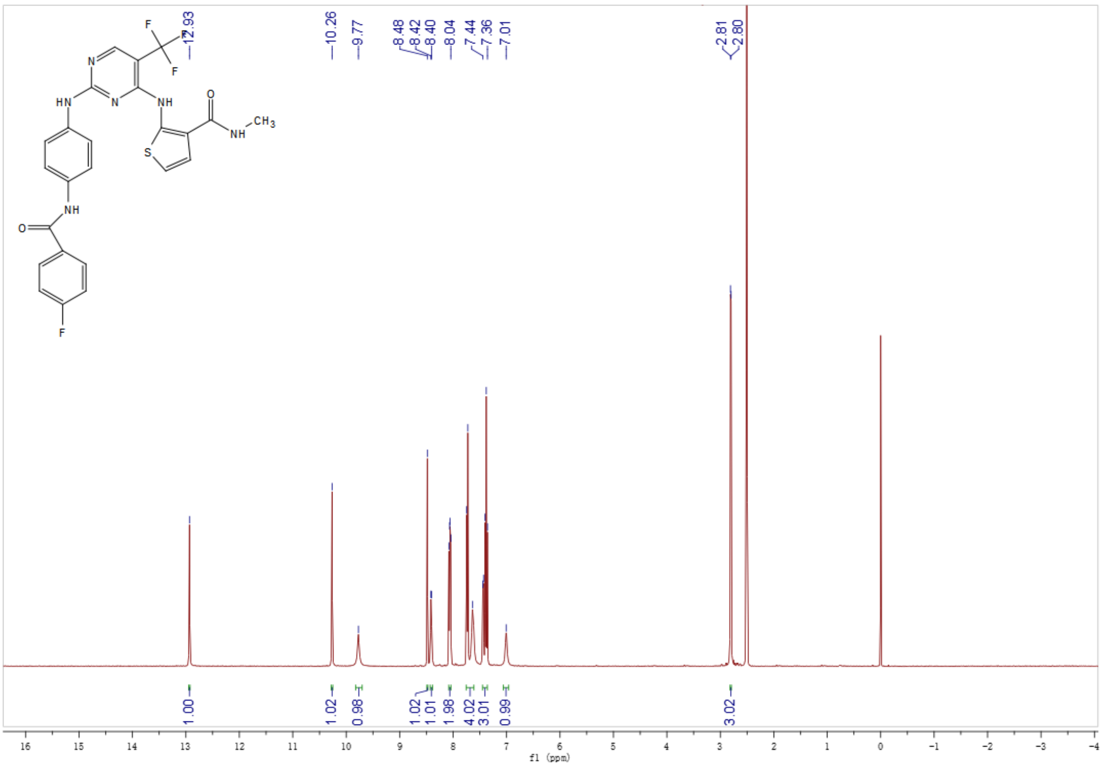

Figure S22. ^{1}H NMR spectrum of compound **4d**

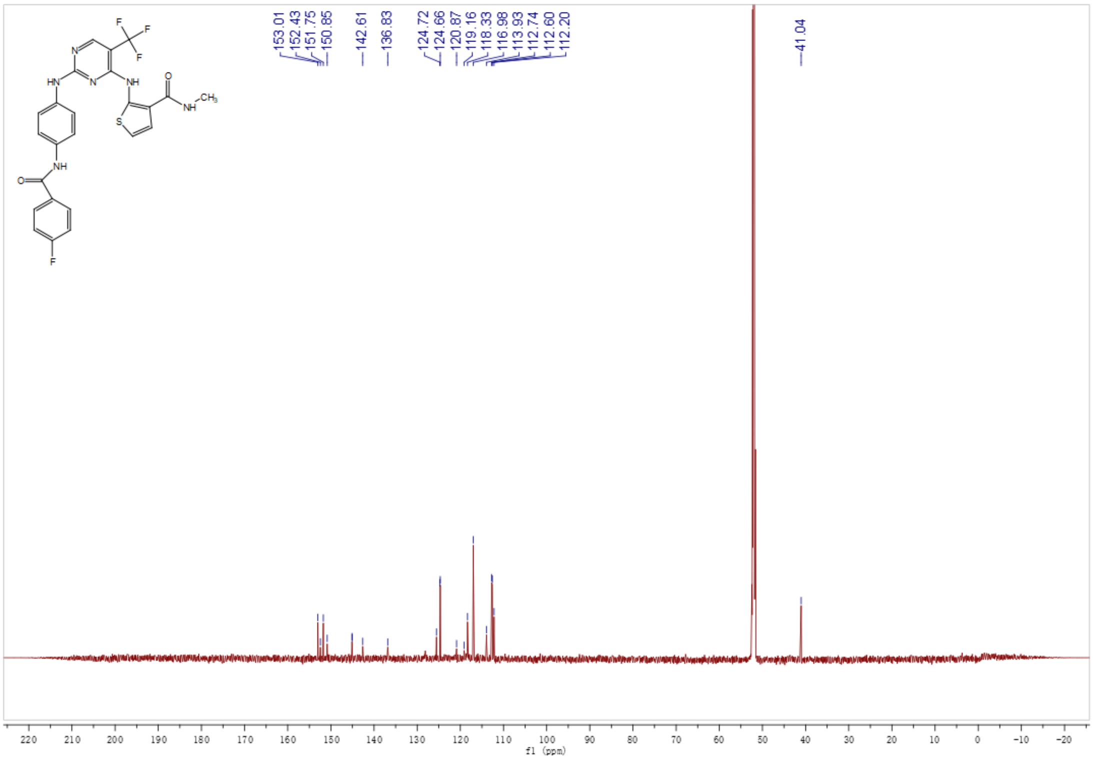

Figure S23. ^{13}C NMR spectrum of compound **4d**

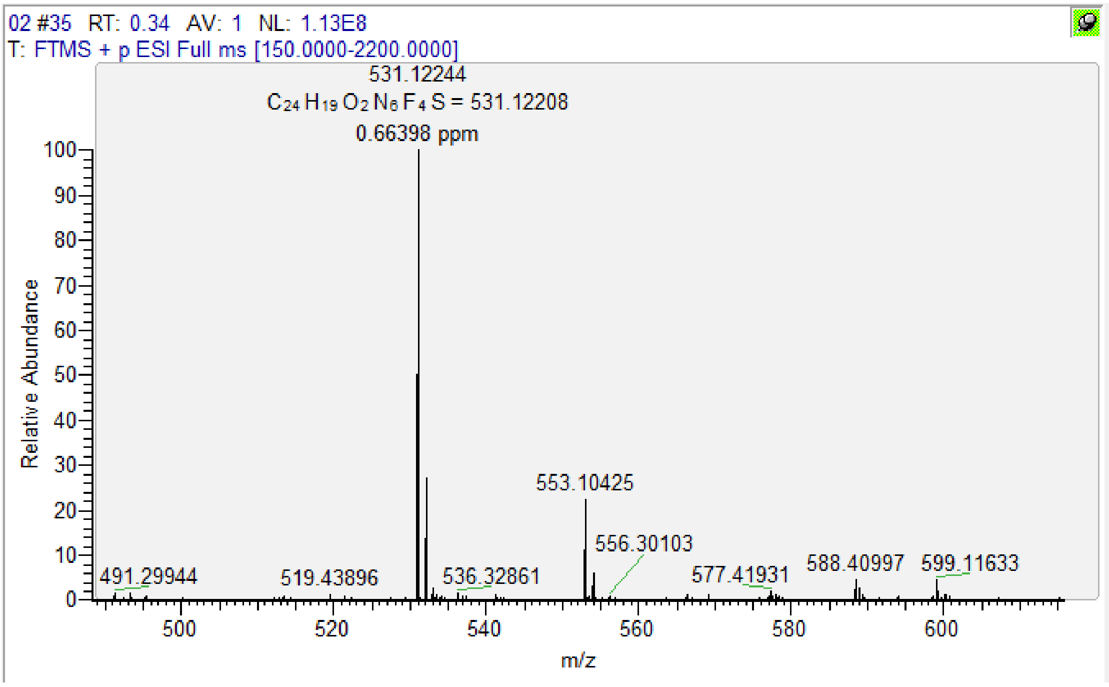

Figure S24. HRMS spectrum of compound **4d**

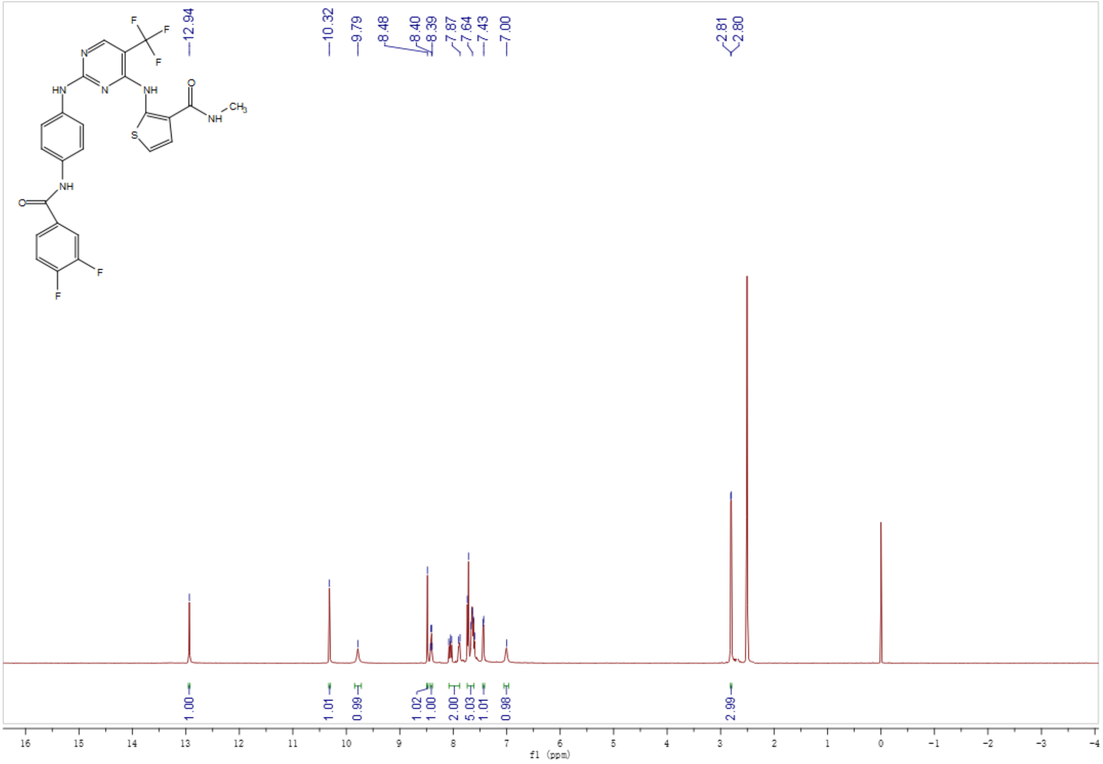

Figure S25. ^{1}H NMR spectrum of compound **4e**

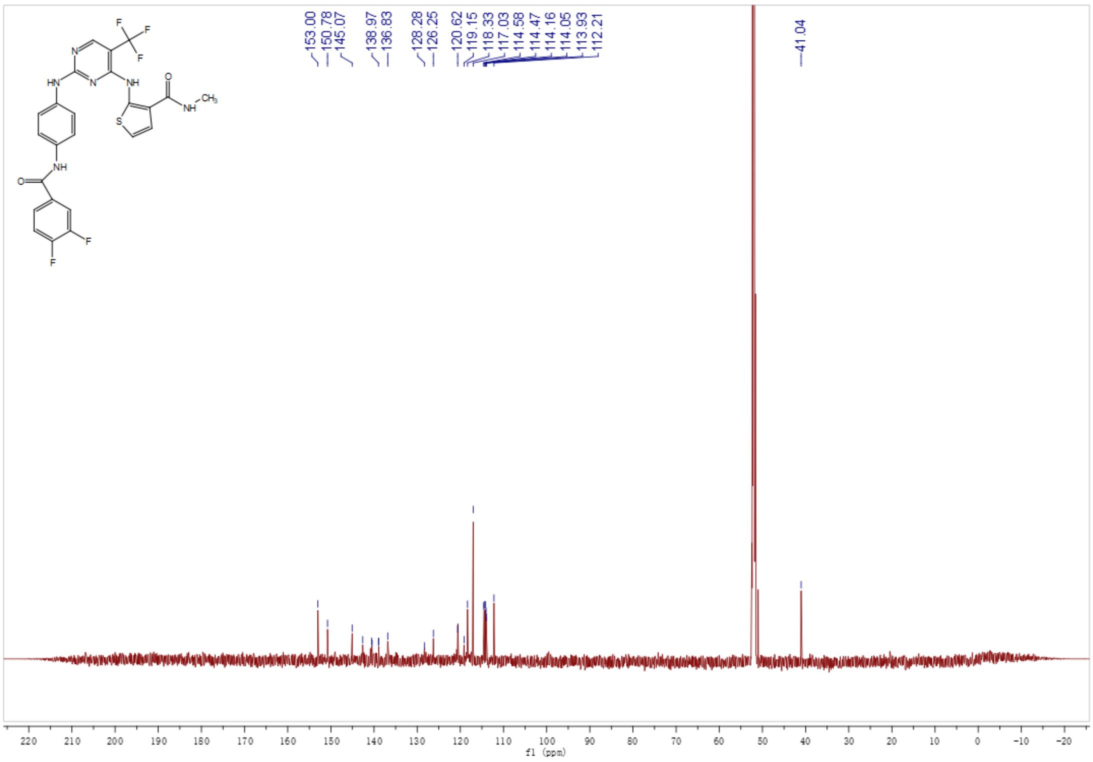

Figure S26. ^{13}C NMR spectrum of compound **4e**

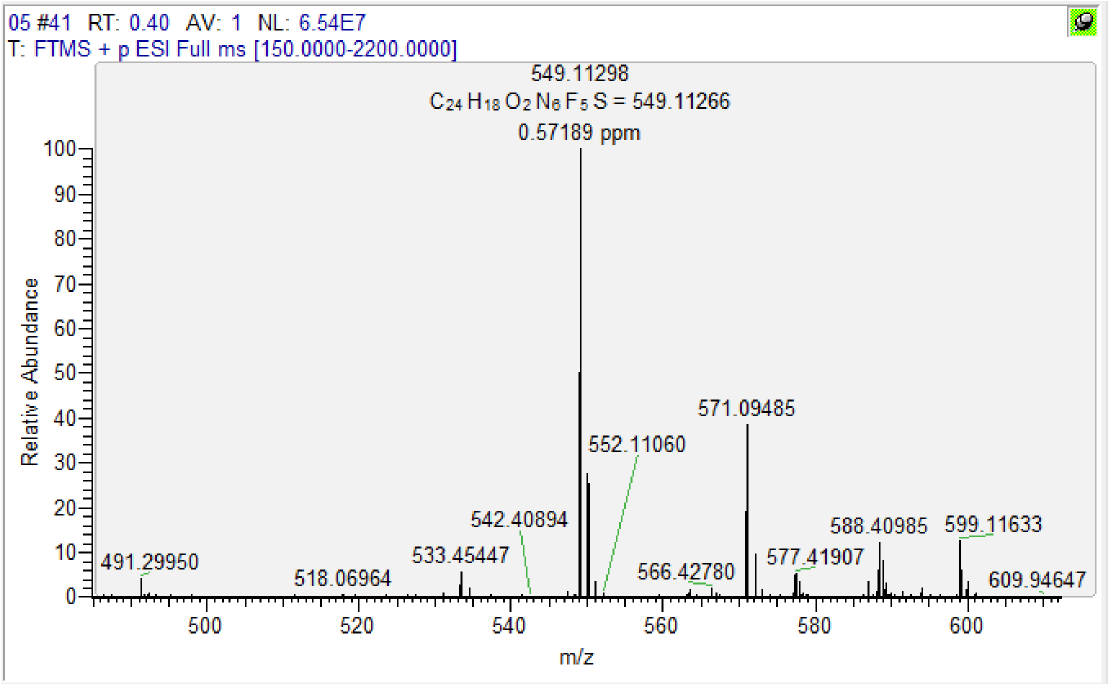

Figure S27. HRMS spectrum of compound **4e**

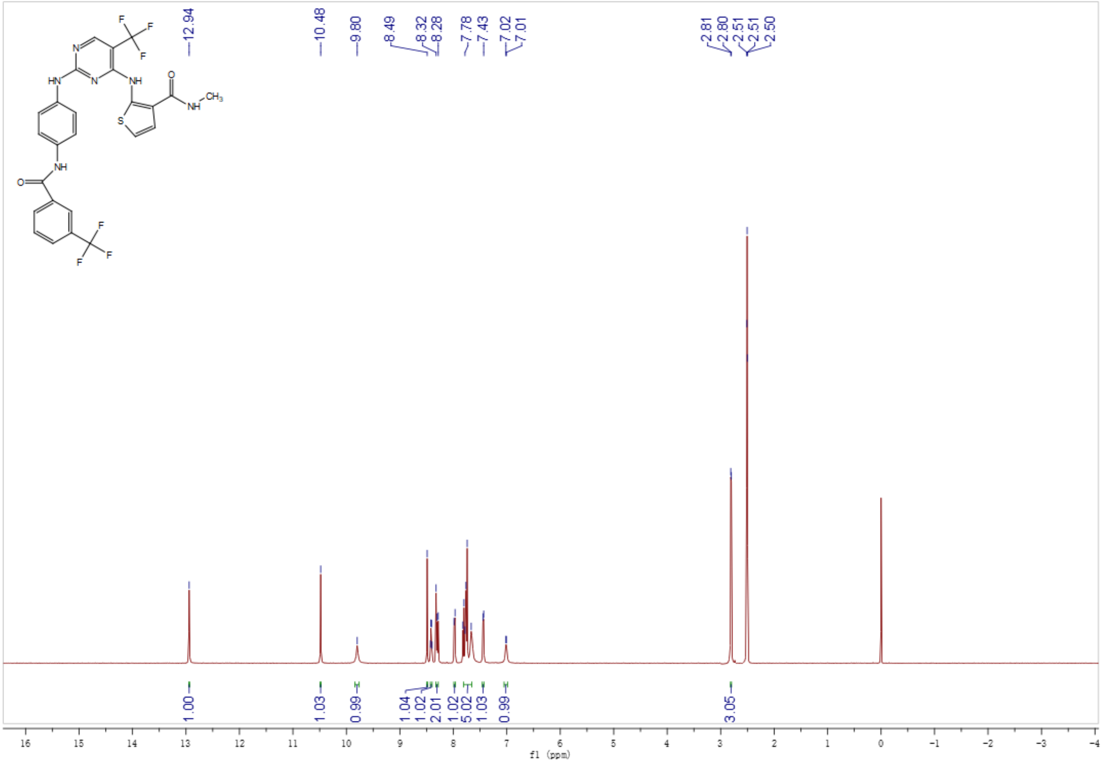

Figure S28. ^{1}H NMR spectrum of compound **4f**

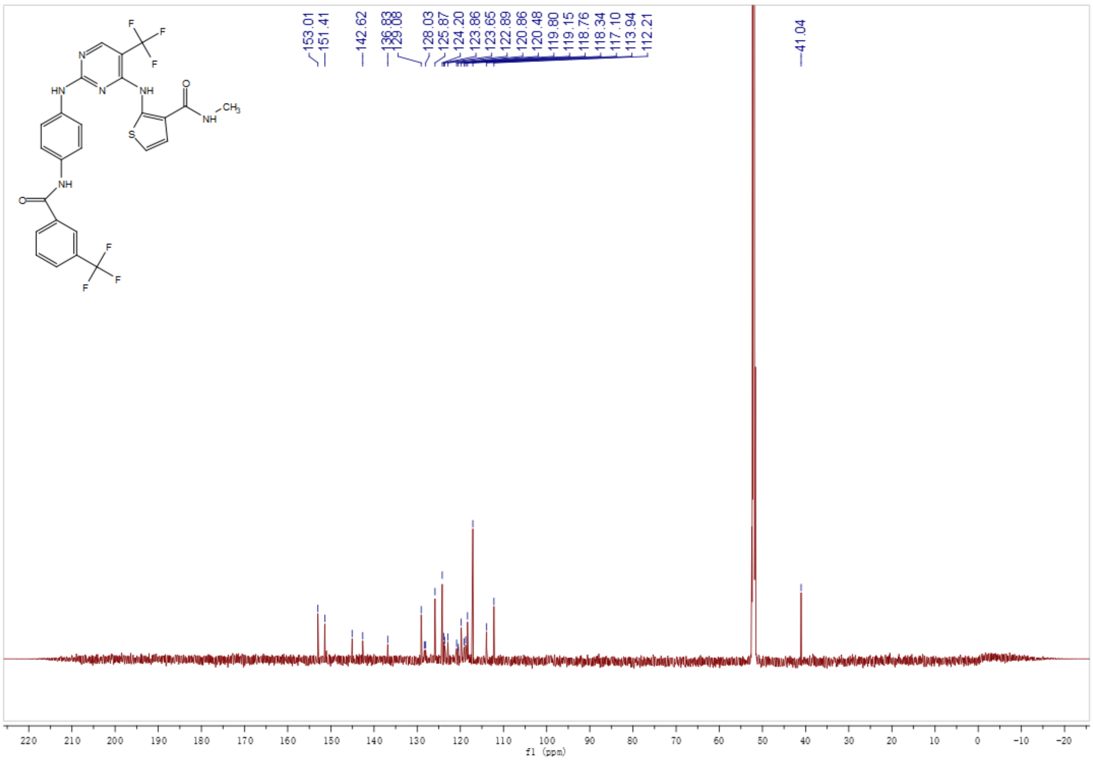

Figure S29. ^{13}C NMR spectrum of compound **4f**

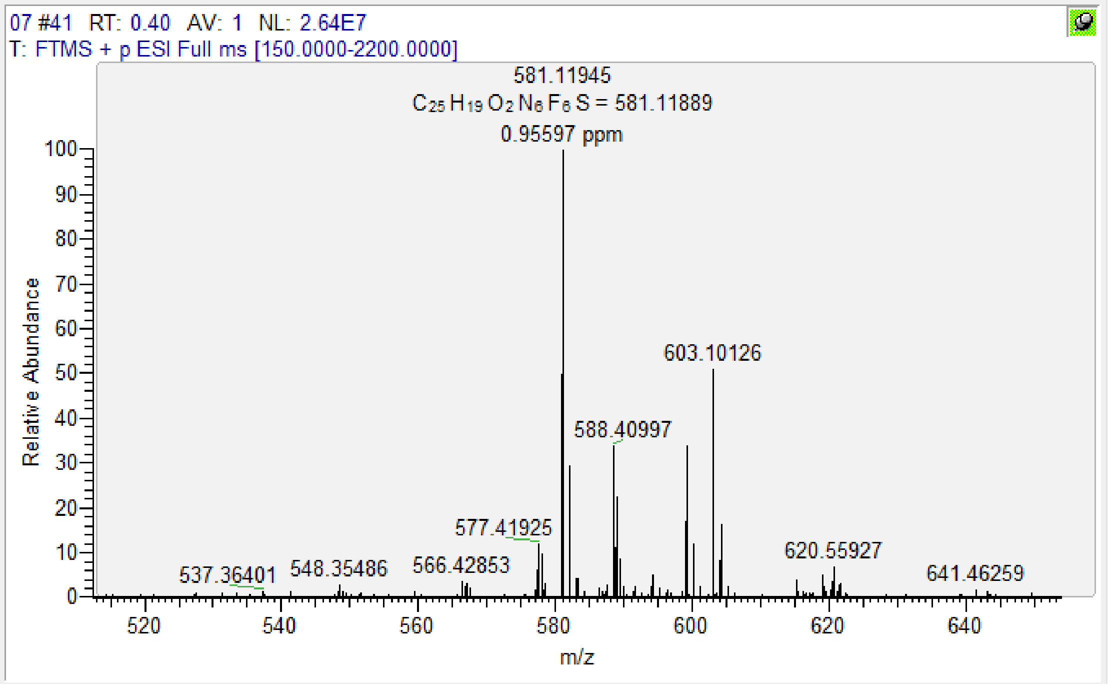

Figure S30. HRMS spectrum of compound **4f**

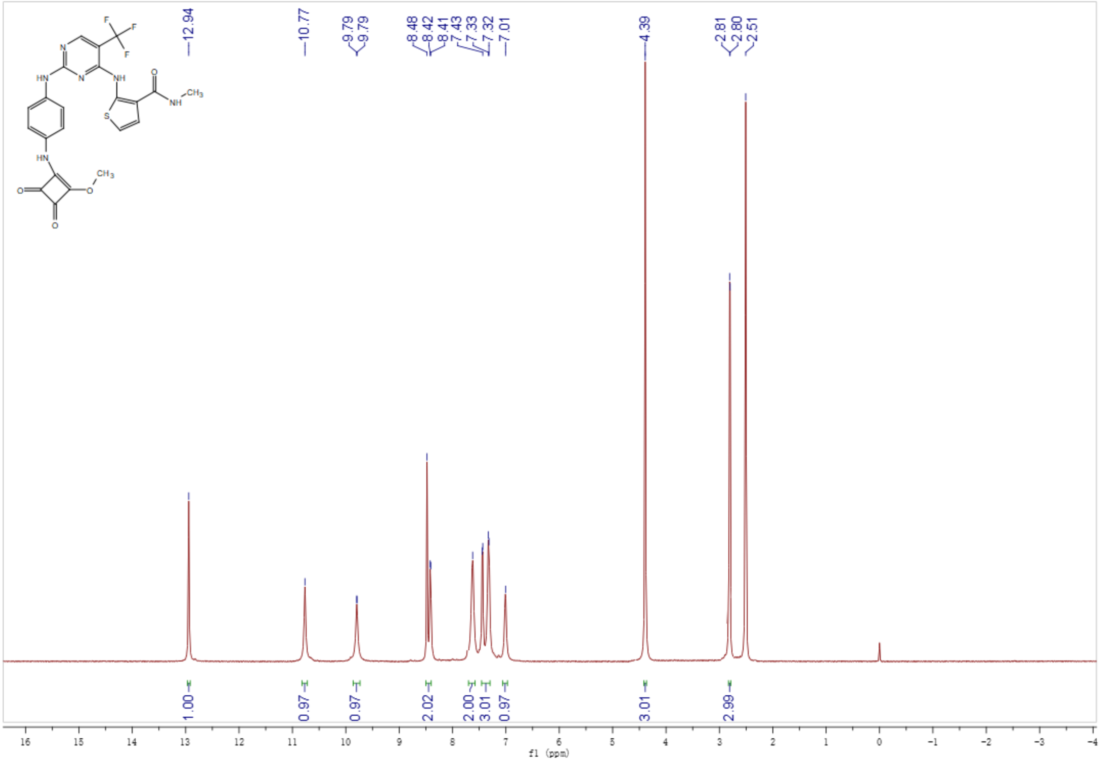

Figure S31. ^{1}H NMR spectrum of compound **5**

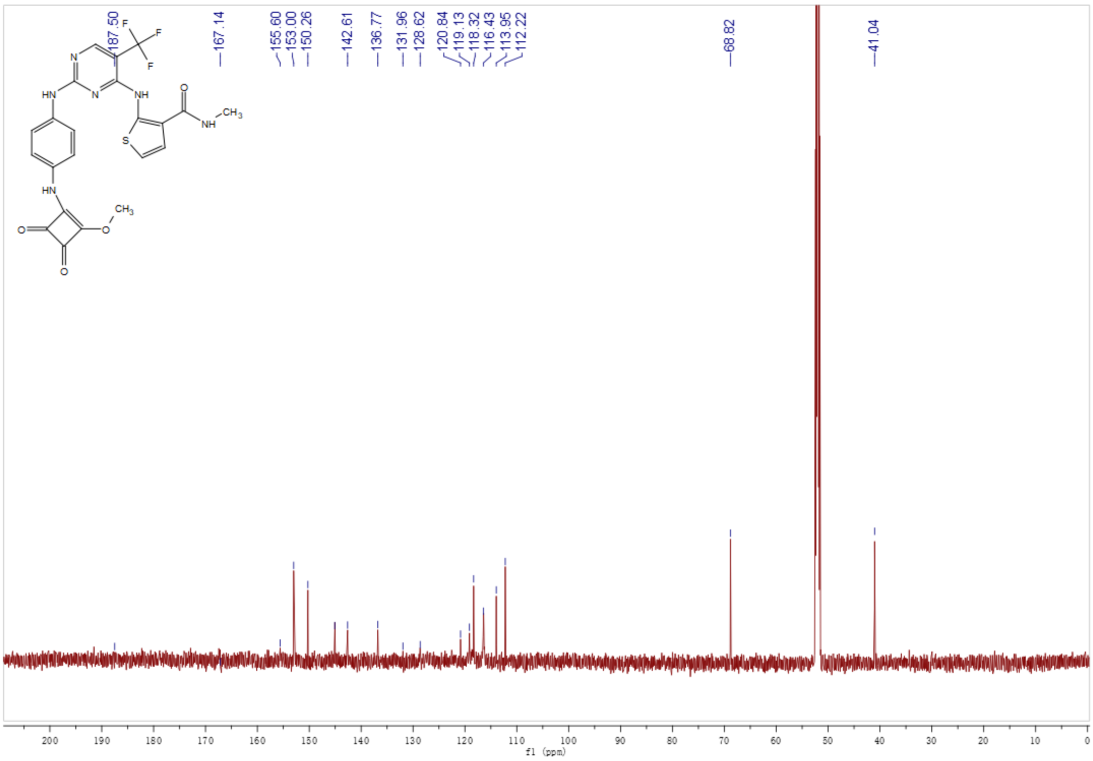

Figure S32. ^{13}C NMR spectrum of compound **5**

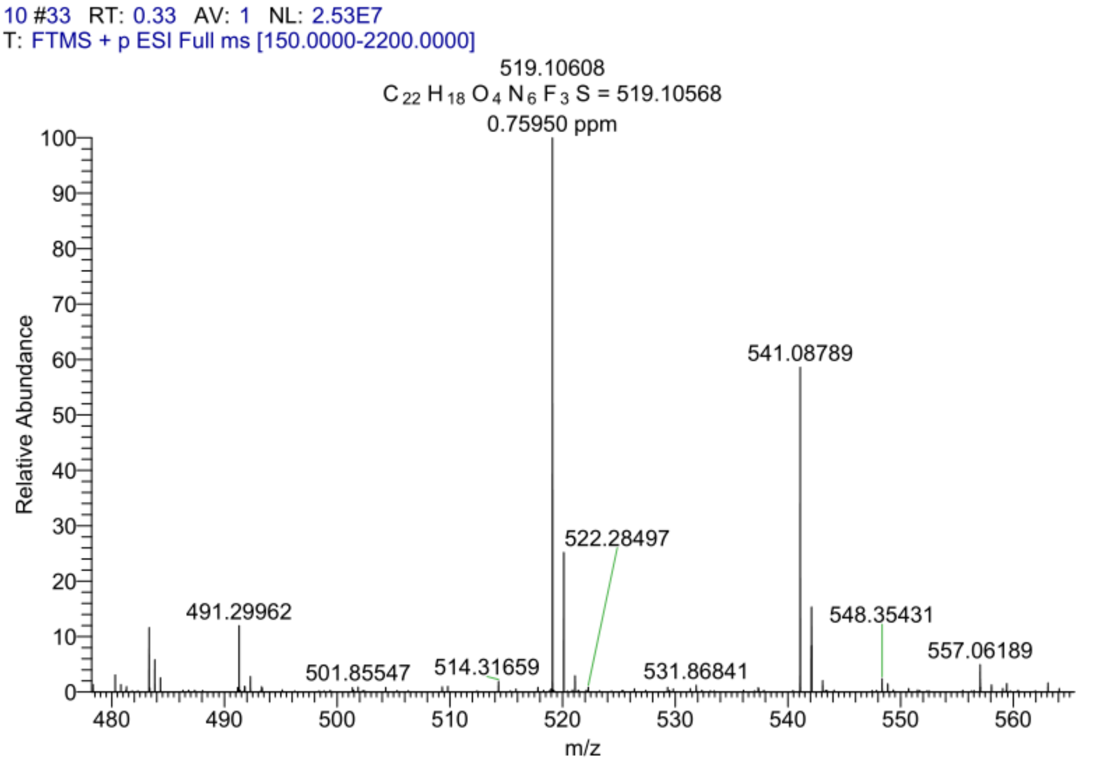

Figure S33. HRMS spectrum of compound **5**

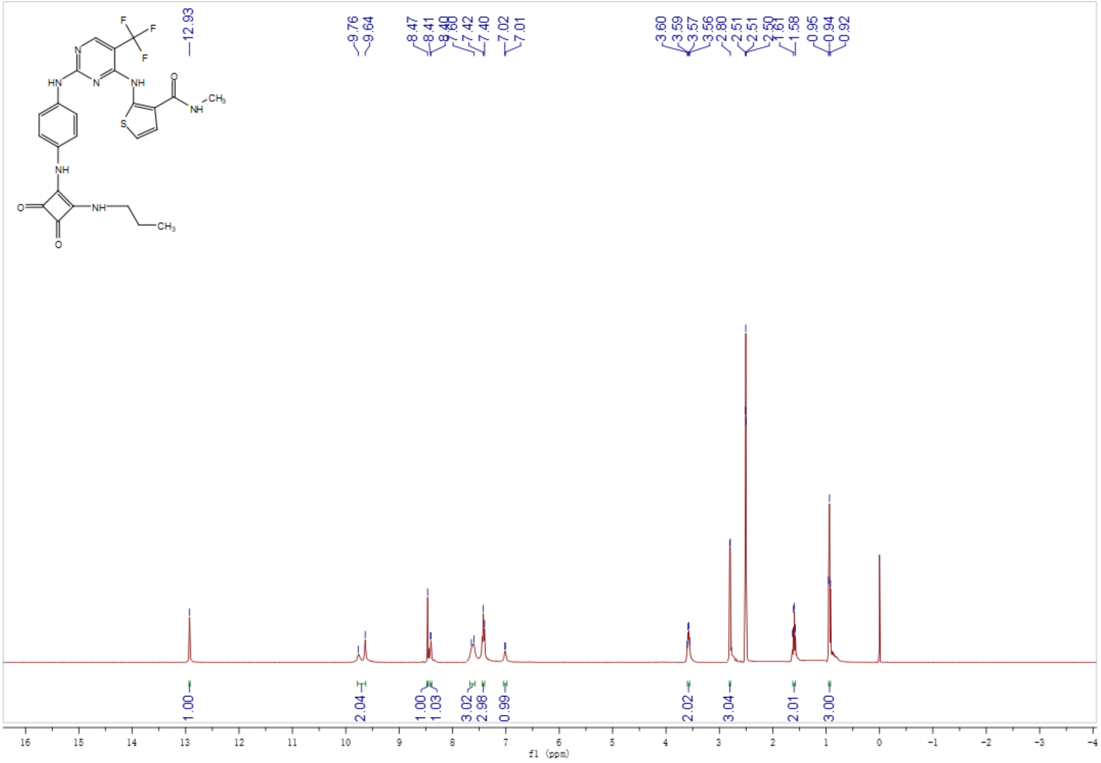

Figure S34. ^{1}H NMR spectrum of compound **6a**

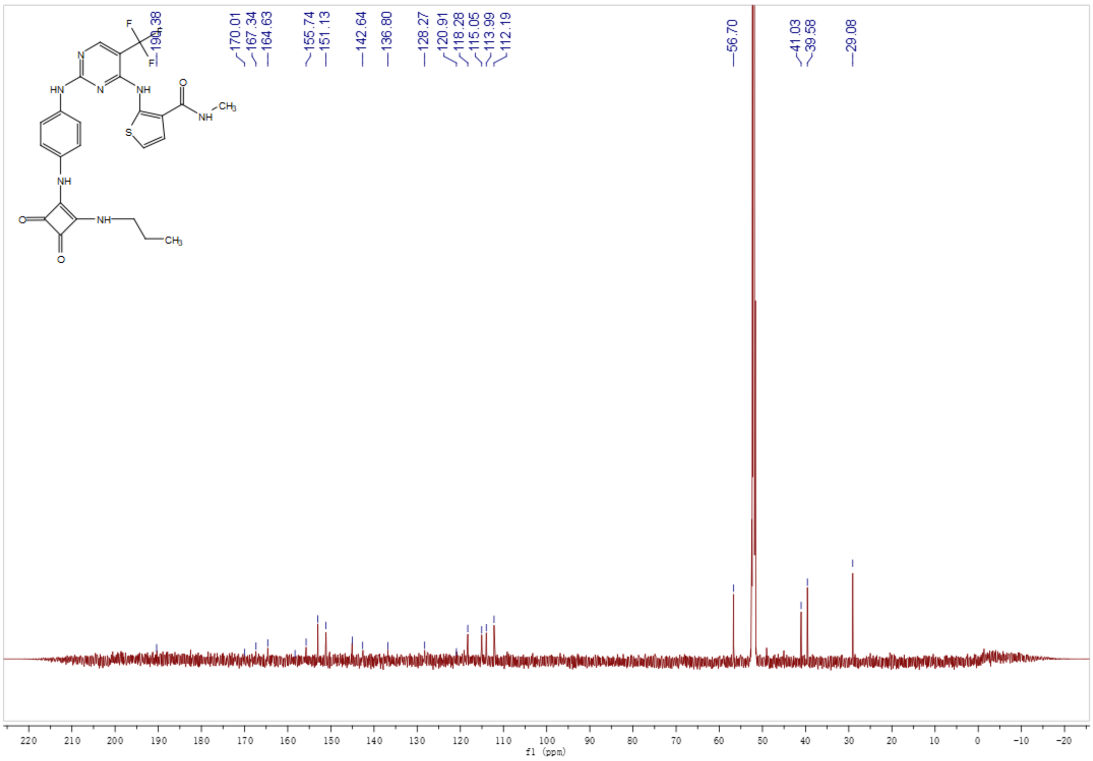

Figure S35. ^{13}C NMR spectrum of compound **6a**

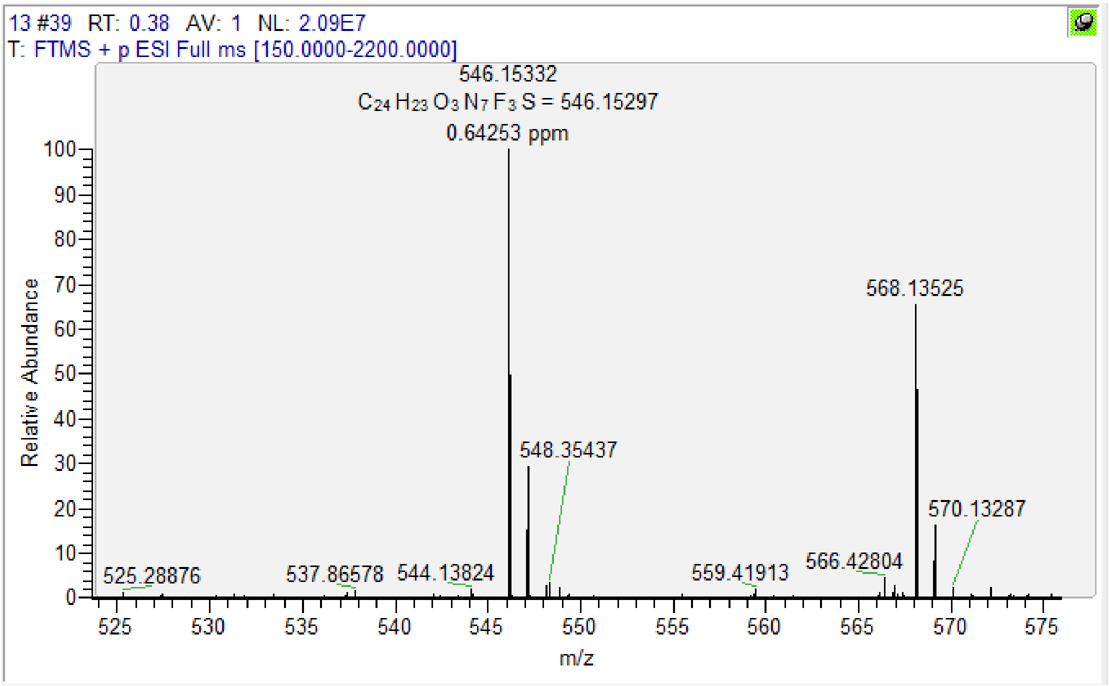

Figure S36. HRMS spectrum of compound **6a**

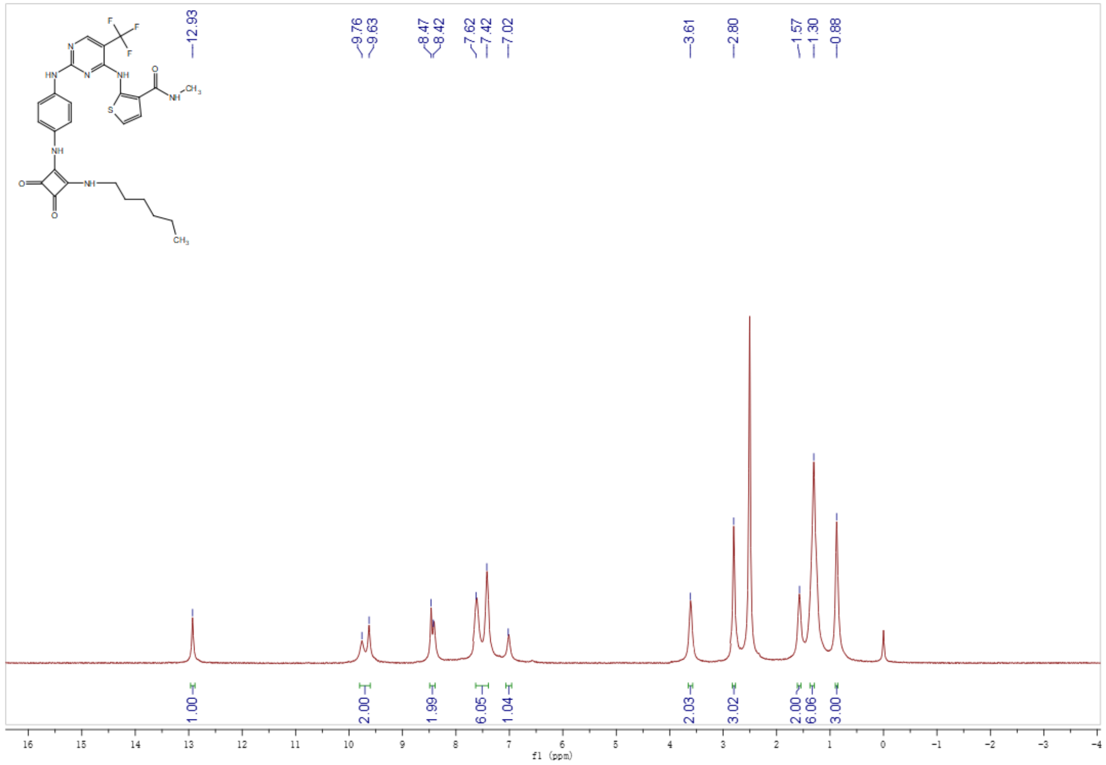

Figure S37. ^{1}H NMR spectrum of compound **6b**

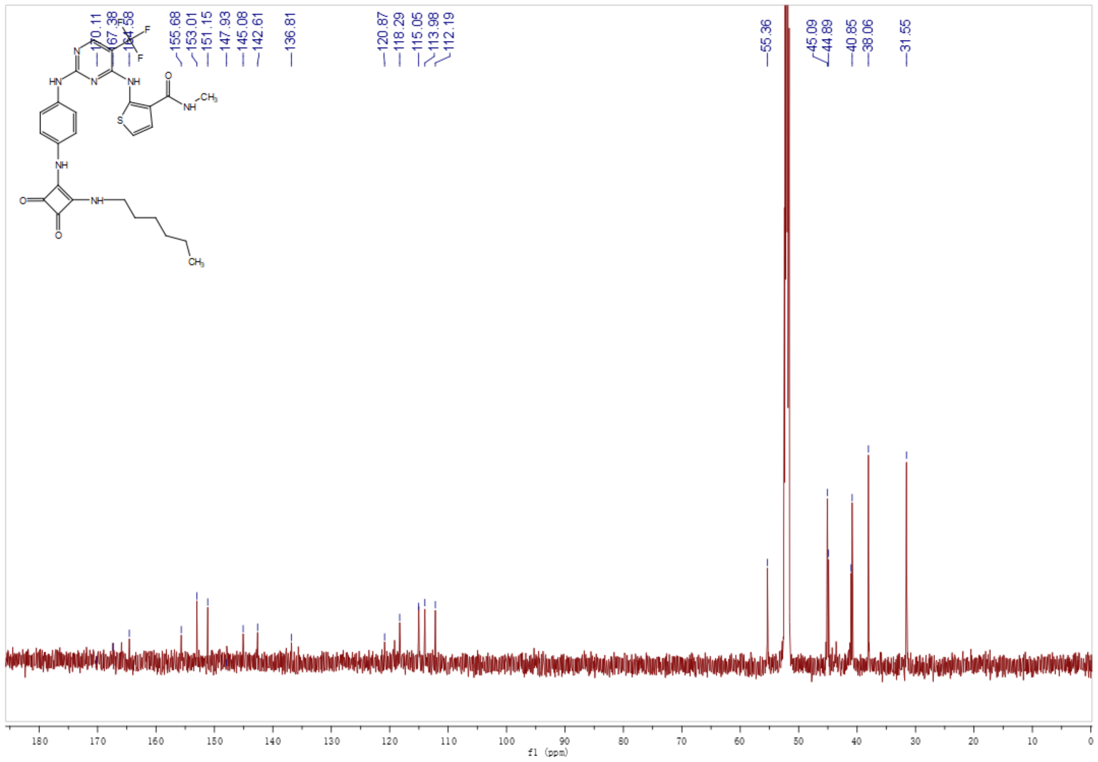

Figure S38. ^{13}C NMR spectrum of compound **6b**

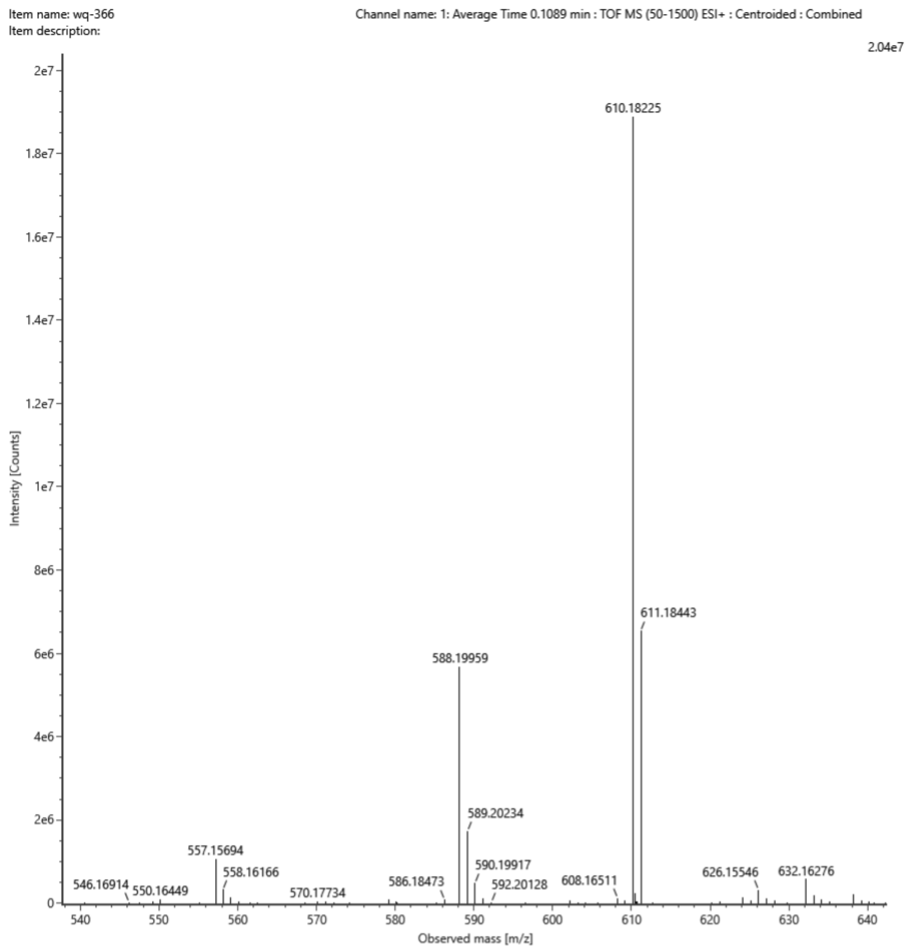

Figure S39. HRMS spectrum of compound **6b**

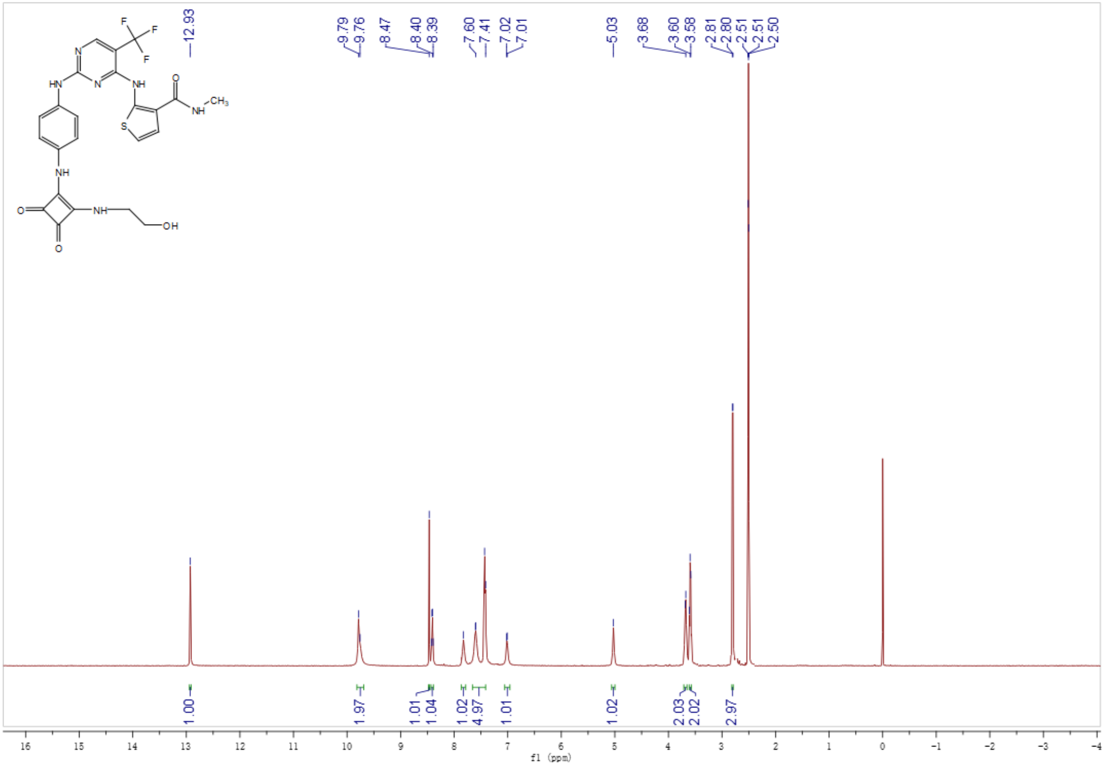

Figure S40. ^{1}H NMR spectrum of compound **6c**

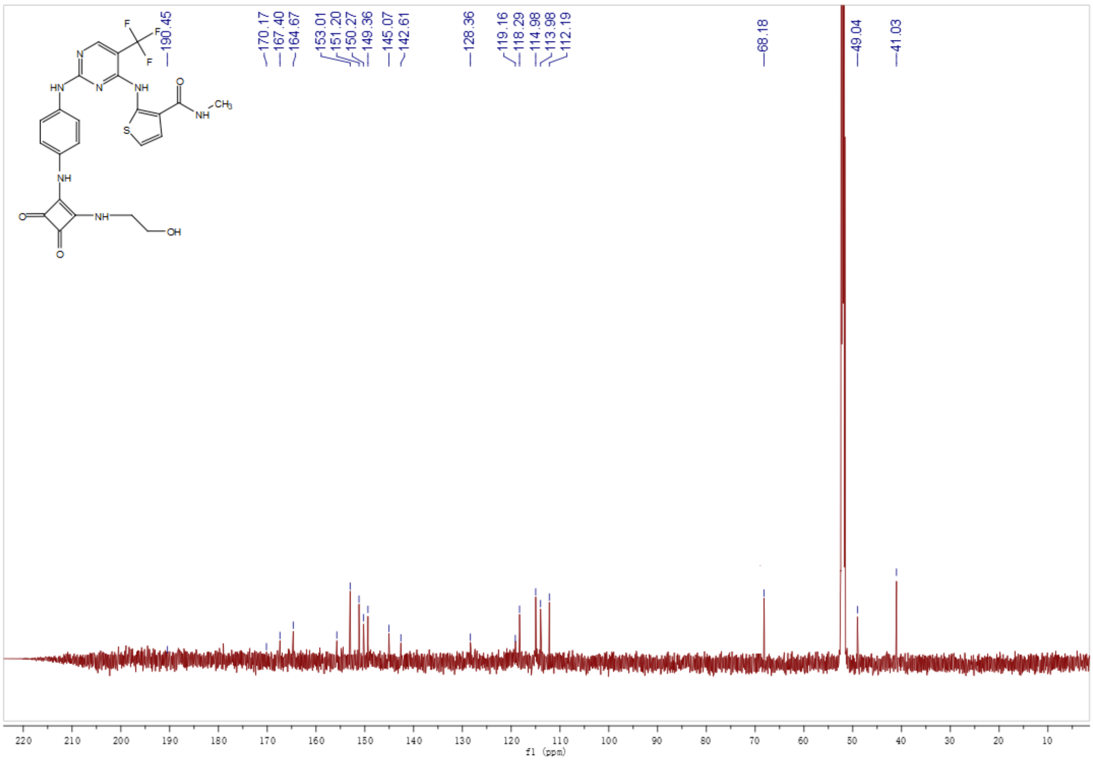

Figure S41. ^{13}C NMR spectrum of compound **6c**

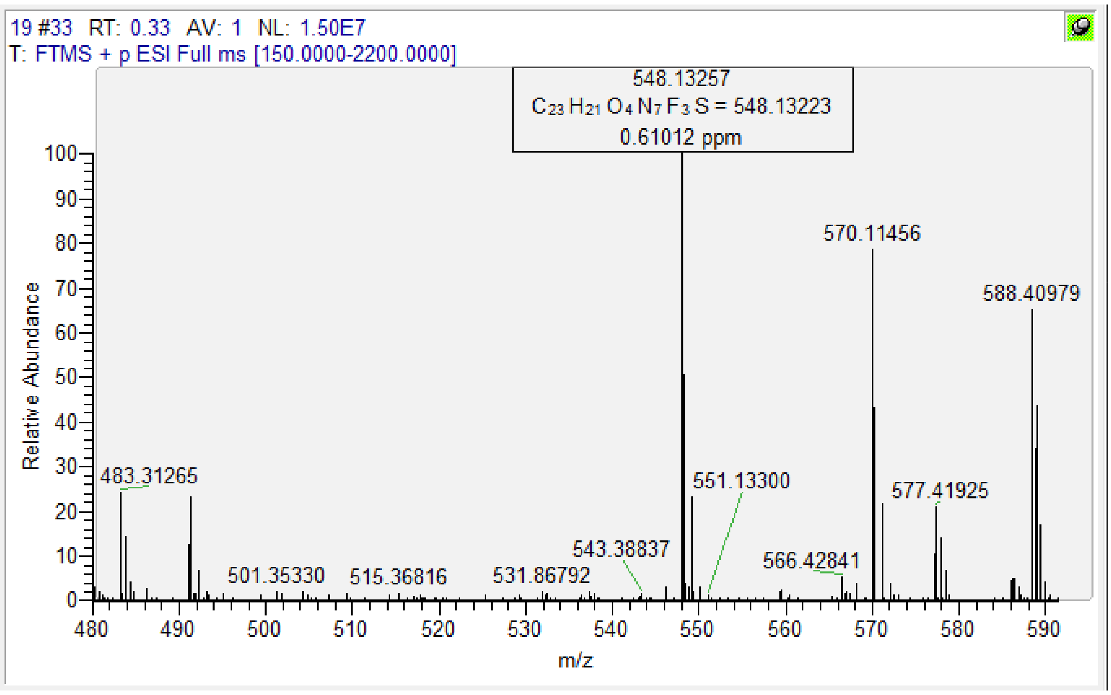

Figure S42. HRMS spectrum of compound **6c**

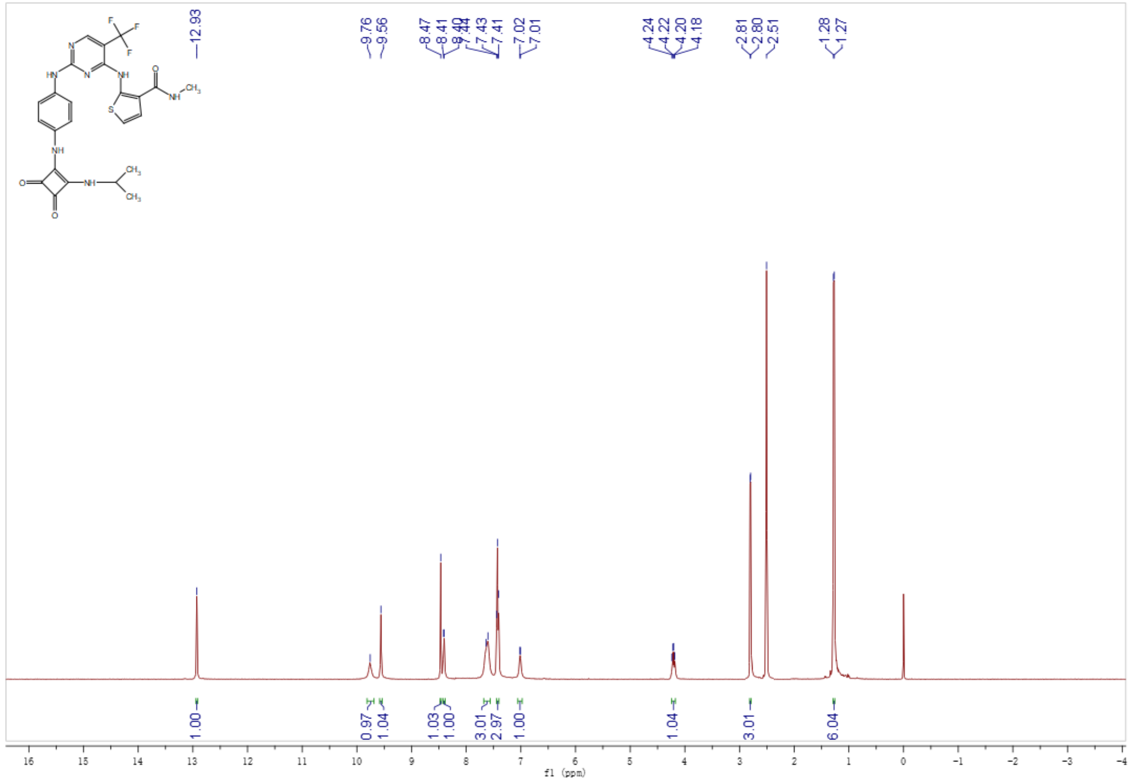

Figure S43. ^{1}H NMR spectrum of compound **6d**

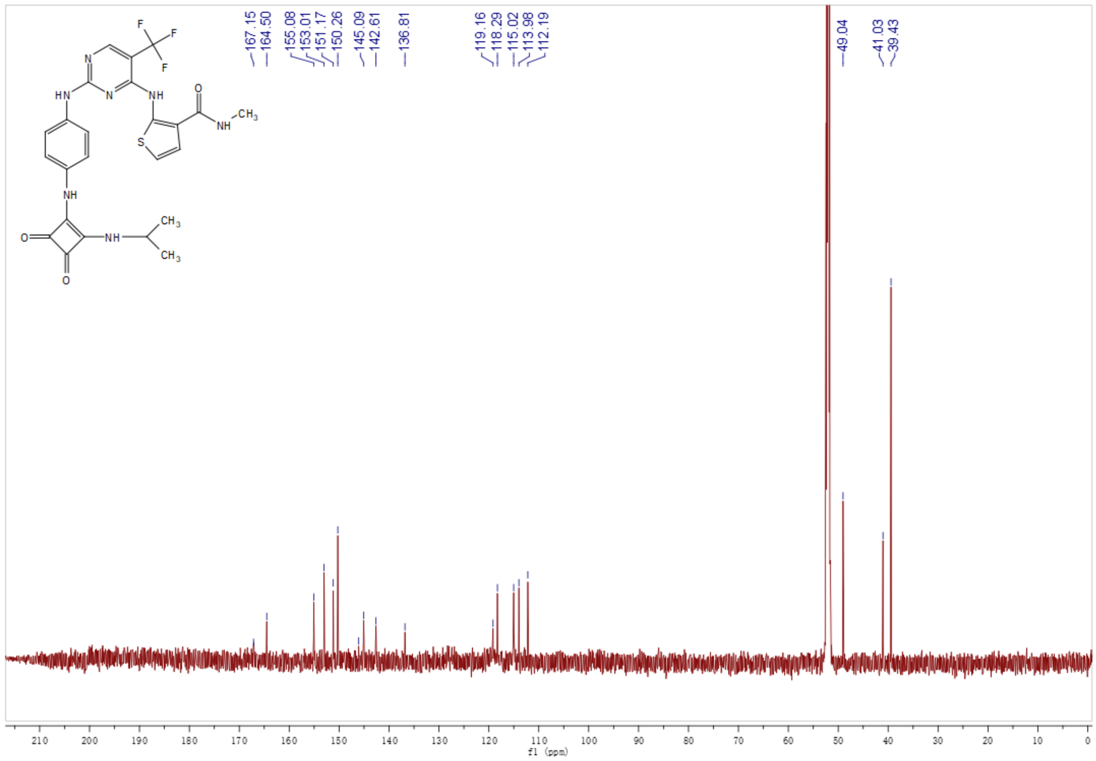

Figure S44. ^{13}C NMR spectrum of compound **6d**

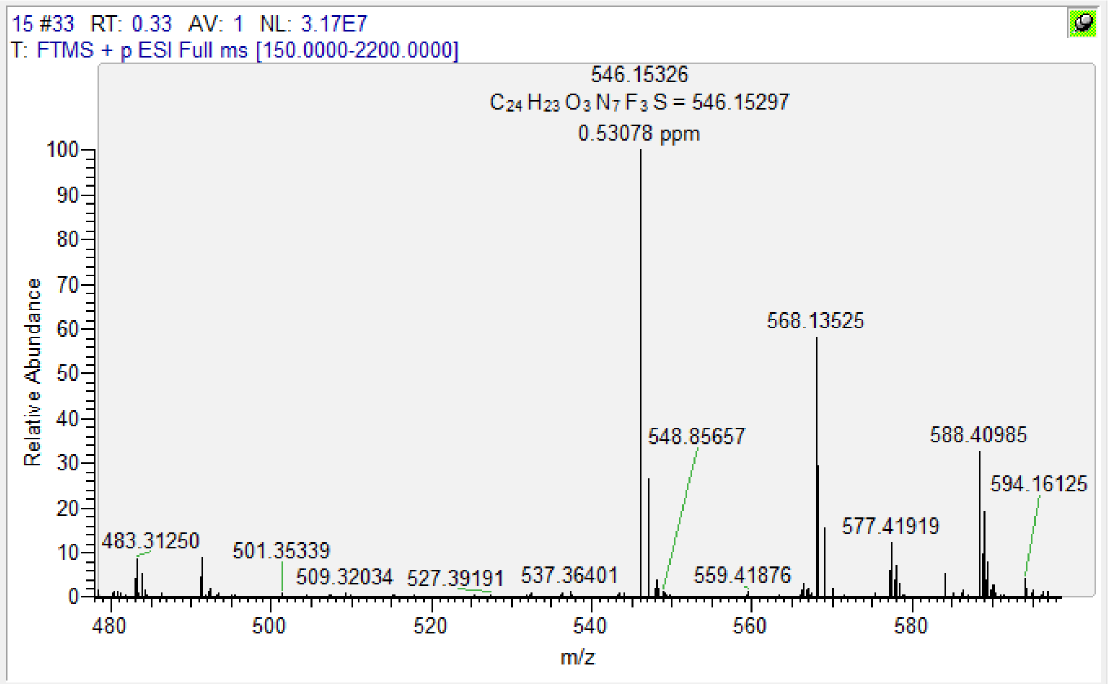

Figure S45. HRMS spectrum of compound **6d**

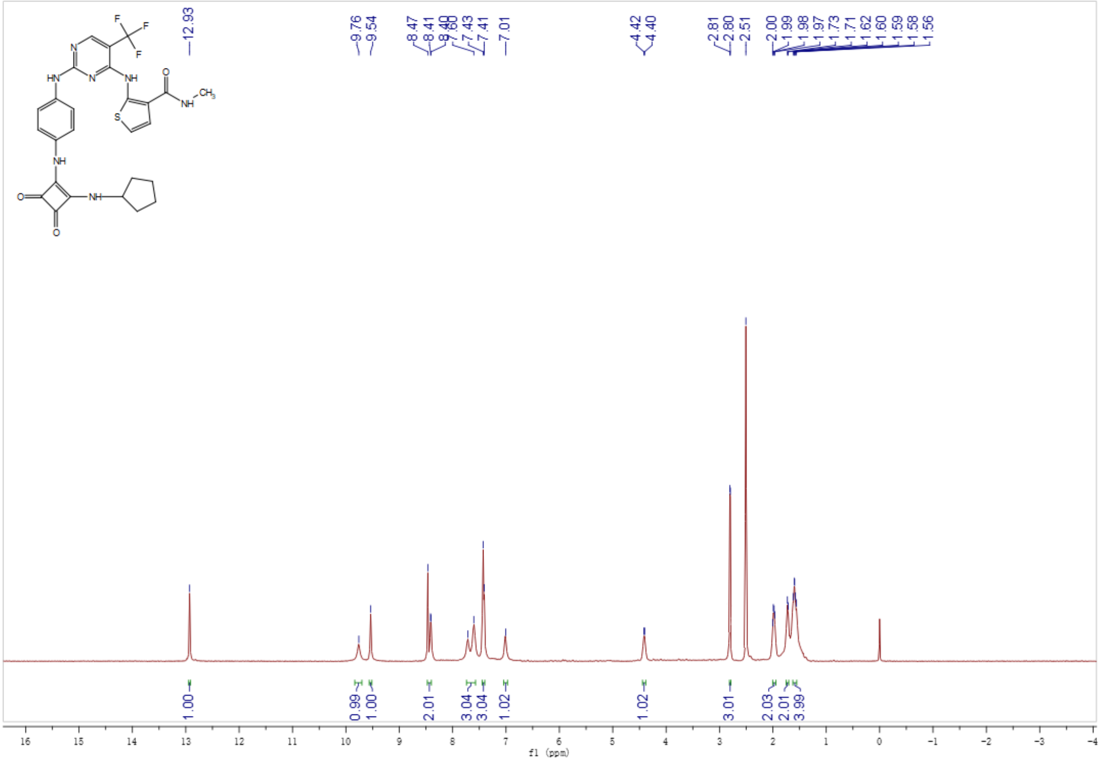

Figure S46. ^{1}H NMR spectrum of compound **6e**

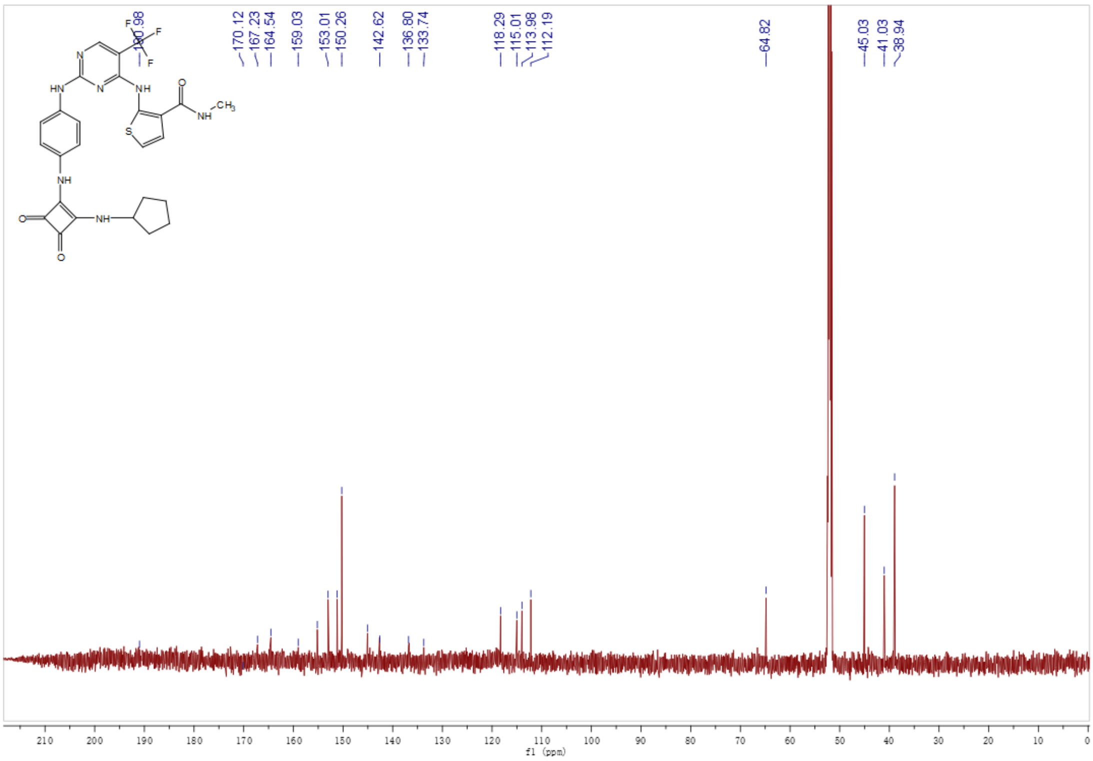

Figure S47. ^{13}C NMR spectrum of compound **6e**

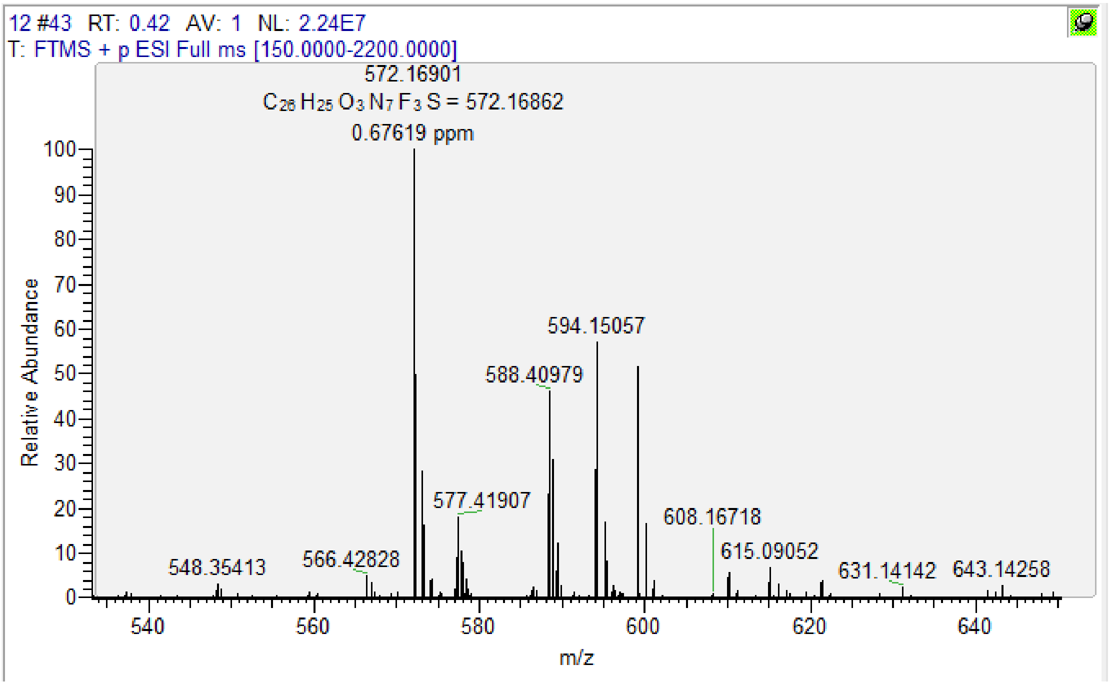

Figure S48. HRMS spectrum of compound **6e**

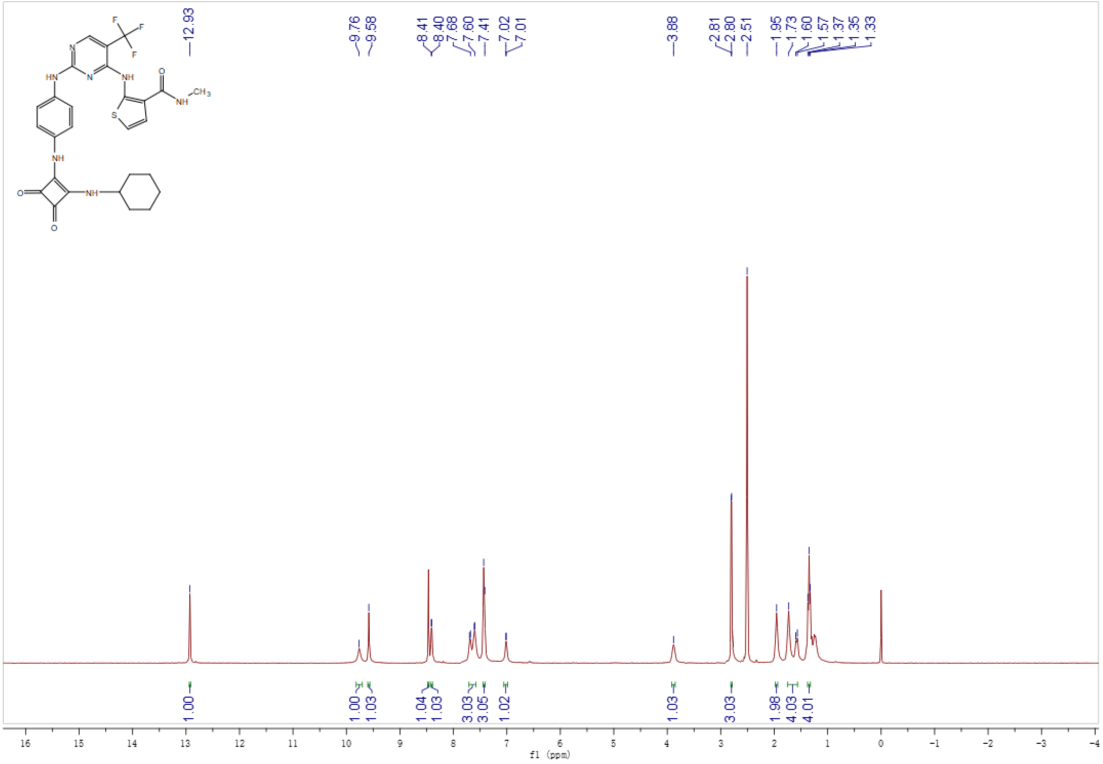

Figure S49. ^{1}H NMR spectrum of compound **6f**

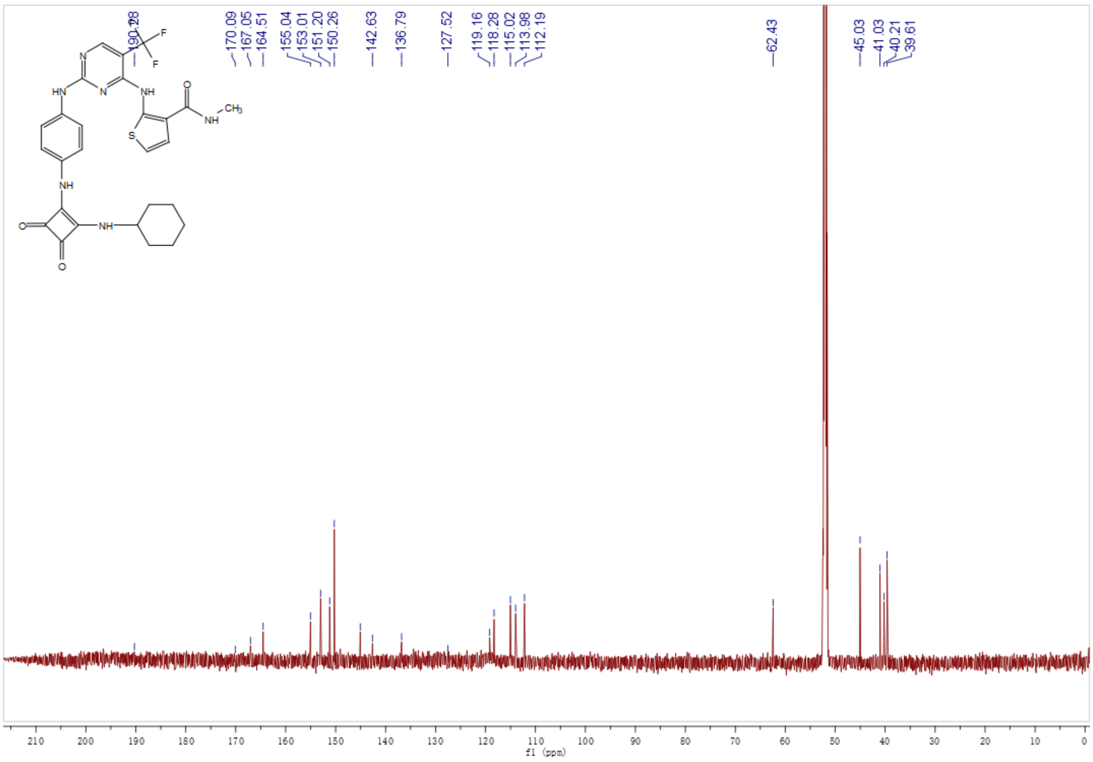

Figure S50. ^{13}C NMR spectrum of compound **6f**

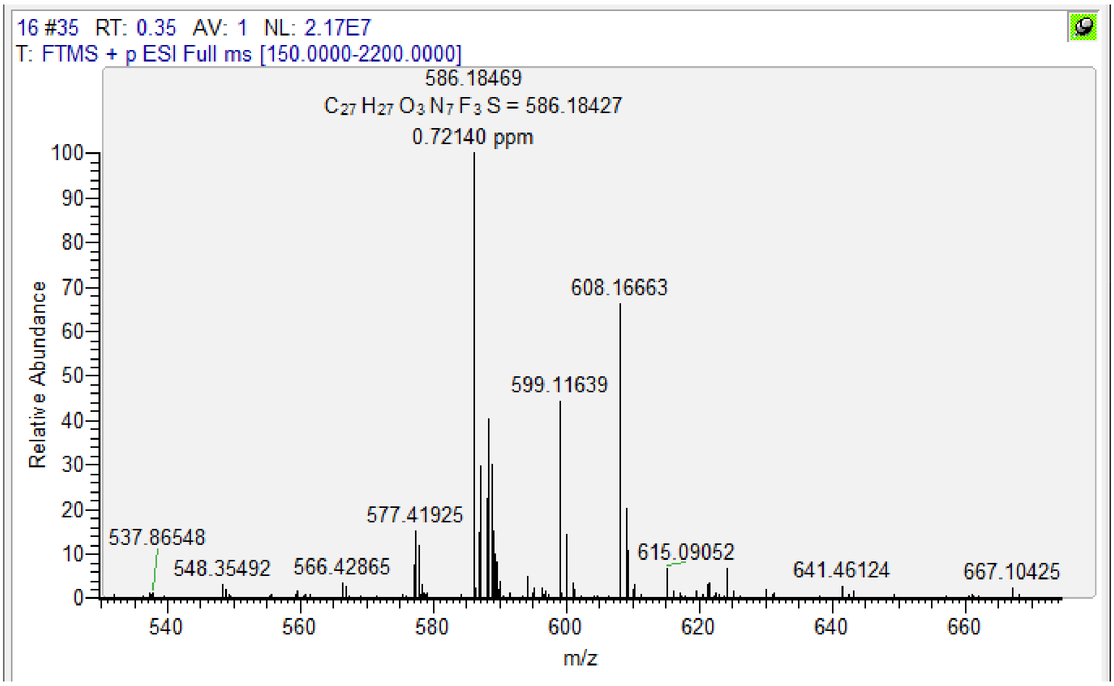

Figure S51. HRMS spectrum of compound **6f**

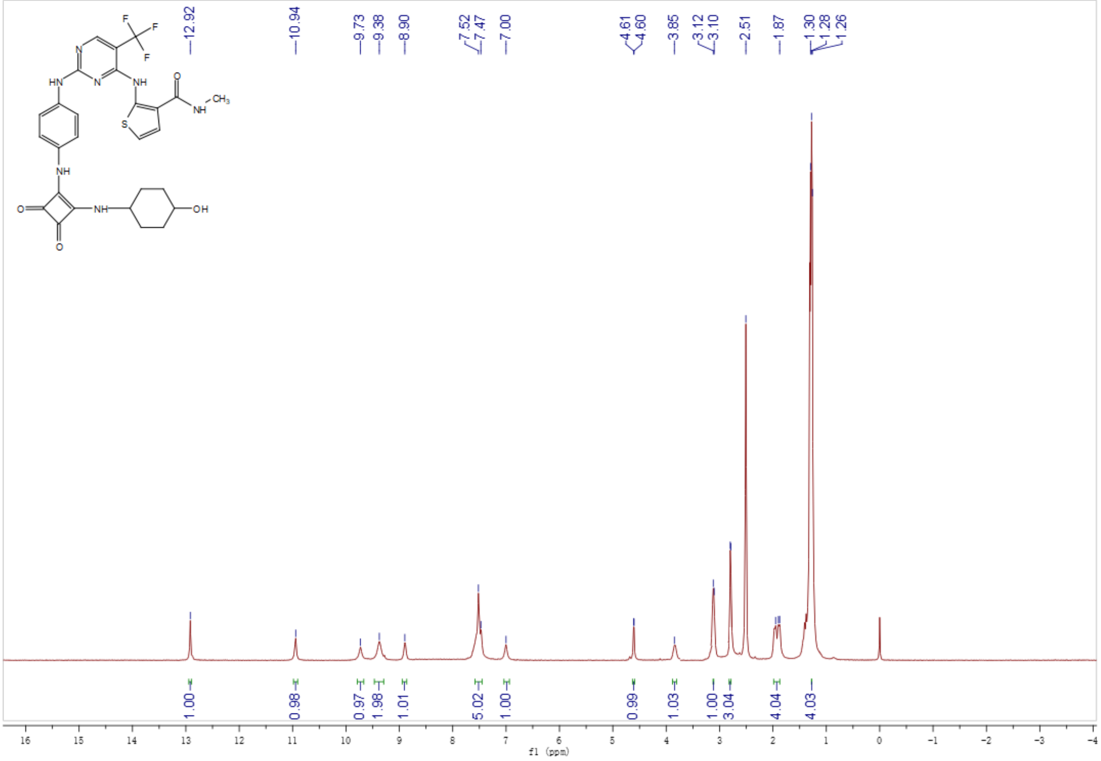

Figure S52. ^{1}H NMR spectrum of compound **6g**

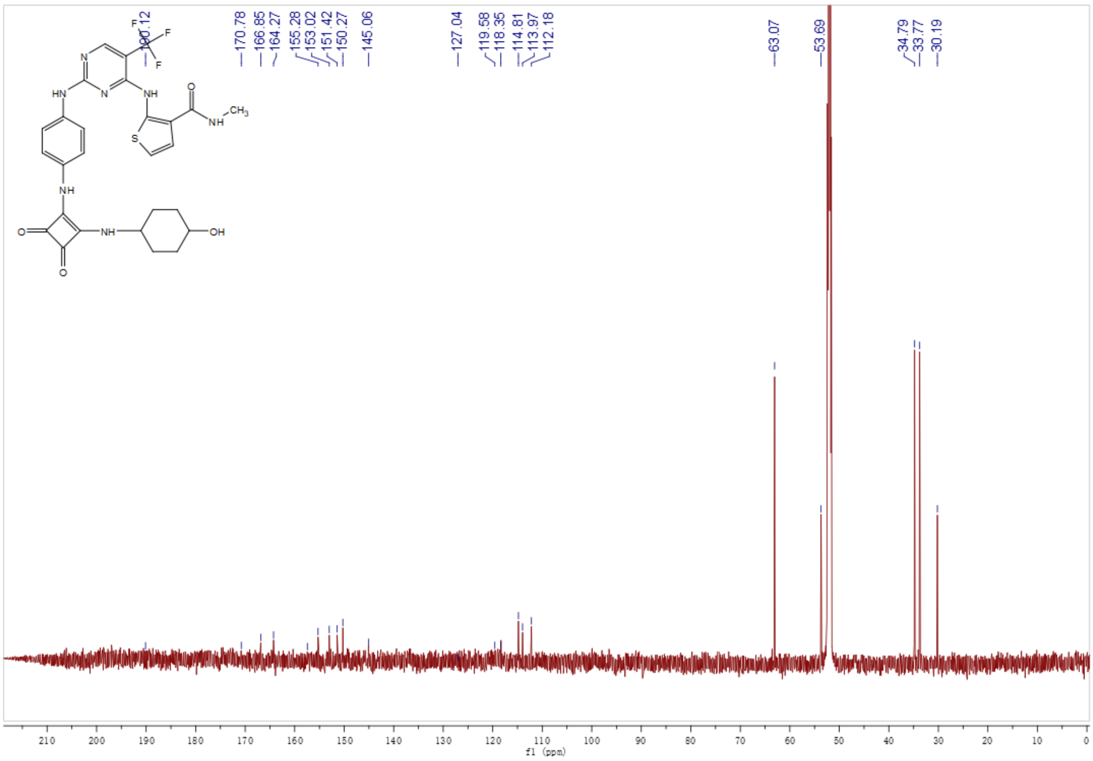

Figure S53. ^{13}C NMR spectrum of compound **6g**

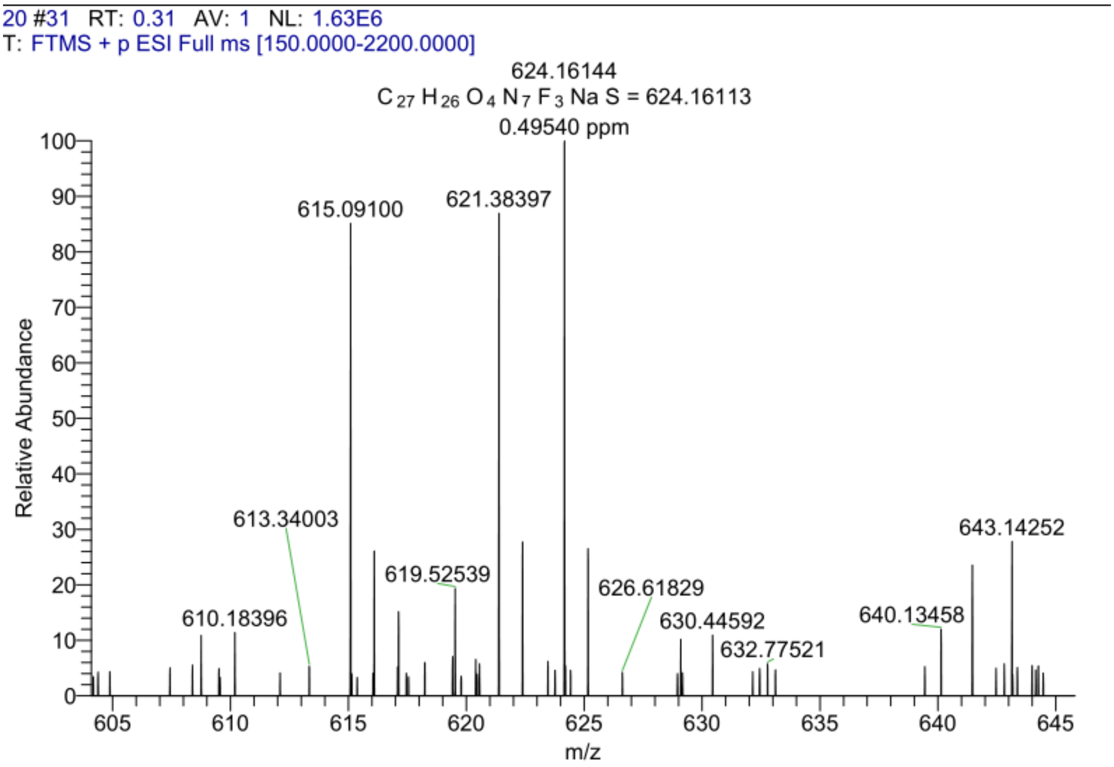

Figure S54. HRMS spectrum of compound **6g**

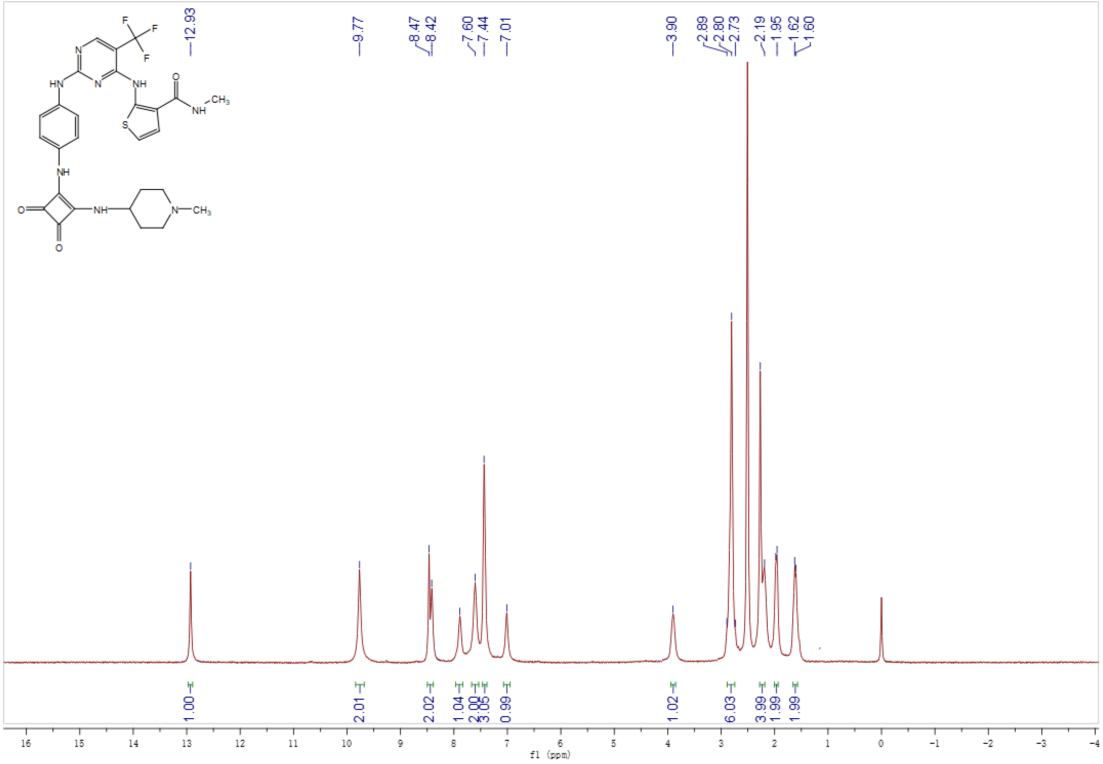

Figure S55. ^{1}H NMR spectrum of compound **6h**

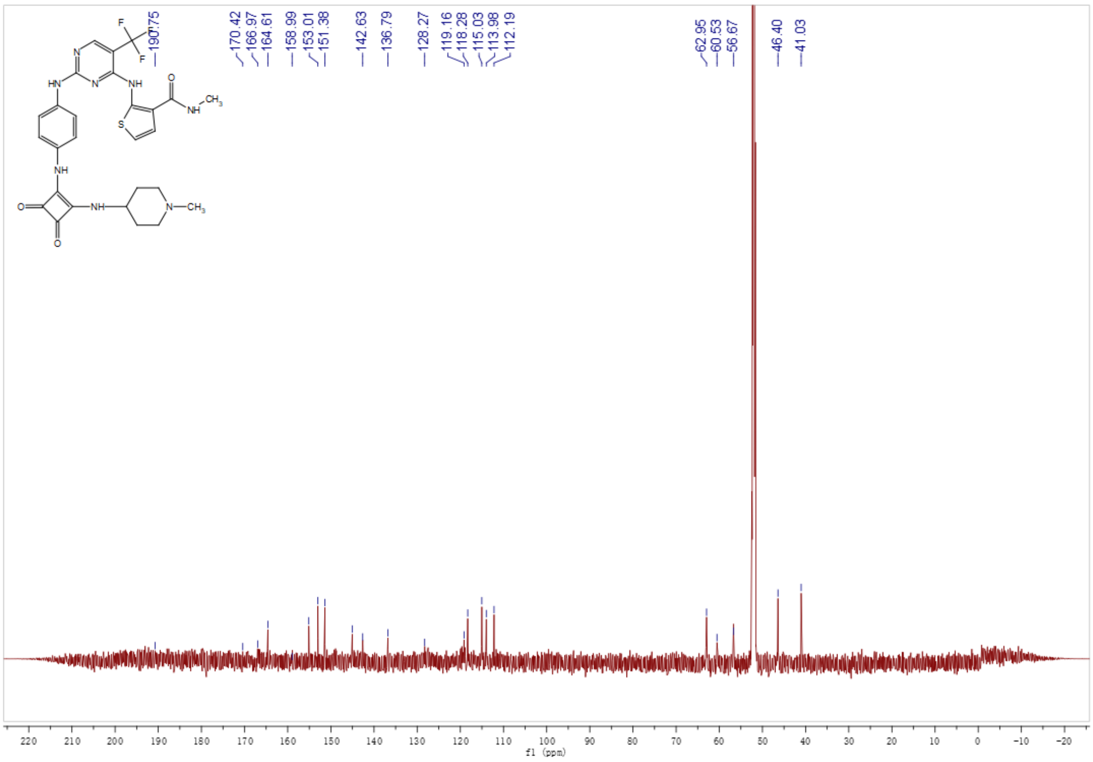

Figure S56. ^{13}C NMR spectrum of compound **6h**

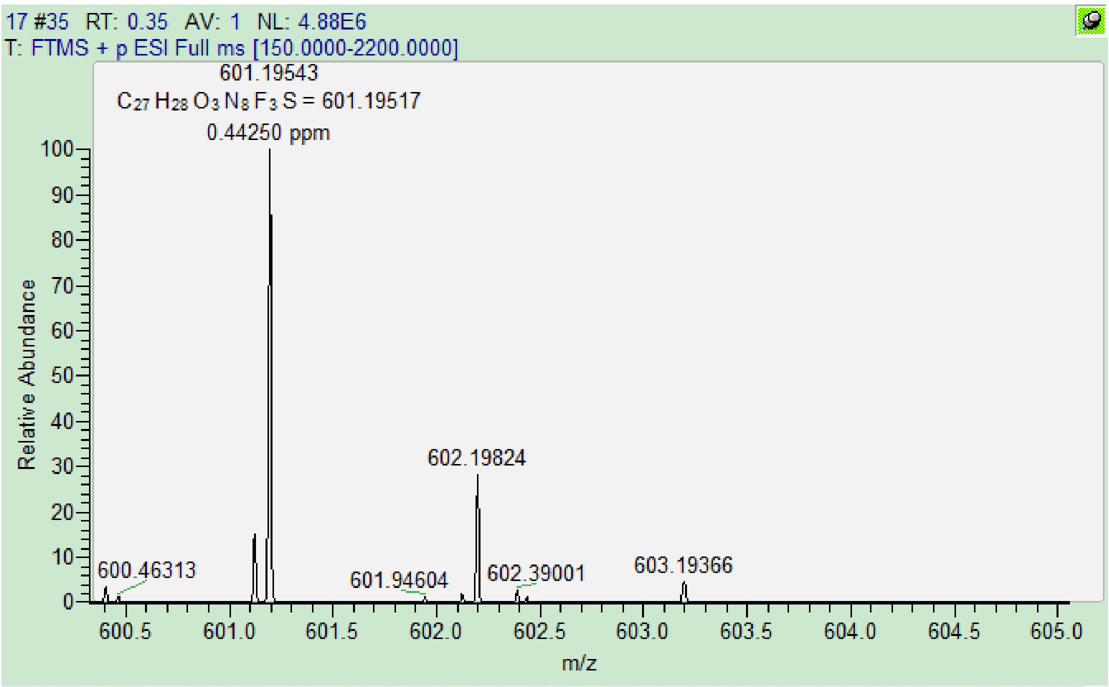

Figure S57. HRMS spectrum of compound **6h**

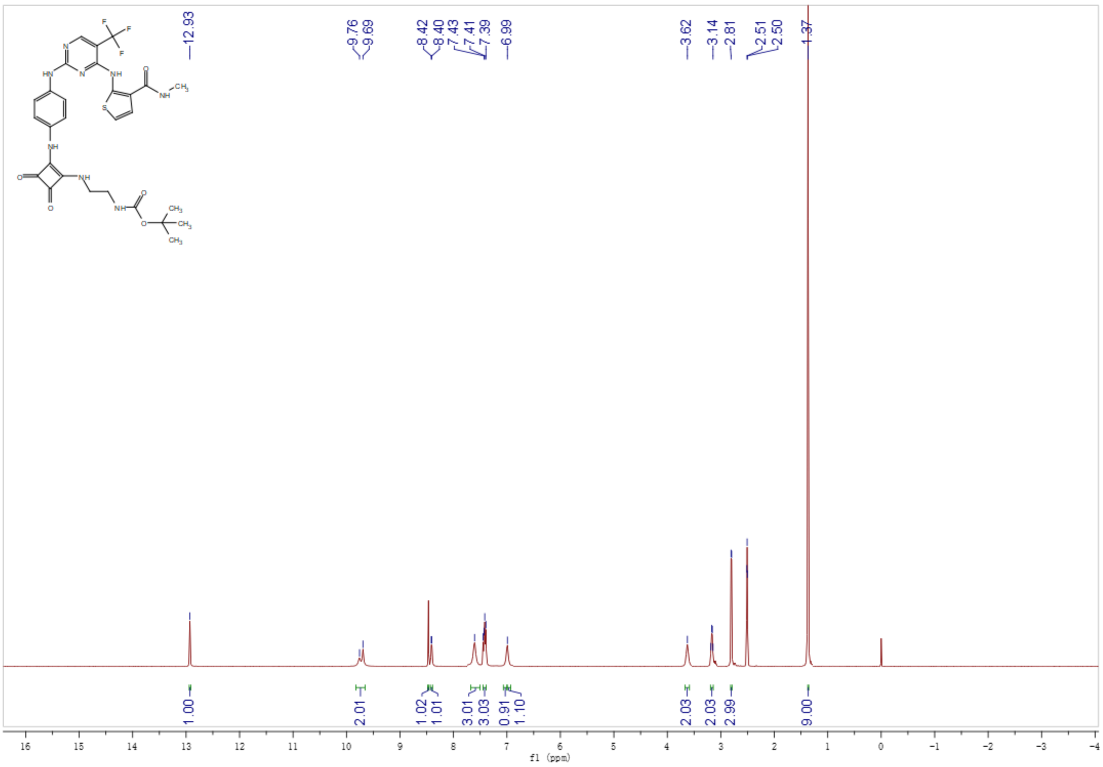

Figure S58. ^{1}H NMR spectrum of compound **6i**

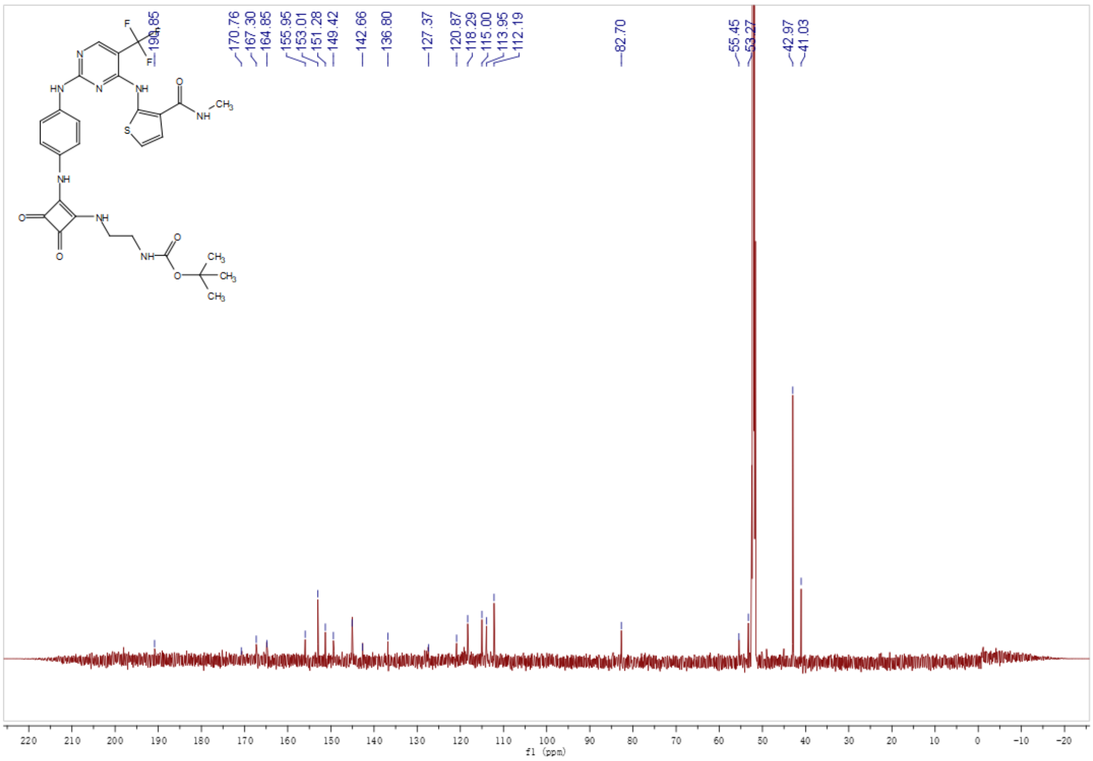

Figure S59. ^{13}C NMR spectrum of compound **6i**

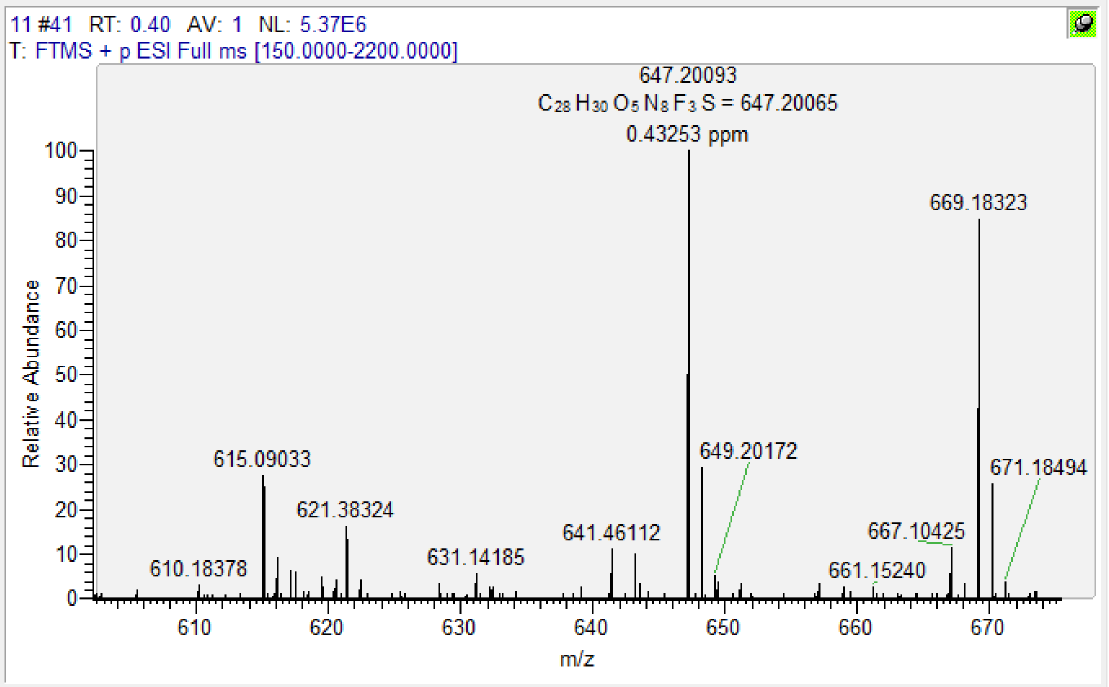

Figure S60. HRMS spectrum of compound **6i**

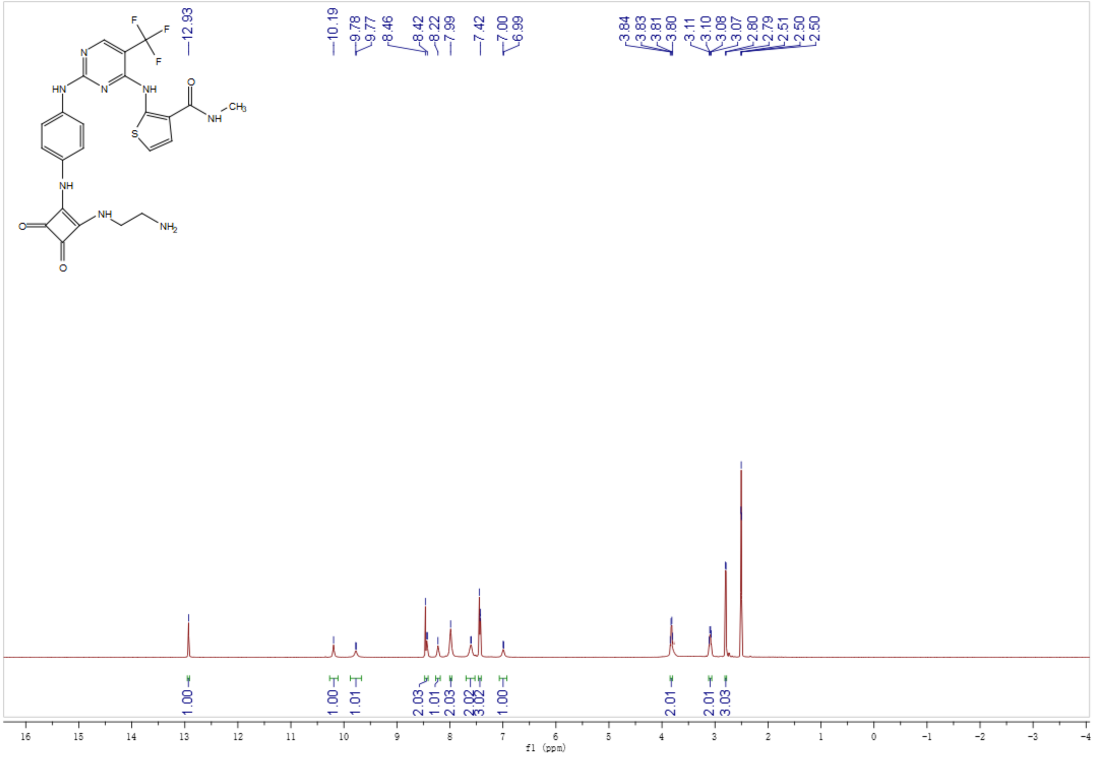

Figure S61. ^{1}H NMR spectrum of compound **7**

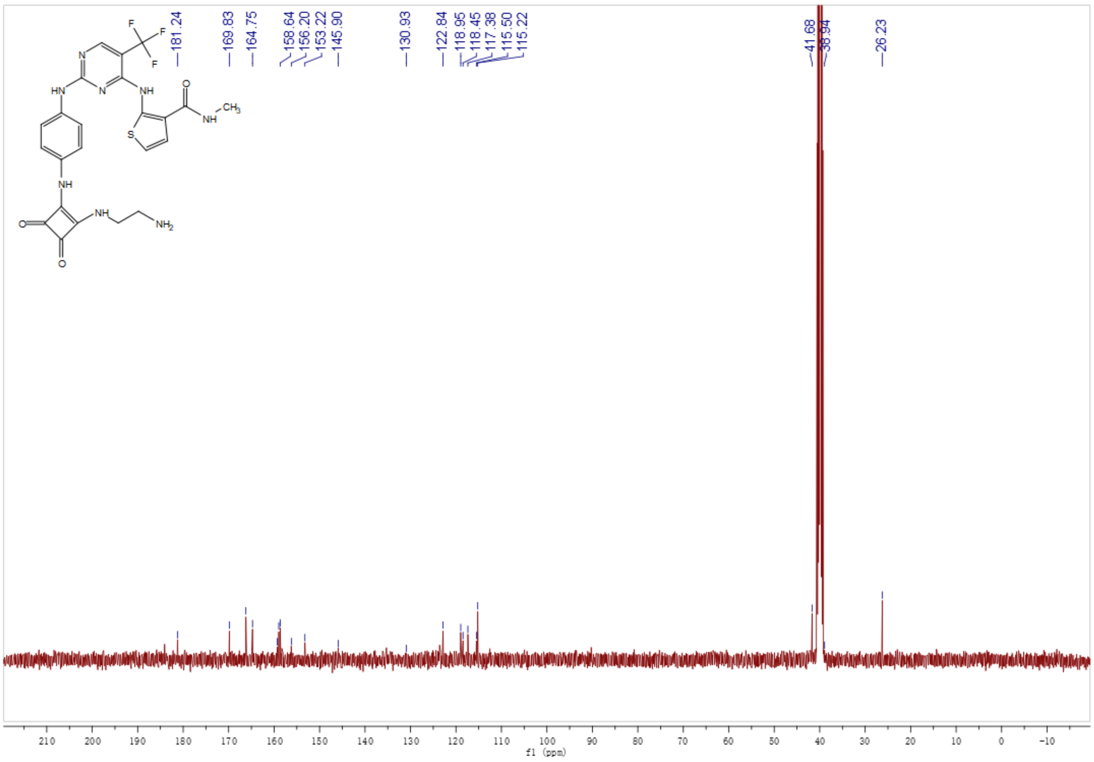

Figure S62. ^{13}C NMR spectrum of compound **7**

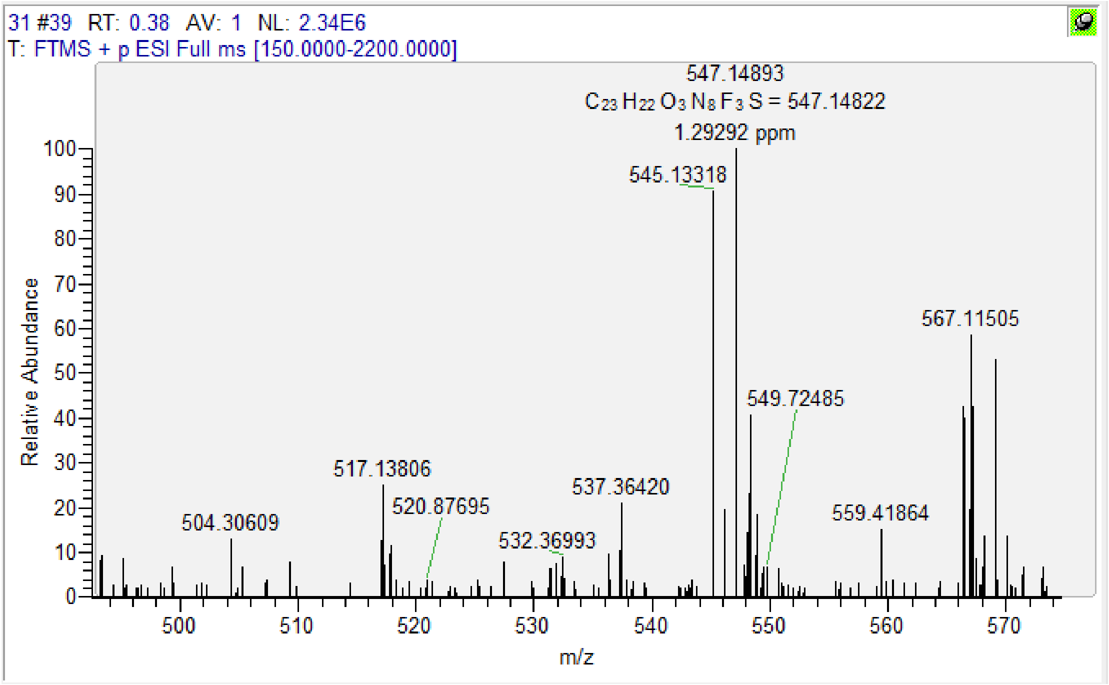

Figure S63. HRMS spectrum of compound **7**

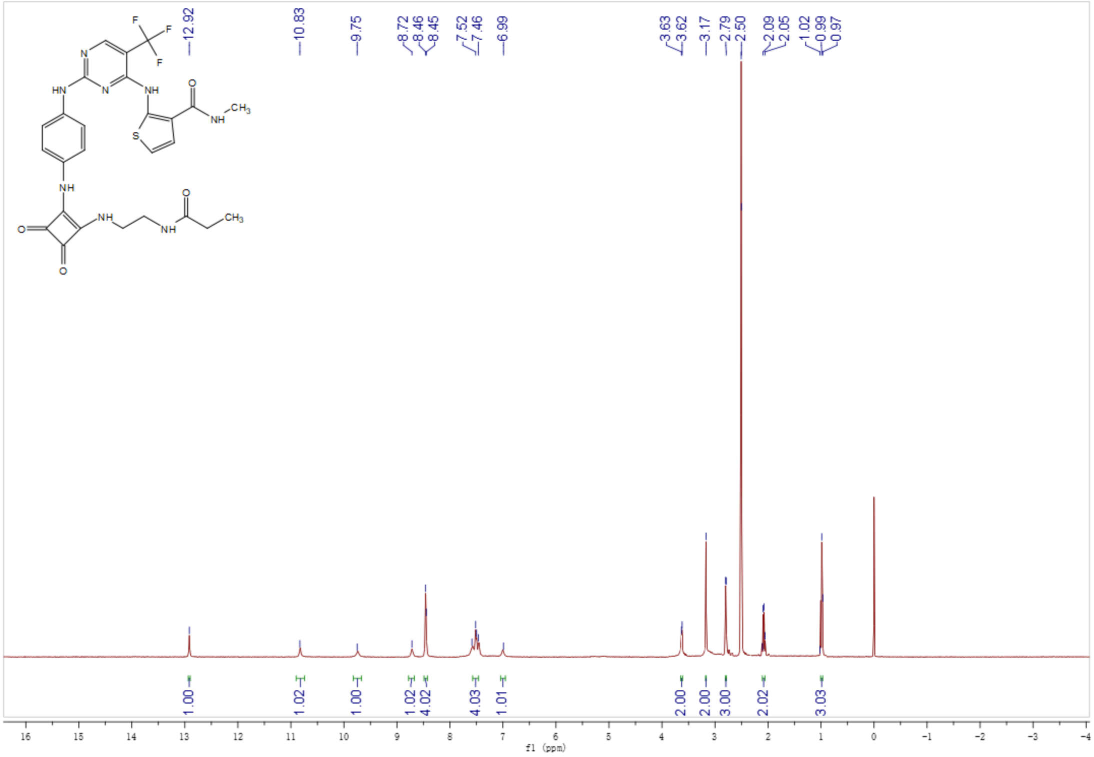

Figure S64. ^{1}H NMR spectrum of compound **8a**

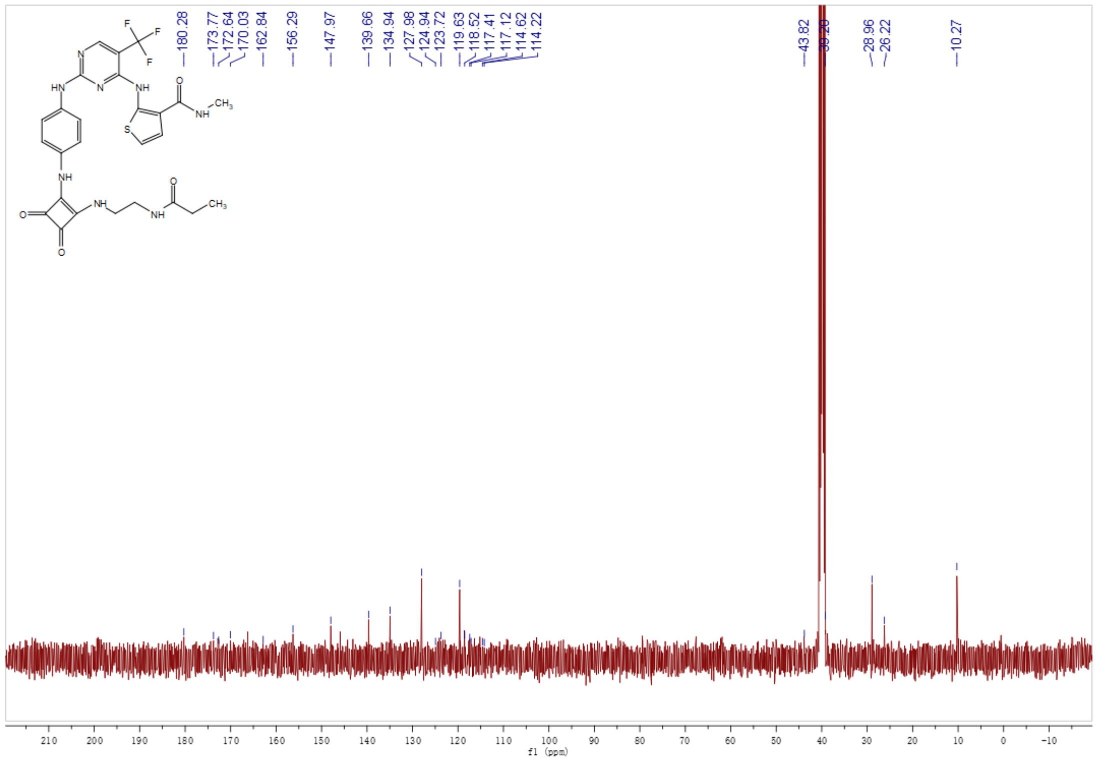

Figure S65. ^{13}C NMR spectrum of compound **8a**

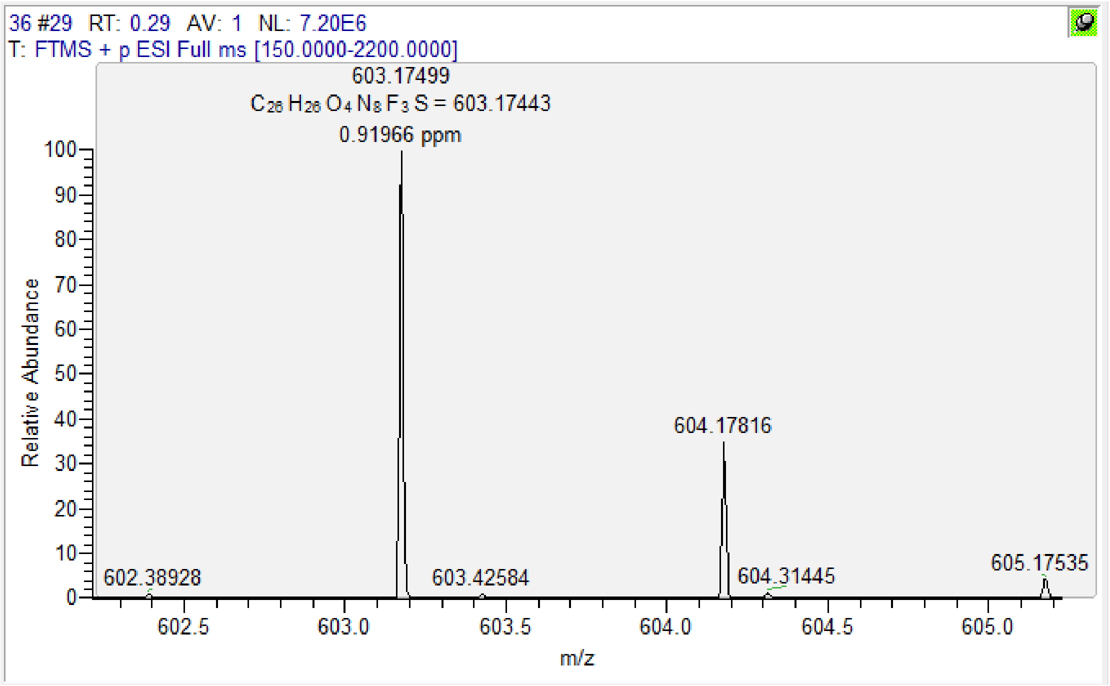

Figure S66. HRMS spectrum of compound **8a**

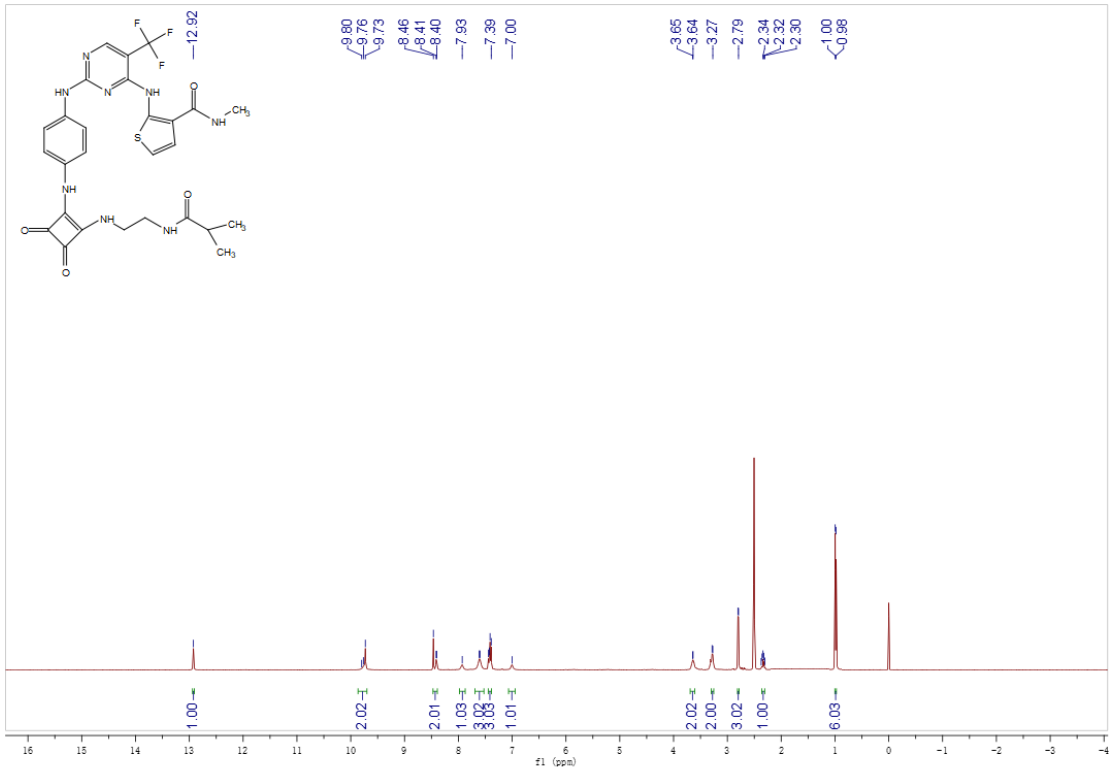

Figure S67. ^{1}H NMR spectrum of compound **8b**

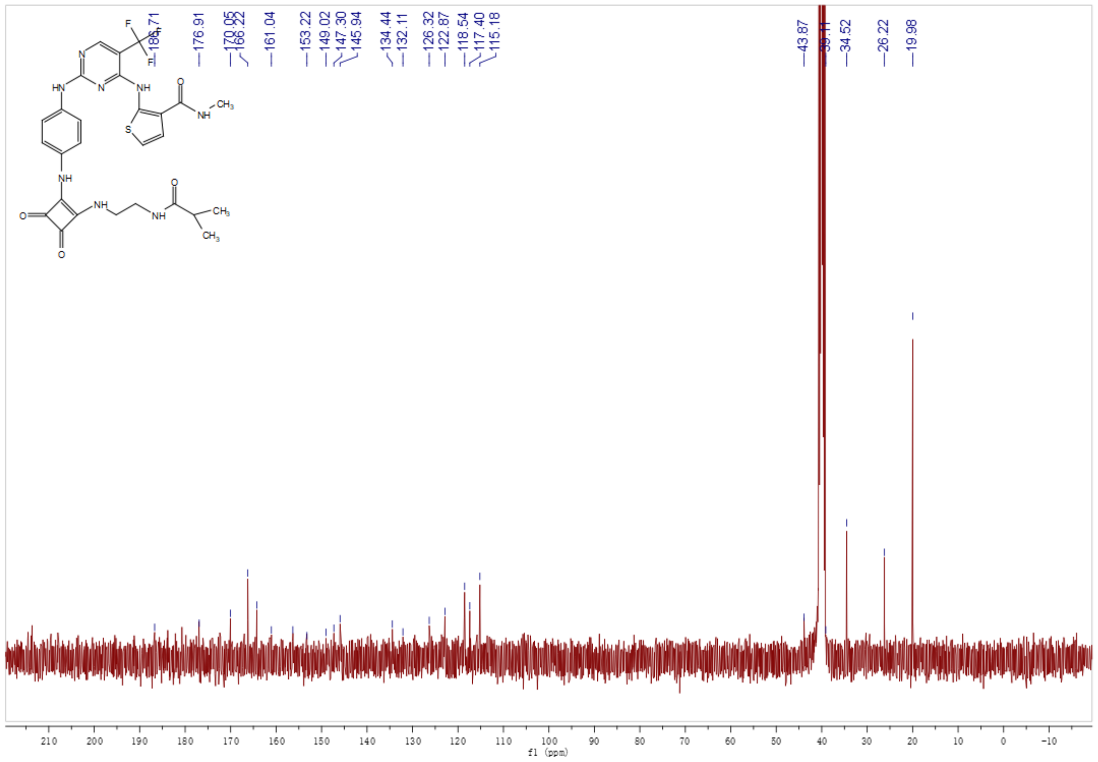

Figure S68. ^{13}C NMR spectrum of compound **8b**

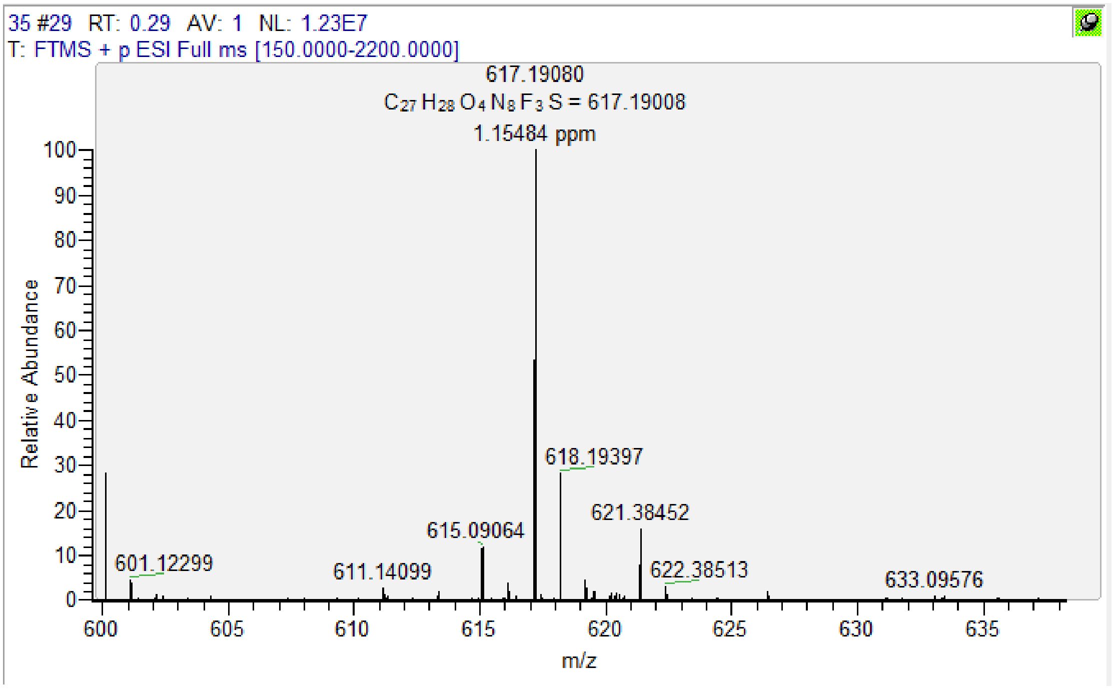

Figure S69. HRMS spectrum of compound **8b**

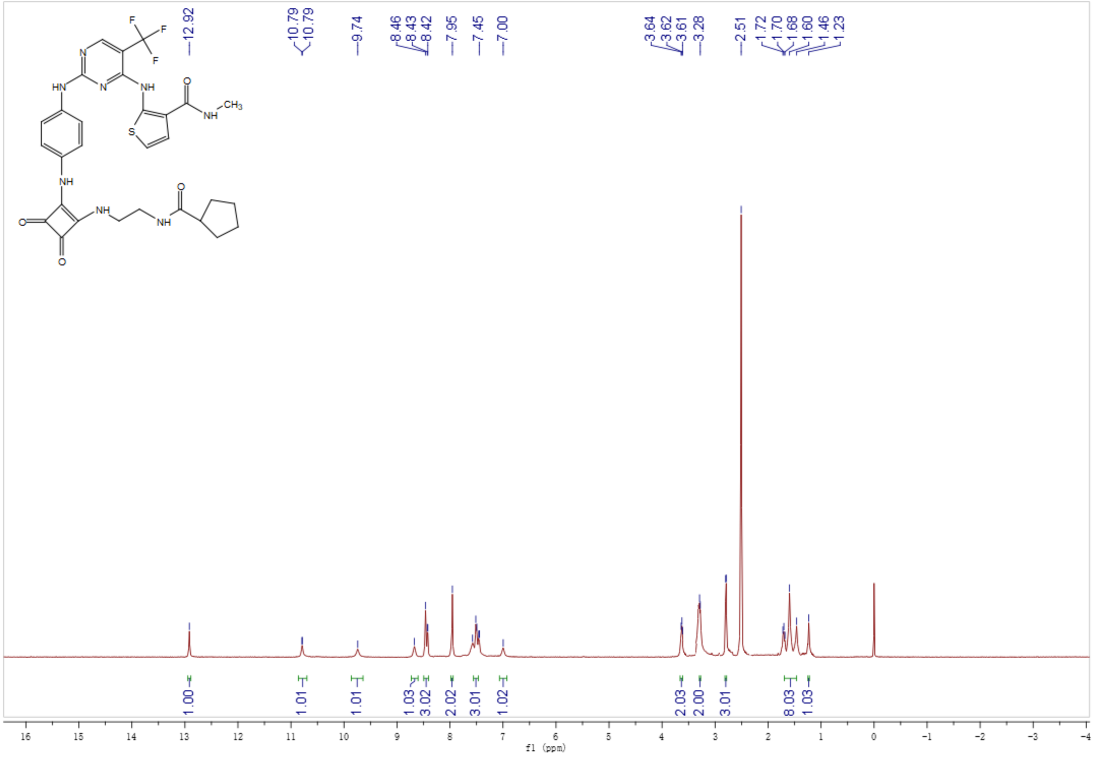

Figure S70. ^{1}H NMR spectrum of compound **8c**

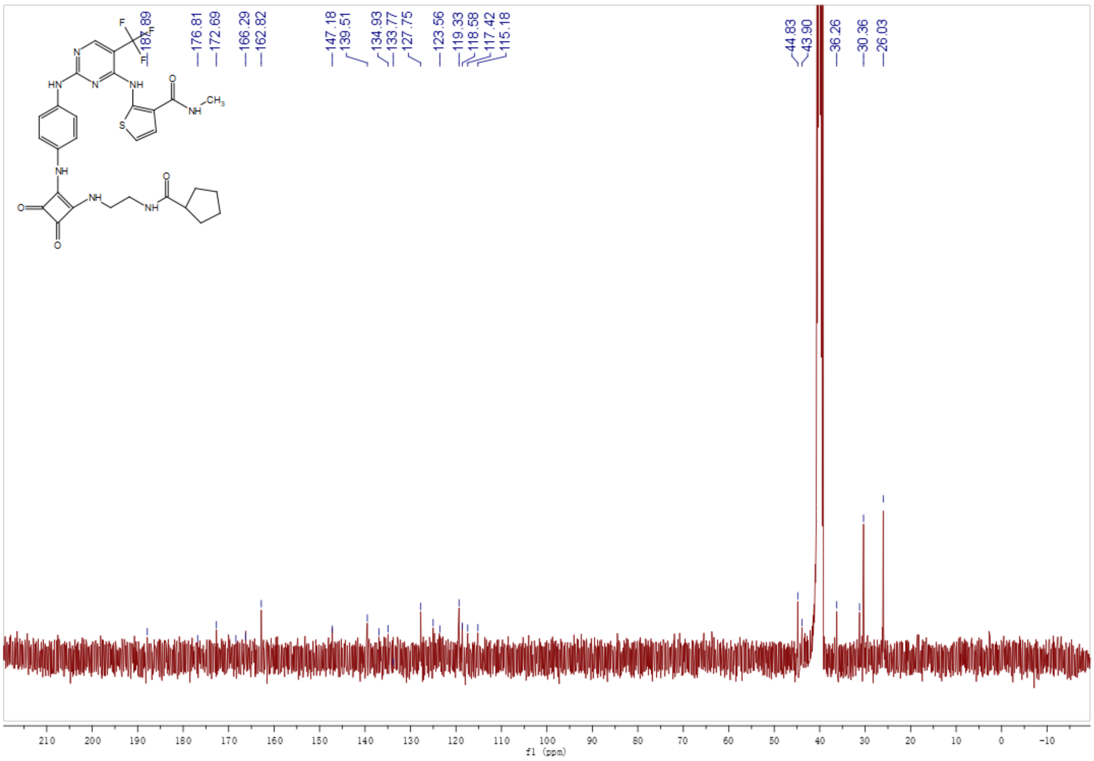

Figure S71. ^{13}C NMR spectrum of compound **8c**

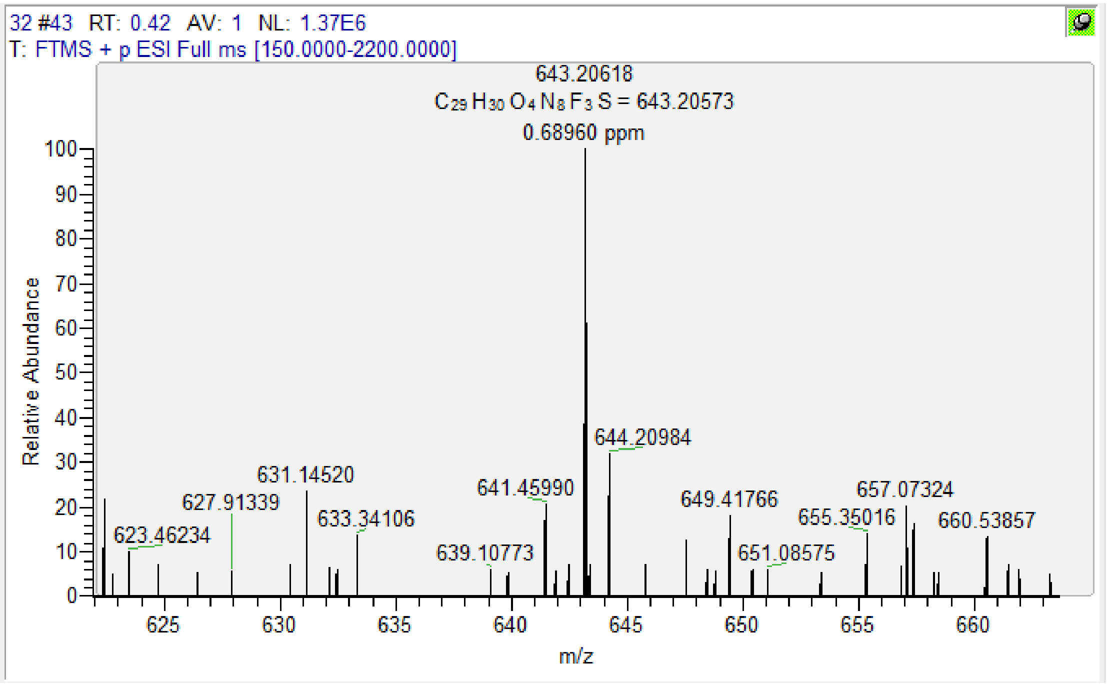

Figure S72. HRMS spectrum of compound **8c**

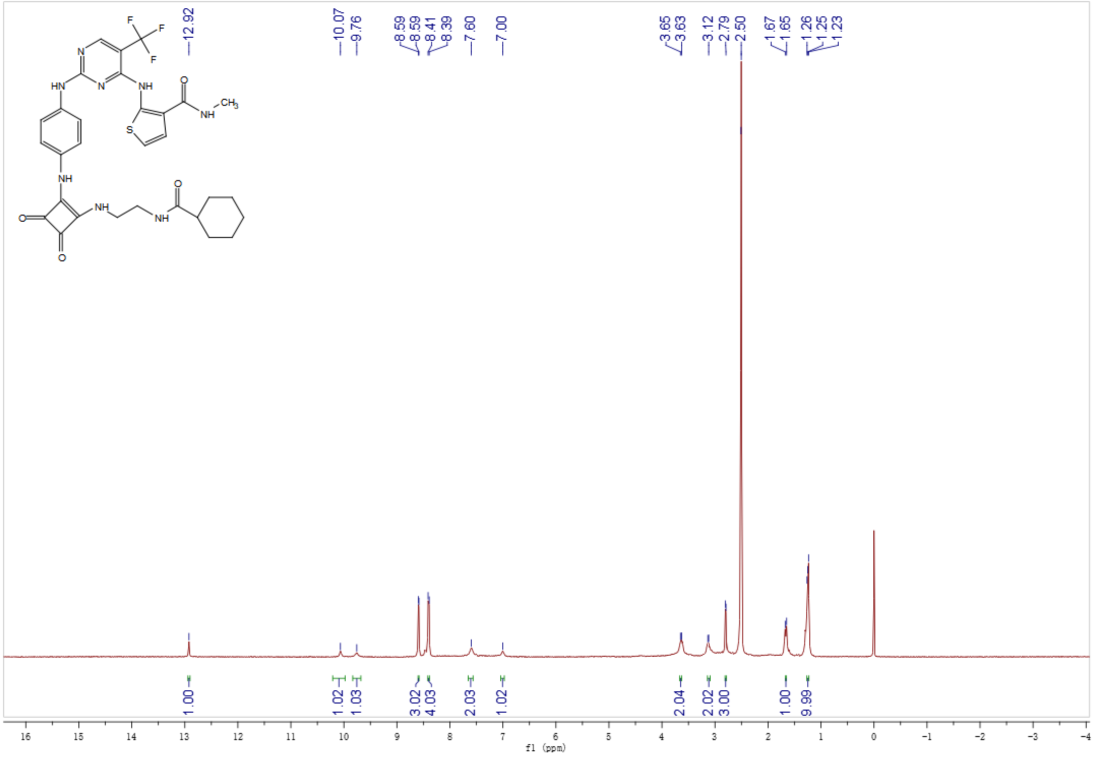

Figure S73. ^{1}H NMR spectrum of compound **8d**

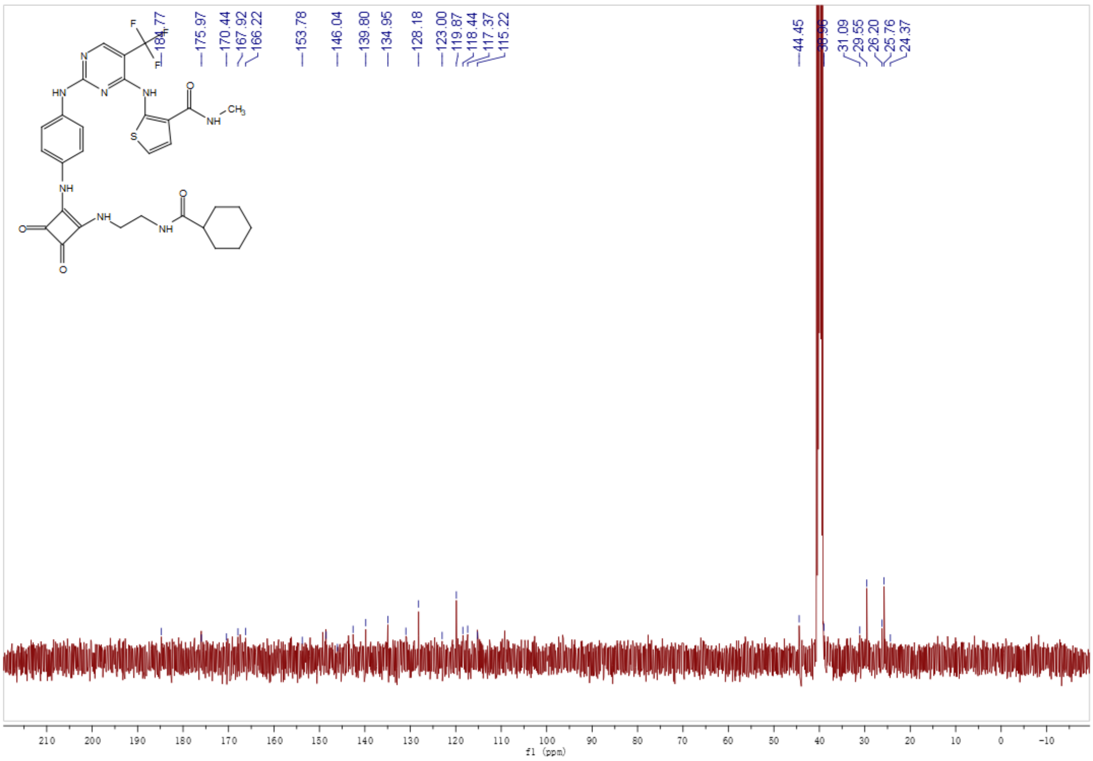

Figure S74. ^{13}C NMR spectrum of compound **8d**

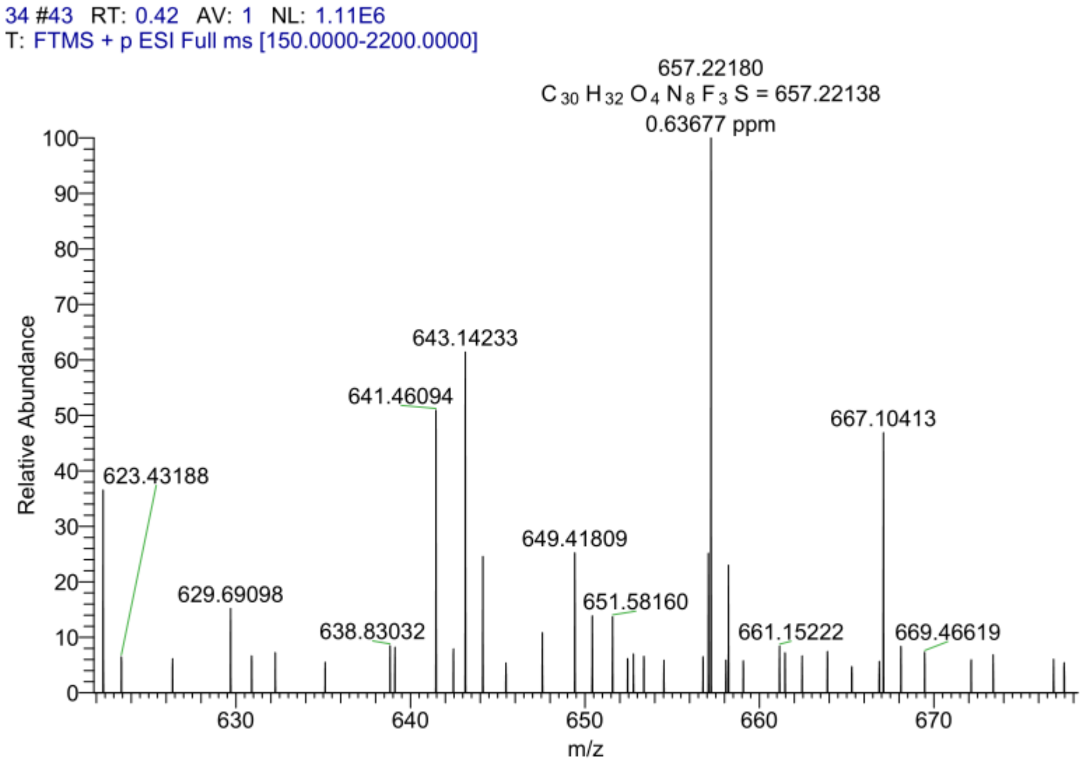

Figure S75. HRMS spectrum of compound **8d**

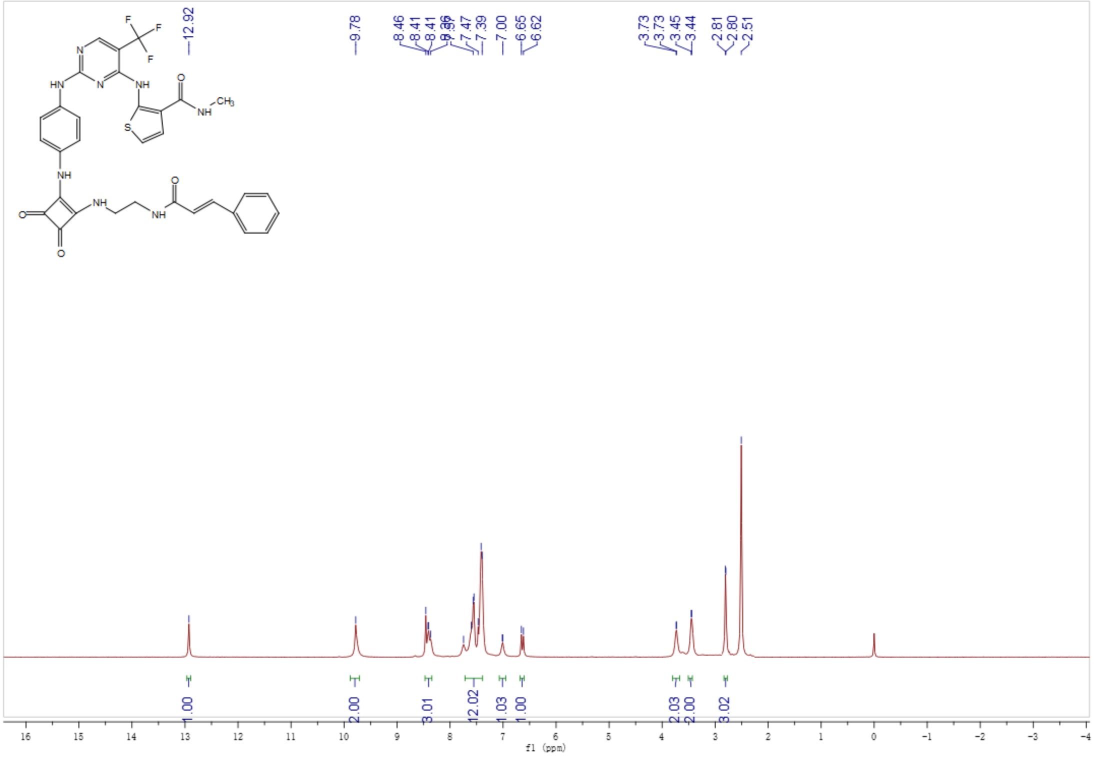

Figure S76. ^{1}H NMR spectrum of compound **8e**

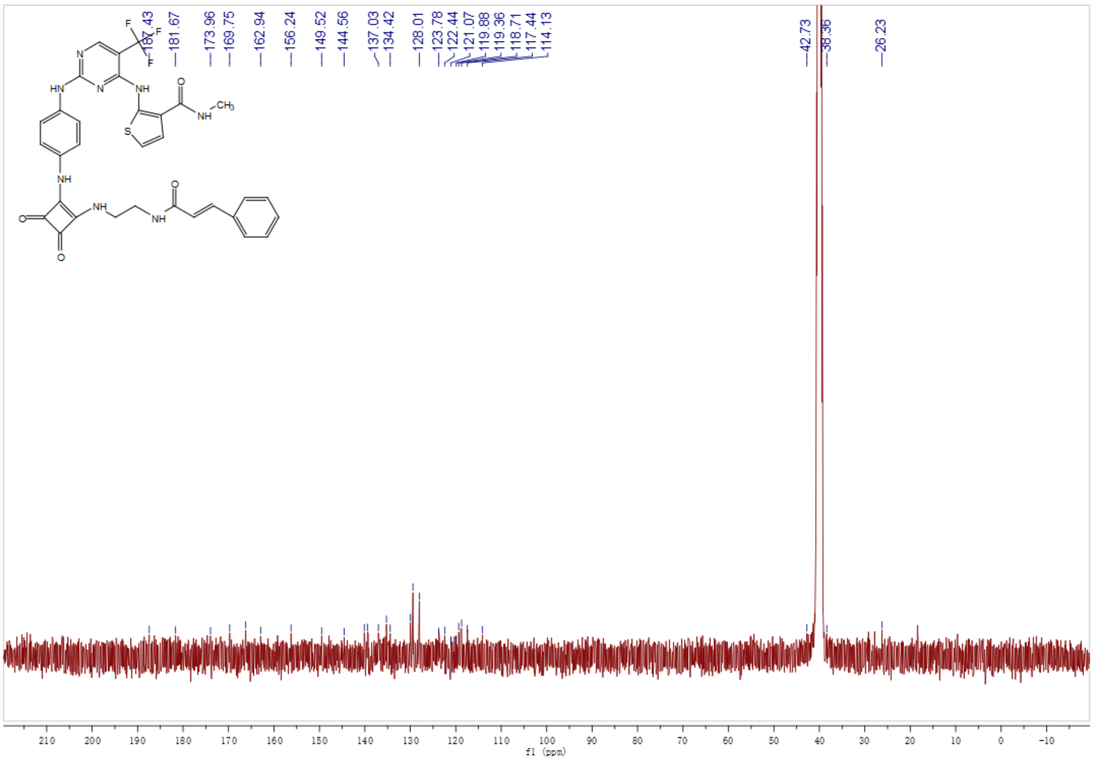

Figure S77. ^{13}C NMR spectrum of compound **8e**

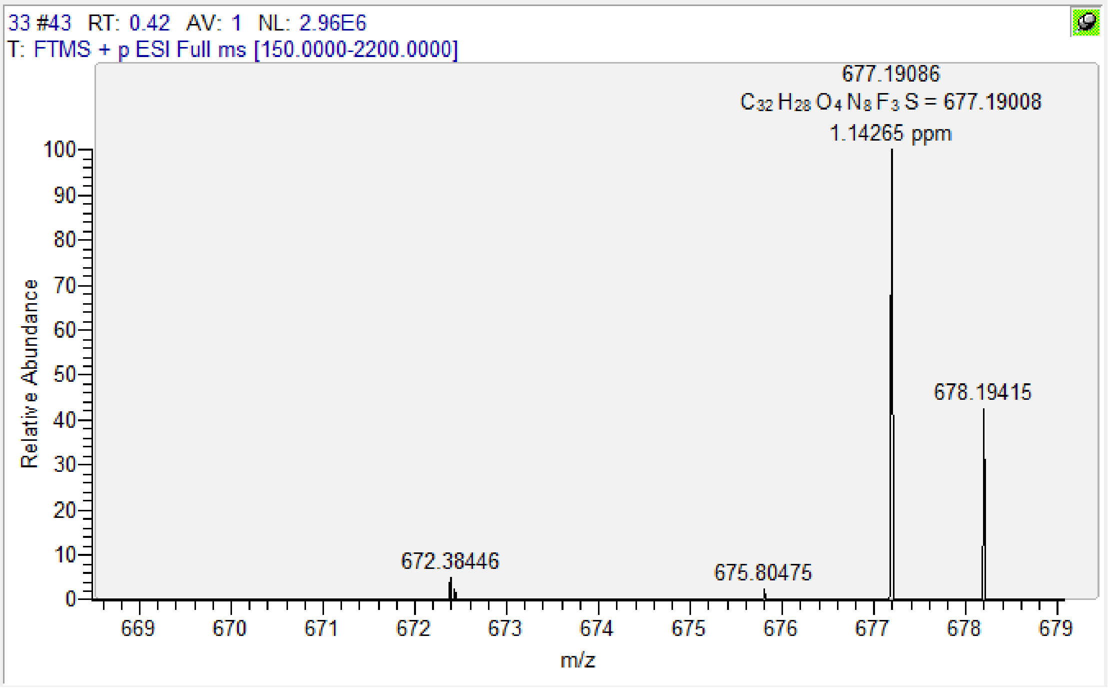

Figure S78. HRMS spectrum of compound **8e**

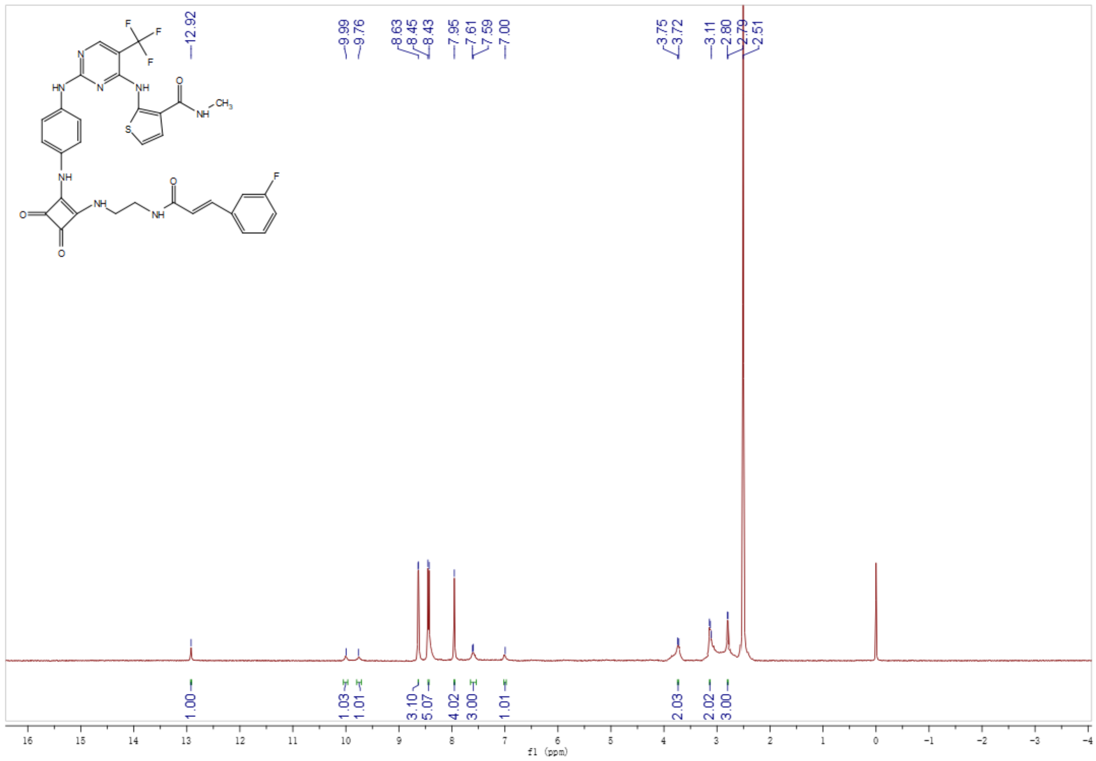

Figure S79. ^{1}H NMR spectrum of compound **8f**

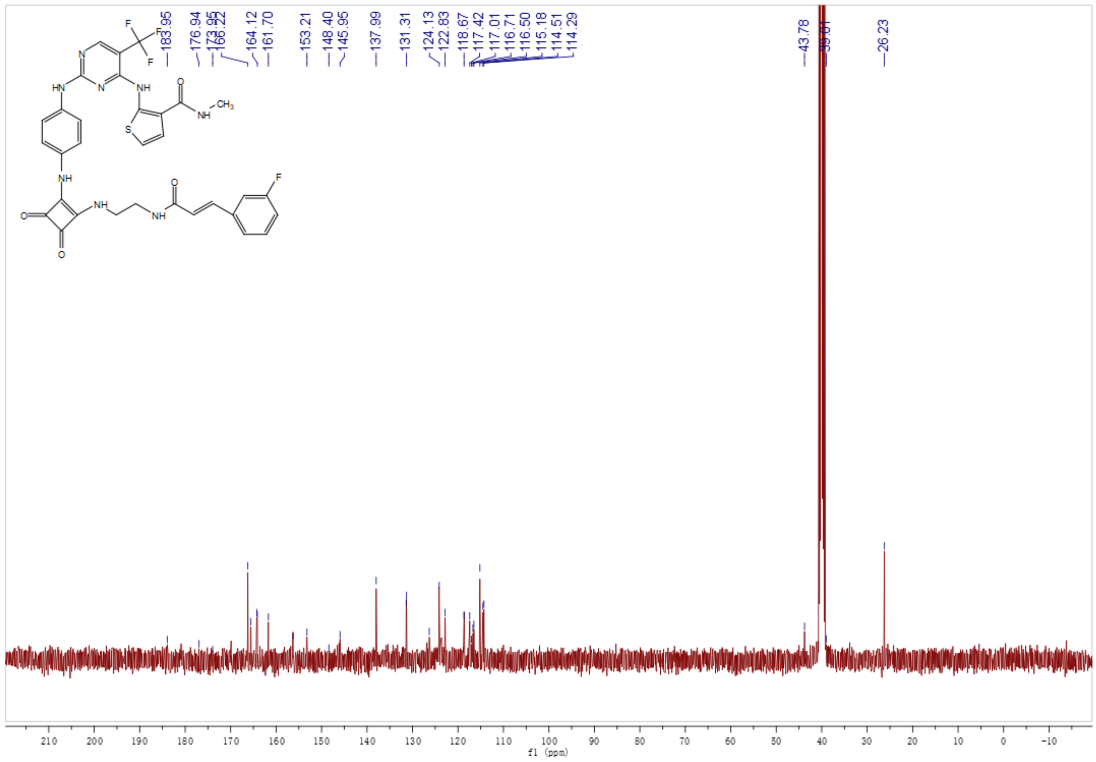

Figure S80. ^{13}C NMR spectrum of compound **8f**

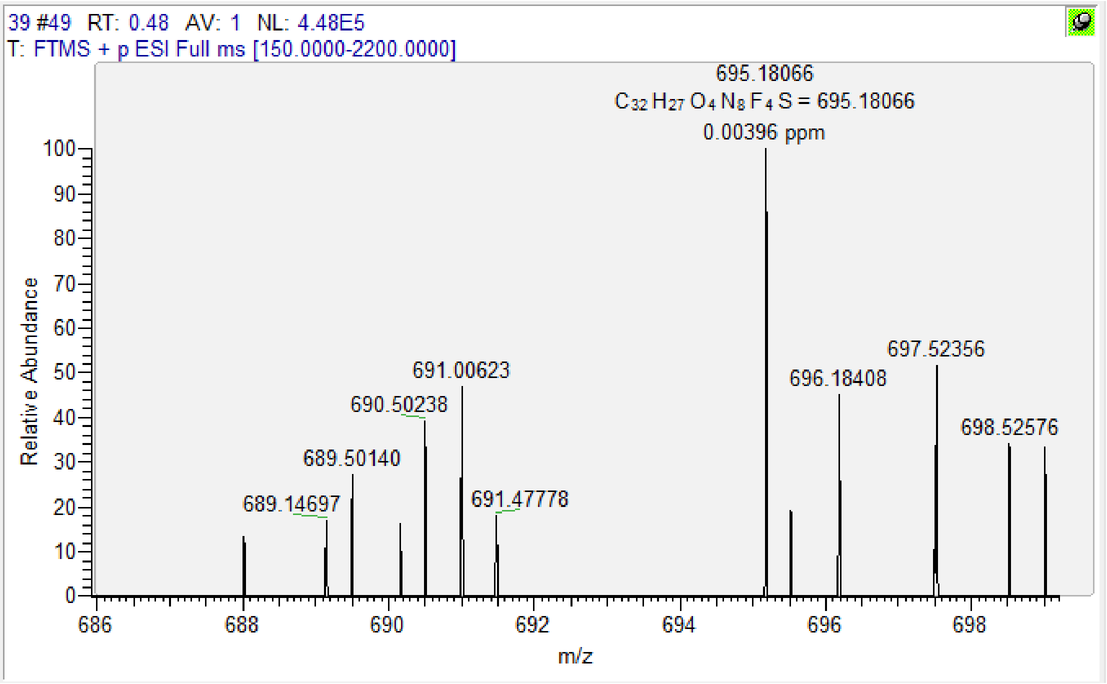

Figure S81. HRMS spectrum of compound **8f**

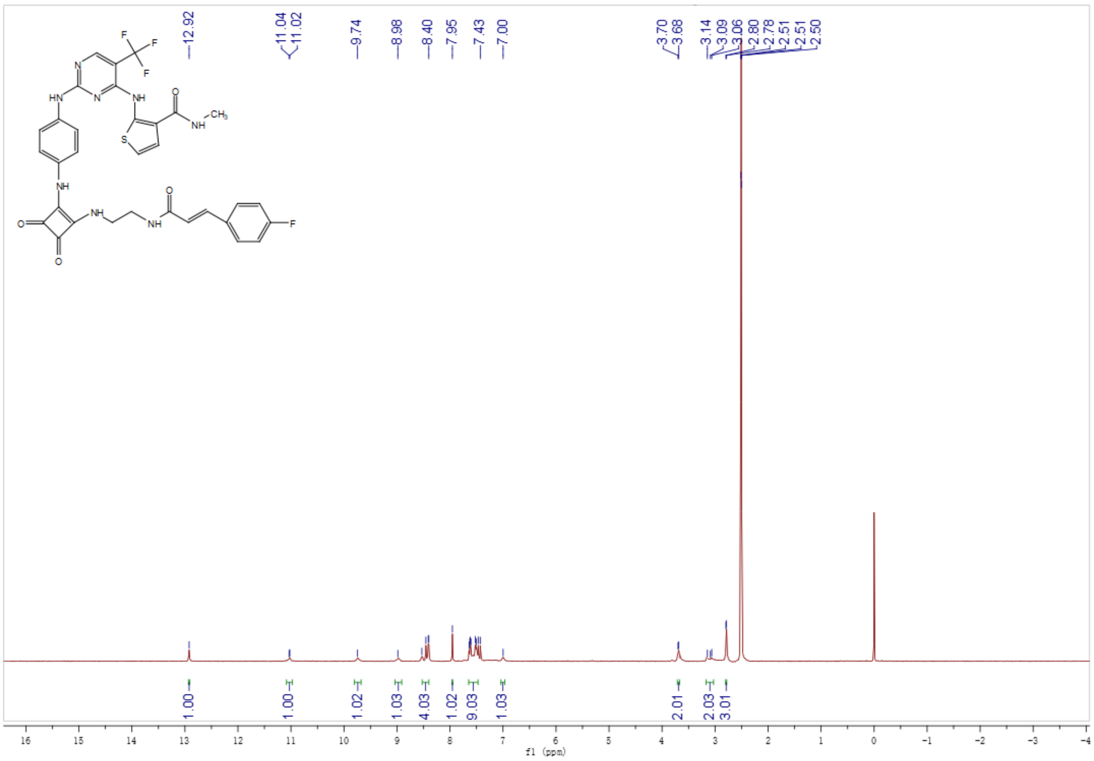

Figure S82. ^{1}H NMR spectrum of compound **8g**

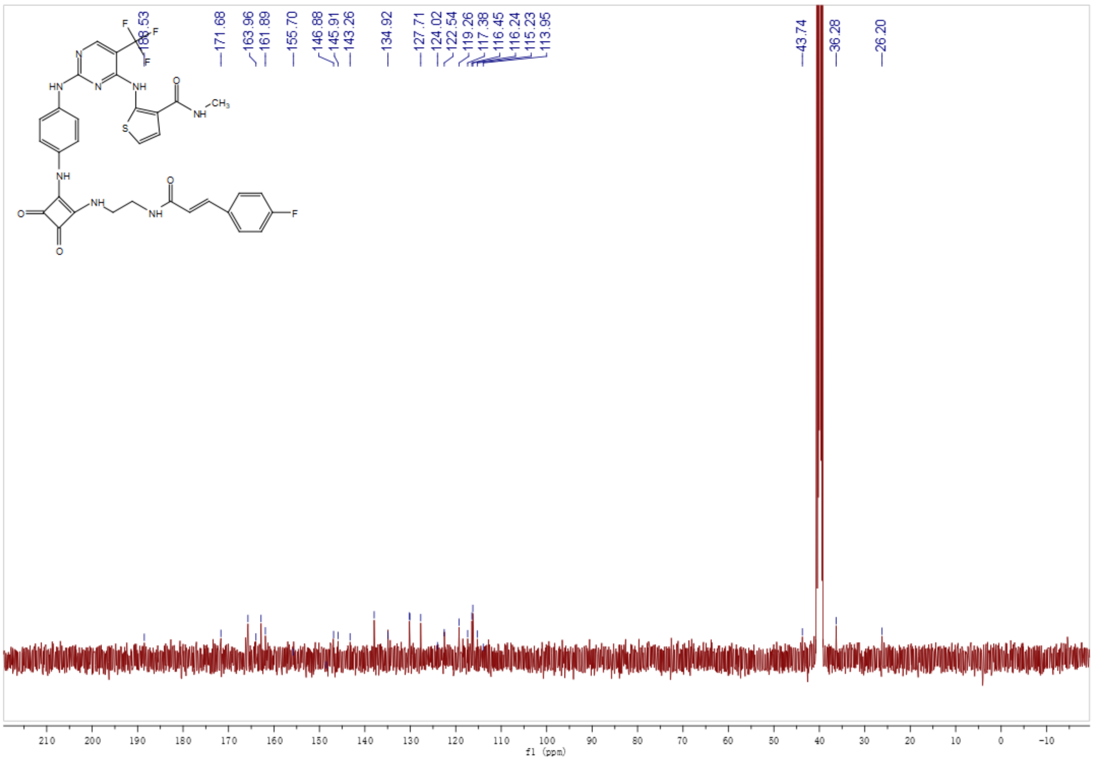

Figure S83. ^{13}C NMR spectrum of compound **8g**

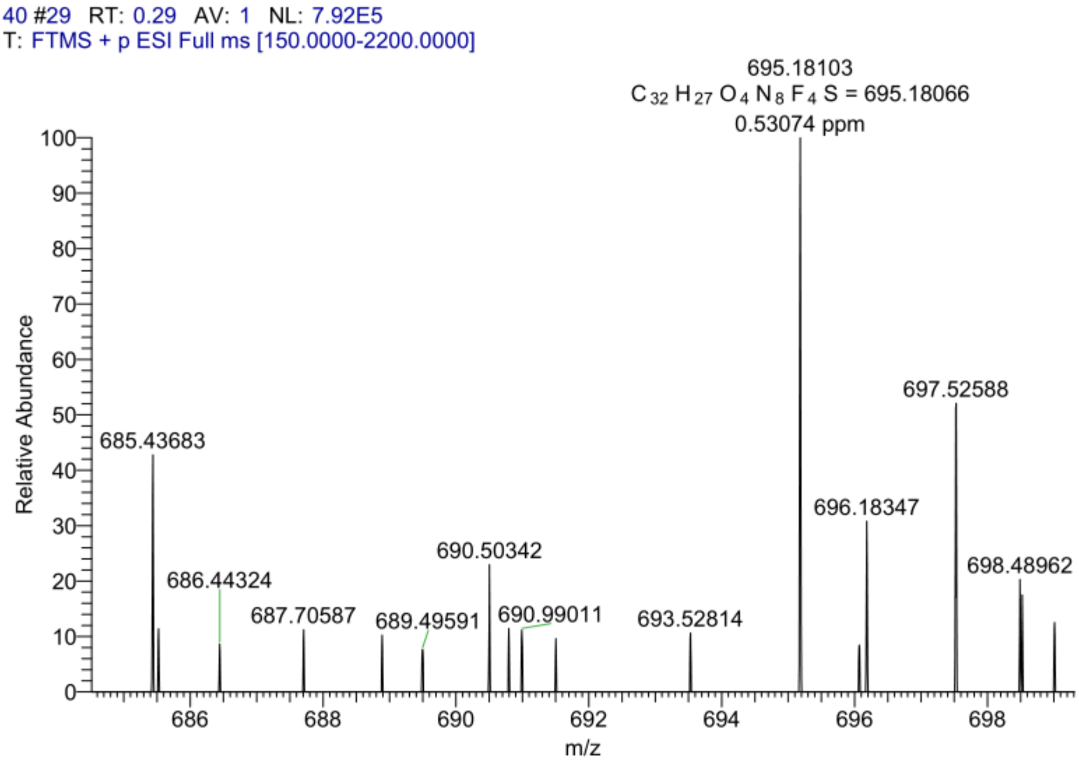

Figure S84. HRMS spectrum of compound **8g**

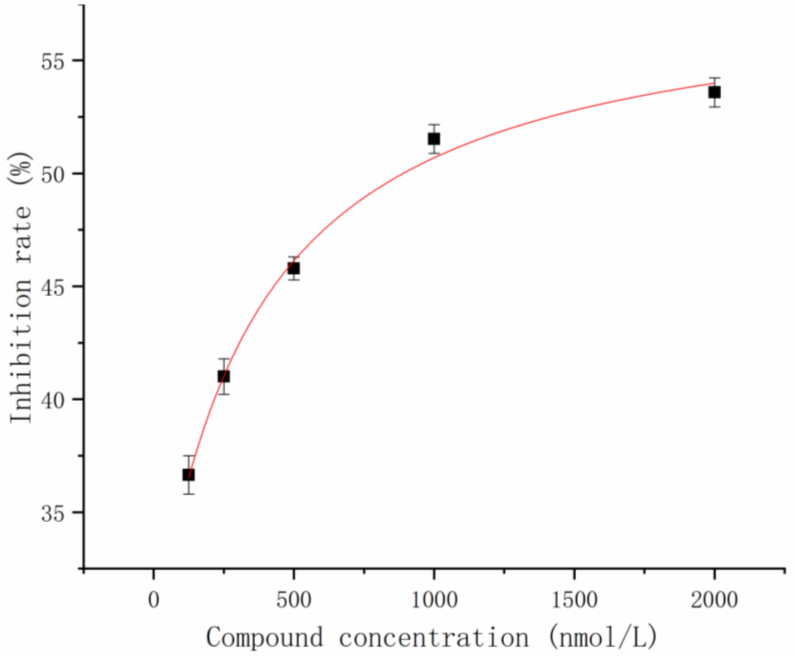

Figure S85. IC_{50} curves of compound **4b**

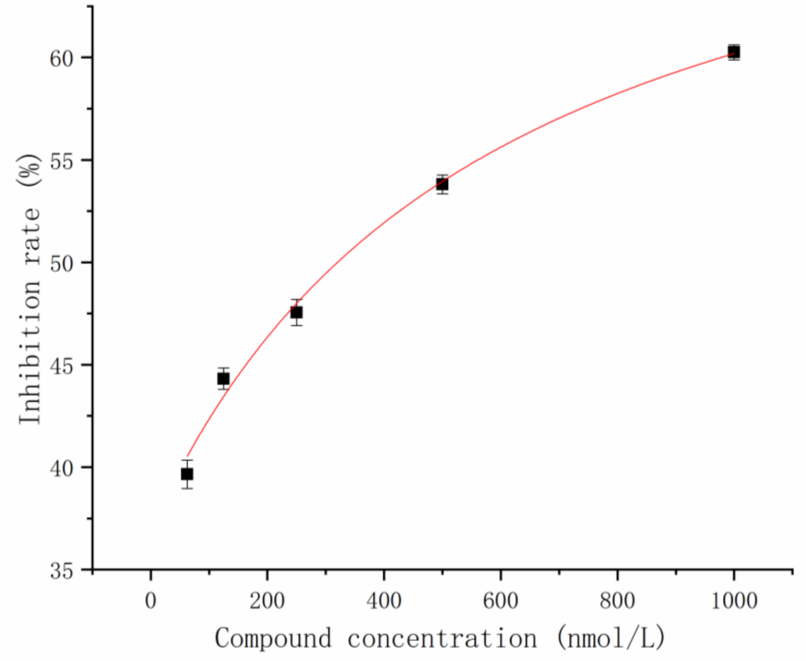

Figure S86. IC_{50} curves of compound **4c**

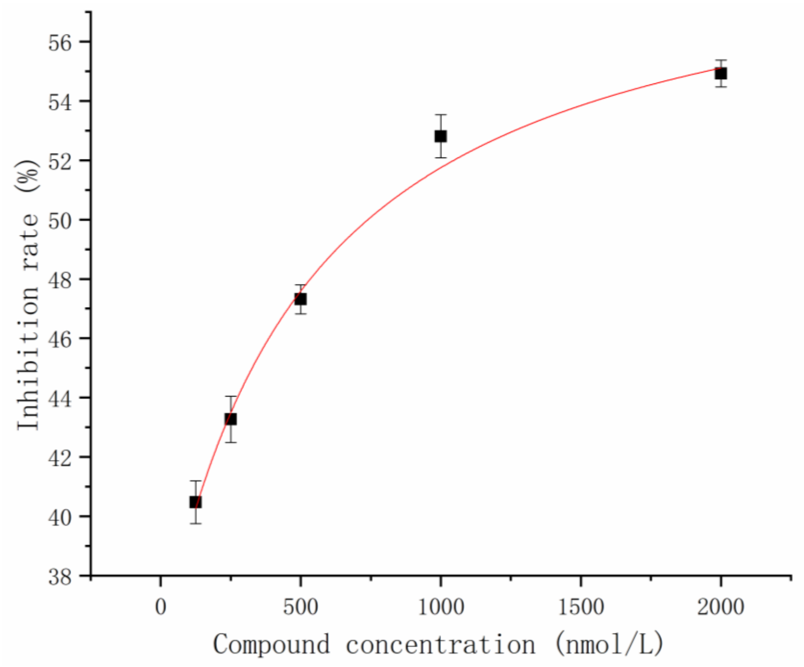

Figure S87. IC_{50} curves of compound **6e**

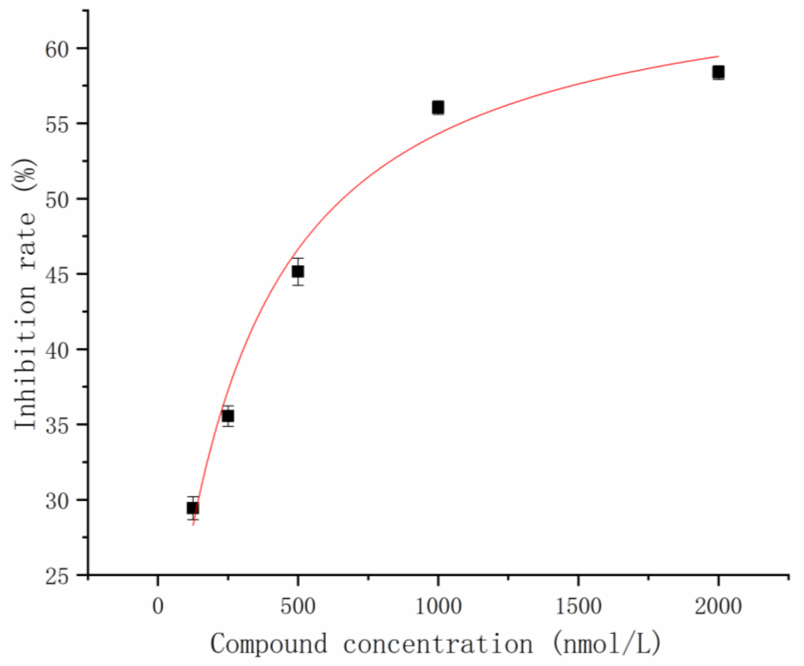

Figure S88. IC_{50} curves of compound **6i**

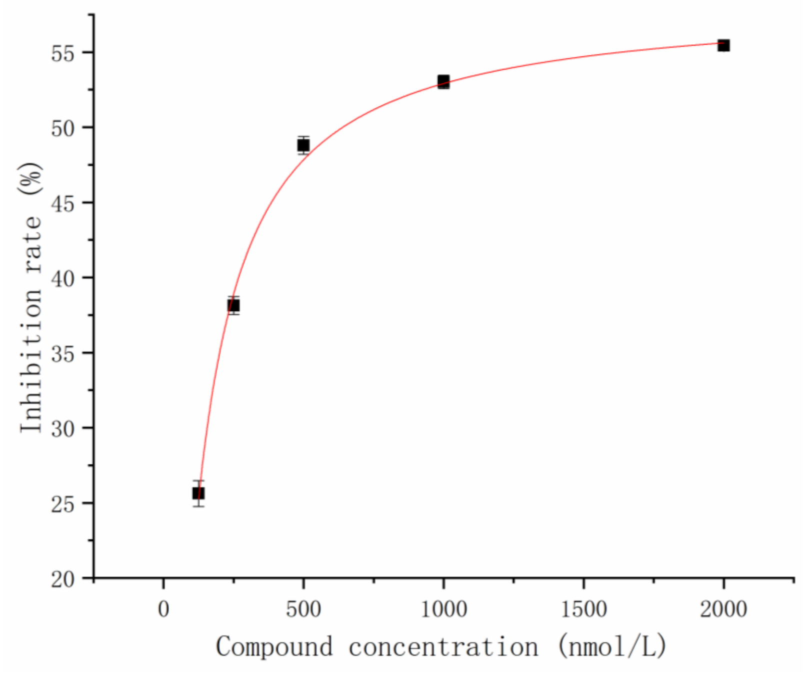

Figure S89. IC_{50} curves of compound **8e**

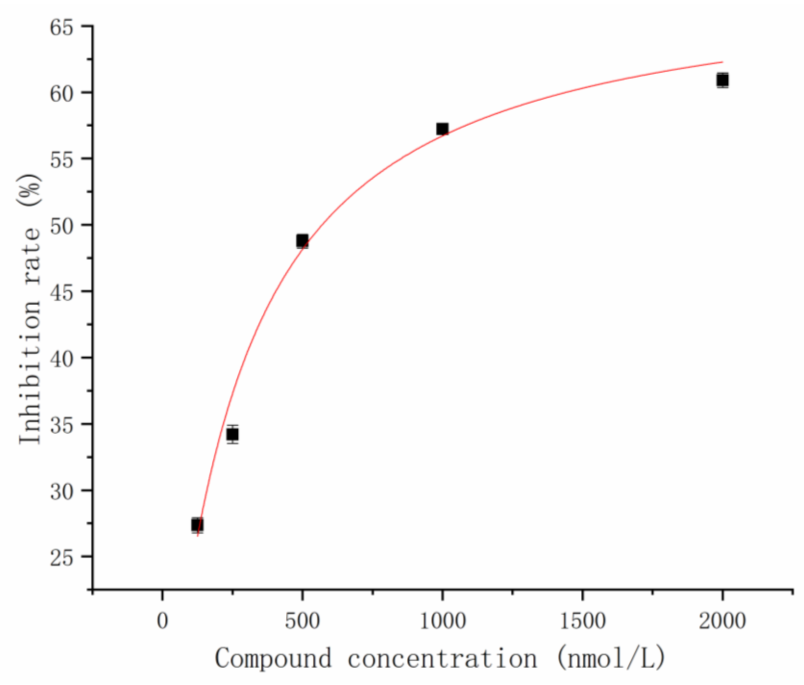

Figure S90. IC_{50} curves of compound **8f**

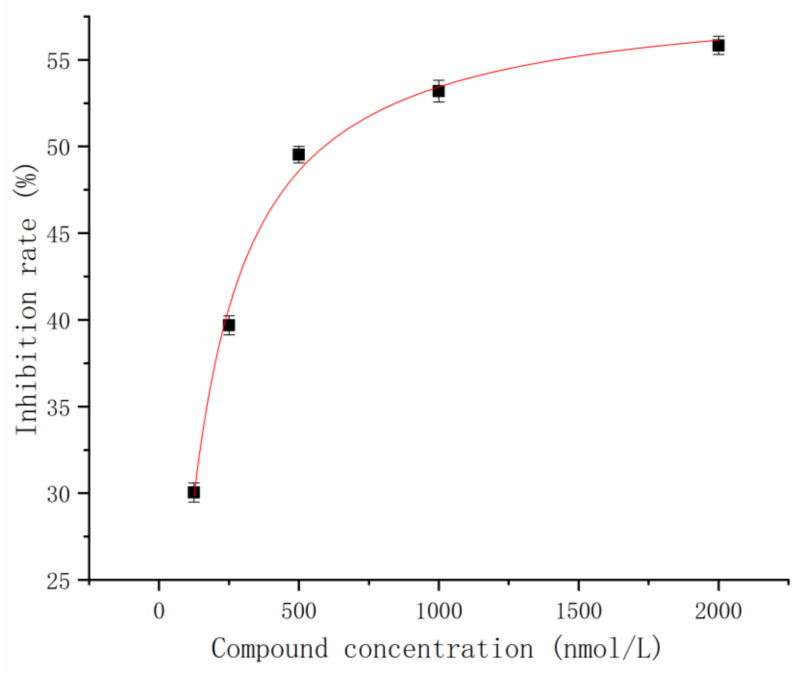

Figure S90. IC_{50} curves of compound **8g**
